# ANGPTL2 activity in cardiac pathologies accelerates heart failure by perturbing cardiac function and energy metabolism

**DOI:** 10.1038/ncomms13016

**Published:** 2016-09-28

**Authors:** Zhe Tian, Keishi Miyata, Tsuyoshi Kadomatsu, Haruki Horiguchi, Hiroyuki Fukushima, Shugo Tohyama, Yoshihiro Ujihara, Takahiro Okumura, Satoshi Yamaguchi, Jiabin Zhao, Motoyoshi Endo, Jun Morinaga, Michio Sato, Taichi Sugizaki, Shunshun Zhu, Kazutoyo Terada, Hisashi Sakaguchi, Yoshihiro Komohara, Motohiro Takeya, Naoki Takeda, Kimi Araki, Ichiro Manabe, Keiichi Fukuda, Kinya Otsu, Jun Wada, Toyoaki Murohara, Satoshi Mohri, Jun K. Yamashita, Motoaki Sano, Yuichi Oike

**Affiliations:** 1 Department of Molecular Genetics, Graduate School of Medical Sciences, Kumamoto University, Kumamoto 860-8556, Japan; 2 Department of Cardiovascular Surgery, Graduate School of Medical Sciences, Kumamoto University, Kumamoto 860-8556, Japan; 3 Department of Immunology, Allergy, and Vascular Biology, Graduate School of Medical Sciences, Kumamoto University, Kumamoto 860-8556, Japan; 4 Institute of Resource Developmental and Analysis, Kumamoto University, Kumamoto 860-8111, Japan; 5 Department of Cell Growth and Differentiation, Center for iPS Cell Research and Application (CiRA), Kyoto University, Kyoto 606-8507, Japan; 6 Department of Cardiovascular Medicine, School of Medicine, Keio University, Tokyo 160-8582, Japan; 7 First Department of Physiology, Kawasaki Medical School, Okayama 701-092, Japan; 8 Department of Cardiology, Nagoya University Graduate school of Medicine, Nagoya 466-8550, Japan; 9 Department of Nephrology, Rheumatology, Endocrinology and Metabolism, Okayama University Graduate School of Medicine, Dentistry and Pharmaceutical Sciences, Okayama 700-8558, Japan; 10 Department of Cell Pathology, Graduate School of Medical Sciences, Kumamoto University, Kumamoto 860-8556, Japan; 11 Department of Aging Research, Graduate School of Medicine, Chiba University, Chiba 260-8670, Japan; 12 Cardiovascular Division, King's College London British Heart Foundation Centre of Research Excellence, London SE5 9NU, UK

## Abstract

A cardioprotective response that alters ventricular contractility or promotes cardiomyocyte enlargement occurs with increased workload in conditions such as hypertension. When that response is excessive, pathological cardiac remodelling occurs, which can progress to heart failure, a leading cause of death worldwide. Mechanisms underlying this response are not fully understood. Here, we report that expression of angiopoietin-like protein 2 (ANGPTL2) increases in pathologically-remodeled hearts of mice and humans, while decreased cardiac ANGPTL2 expression occurs in physiological cardiac remodelling induced by endurance training in mice. Mice overexpressing ANGPTL2 in heart show cardiac dysfunction caused by both inactivation of AKT and sarco(endo)plasmic reticulum Ca^2+^-ATPase (SERCA)2a signalling and decreased myocardial energy metabolism. Conversely, *Angptl2* knockout mice exhibit increased left ventricular contractility and upregulated AKT-SERCA2a signalling and energy metabolism. Finally, ANGPTL2-knockdown in mice subjected to pressure overload ameliorates cardiac dysfunction. Overall, these studies suggest that therapeutic ANGPTL2 suppression could antagonize development of heart failure.

## Abstract (web summary)

Heart responds to increased workload by enlarging cardiomyocytes to preserve function, but in pathologies hypertrophy leads to heart failure. Here the authors show that ANGPTL2 activity in the heart is critical for determining beneficial *vs*. pathological hypertrophy via its effect on AKT-SERCA2a signaling and myocardial energy.

## Graphical abstract

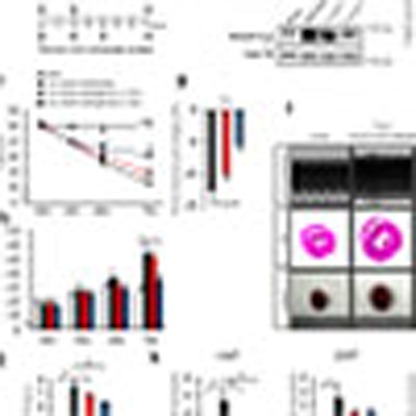

A cardioprotective response to increase left ventricular contractile ability and improve myocardial metabolism occurs with increased workload in cardiac conditions such as hypertension. When this response is excessive, pathological cardiac remodelling occurs, a condition often progressing to heart failure (HF), a leading cause of death that affects more than 23 million people worldwide^1^. HF is a cardiac dysfunction marked by perturbations in adequate oxygen supply required to maintain proper heart contractility and energy production^2^. Decreased cardiac contractility seen in HF is marked by reduced ejection fraction (HFrEF). By contrast, in HF associated with preserved ejection fraction (HFpEF), contractile function appears normal despite the presence of symptoms and the condition is thought to be caused by abnormal diastolic function^3^. Both contraction and relaxation are major energy-consuming processes^4^,^5^, suggesting that failure of myocardial energy metabolism underlies both HFrEF and HFpEF.

Cardiac hypertrophy, defined as increased heart mass caused due to cardiomyocyte enlargement, is also an adaptive response that maintains cardiac function in response to increased workload^6^,^7^,^8^. Pathological stimuli, such as hypertension, promote the transition from hypertrophy to HF, the latter condition associated with cardiac fibrosis and re-expression of a fetal cardiac gene programme^9^. By contrast, exercise induces physiological hypertrophy characterized by preserved myocardial structure and energy metabolism, protecting the heart from HF^10^,^11^,^12^. Mechanistic differences between physiological and pathological hypertrophy remain unclear.

The signalling factor angiopoietin-like protein 2 (ANGPTL2), one of eight ANGPTL proteins^13^, functions in maintenance of tissue homeostasis by inducing inflammation and angiogenesis^14^,^15^. In some contexts, ANGPTL2 overexpression promotes irreversible pathological tissue remodelling and development or progression of obesity-associated metabolic diseases, type 2 diabetes, atherosclerotic vascular diseases and some cancers^16^,^17^,^18^,^19^,^20^,^21^,^22^,^23^. In these conditions, ANGPTL2 expression increases in infiltrating immune cells or resident cells, such as adipocytes, vascular endothelial cells and tumour cells. Enhanced ANGPTL2 autocrine or paracrine signalling accelerates disease development and progression. Several studies report ANGPTL2 expression in heart^13^,^17^, although its function in those tissues has not been clarified.

Here, we report that cardiac ANGPTL2 expression increases in pathologically stressed hearts in mice and humans. Mice overexpressing ANGPTL2 in heart showed cardiac dysfunction accompanied by inactivation of AKT and sarco(endo)plasmic reticulum Ca^2+^-ATPase (SERCA)2a signalling and by decreased myocardial energy metabolism. In contrast, *Angptl2* knockout (KO) mice showed increased left ventricular contractile capacity via increases in AKT-SERCA2a signalling, myocardial energy metabolism and ATP production, all of which protected mice from HF in a pressure overload model. Furthermore, *Angptl2* KO phenotypes resembled exercise-induced hypertrophy. Accordingly, cardiac ANGPTL2 expression significantly decreased in physiological cardiac remodelling induced by endurance training in mice. Importantly, in a mouse pressure overload model, suppression of cardiac ANGPTL2 induction blocked HF development and was accompanied by increased AKT-SERCA2a signalling and improved cardiac energy metabolism. ANGPTL2-knockdown (KD) human-induced pluripotent stem cell (iPS)-derived cardiomyocytes showed induction of AKT-SERCA2a signalling and proper myocardial energy metabolism compared with control iPS-derived cardiomyocytes. Together, these results suggest that increased ANGPTL2 activity accelerates cardiac dysfunction and that therapeutic ANGPTL2 suppression could antagonize this condition.

## Results

### Elevated ANGPTL2 expression in stressed cardiomyocytes

**Figure 1:**
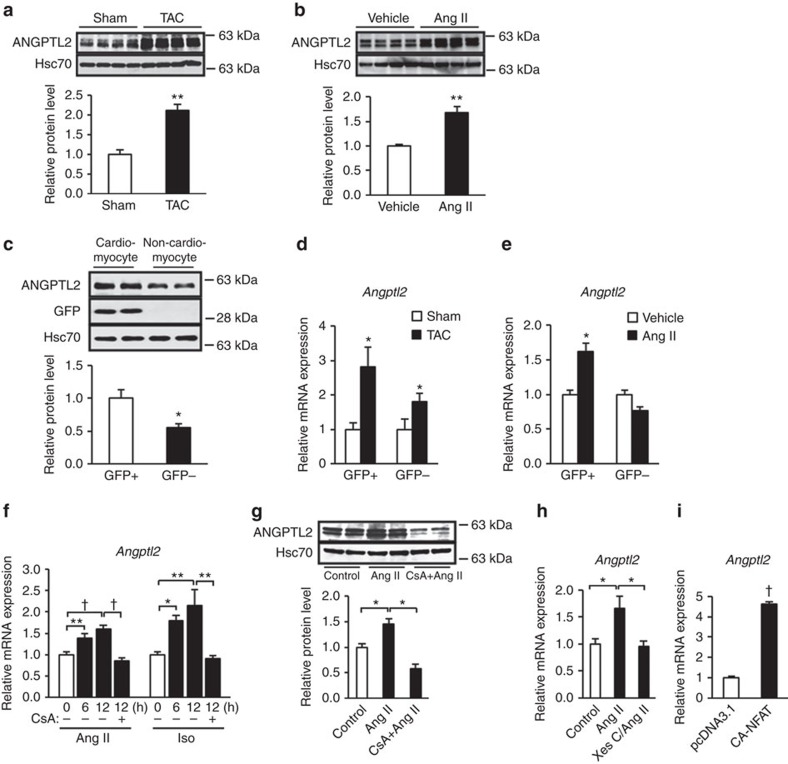
ANGPTL2 expression is increased in stressed cardiomyocytes. (**a**–**c**) Representative western blot and quantitation of ANGPTL2 protein in hearts from WT mice 6 weeks after TAC or sham surgery (*n*=5 per group) (**a**) and 2 weeks after Angiotensin II (Ang II) or vehicle treatment (*n*=8 per group) (**b**). Analysis of ANGPTL2 and GFP protein from GFP^+^ cardiomyocytes and GFP^−^ non-cardiomyocytes from *Myh6-EGFP* Tg mice (*n*=3 per group) (**c**). Hsc70 served as a loading control. Normalized values from controls (**a**,**b**) or GFP^+^ cardiomyocytes (**c**) were set to 1. (**d**,**e**) Quantitative RT-PCR analysis of *Angptl2* expression in GFP^+^ and GFP^–^ cells 6 weeks after TAC or sham surgery (**d**) and 2 weeks after Ang II or vehicle treatment (**e**) (*n*=3 per group). Values for respective controls were set to 1. (**f**,**g**) Relative ANGPTL2 transcript (**f**) and protein (**g**) levels in NRCMs assessed 6 or 12 h after Ang II (left in **f**,**g**) or Iso (right in **f**) treatment, with or without cyclosporine A (CsA) treatment. Values before treatment were set to 1. *n*=4–8 per group. (**h**) Relative ANGPTL2 transcript levels in NRCMs 8 h after Ang II treatment, with or without xestospongin C (Xes C) treatment. Values from control NRCMs were set to 1. *n*=8 per group. (**i**) Relative *Angptl2* transcript levels in NRCMs overexpressing constitutively active NFAT (CA-NFAT). Values from control NRCMs were set to 1. *n*=3 per group. Data are means±s.e.m. Statistical significance was determined by Student's *t* test (**a**–**e** and **i**) or one-way ANOVA (**f**–**h**). *P<0.05, **P<0.01, ^†^*P*<0.001 between groups.

To assess ANGPTL2 function in heart, we assessed ANGPTL2 protein levels in stressed heart tissues induced by pressure overload in wild-type (WT) mice using transverse aorta constriction (TAC). TAC animals showed higher ANGPTL2 protein levels than did sham-operated controls (Fig. 1a). TAC mice developed pathological cardiac remodelling with left ventricular dilatation, decreased fractional shortening and increased expression of the HF markers *ANP*, *BNP* and *Myh7* and cardiac fibrosis markers *CTGF*, *Col1* and *Col3a1*, and developed HFrEF caused by systolic dysfunction with left ventricular dilatation (Supplementary Fig. 1a,b).

Moreover, using an alternate model of HF based on Angiotensin II (Ang II) administration, we observed that ANGPTL2 protein levels in stressed heart tissues resulting from Ang II infusion significantly increased relative to vehicle-infusion controls (Fig. 1b). In this model, mice showed no left ventricular dilatation and preserved fractional shortening but exhibited a reduced the peak early distolic filing velocity (E-wave) per the peak atrial filing velocity (A-wave) (E/A) ratio and increased expression of HF and cardiac fibrosis markers (Supplementary Fig. 1c–e), confirming that mice in this model develop HFpEF, as reported previously^24^,^25^.

To determine what cells express ANGPTL2 in heart, we separated cardiomyocytes from non-cardiomyocytes in transgenic (Tg) mice expressing enhanced green fluorescent protein (EGFP) under control of the cardiomyocyte-specific promoter *αMHC* (*αMHC-EGFP* Tg mice). Immunoblotting revealed more abundant ANGPTL2 expression in GFP-positive cardiomyocytes than in GFP-negative cells (Fig. 1c; Supplementary Fig. 1f,g). RT-PCR analysis also revealed increased *Angptl2* expression in GFP-positive cardiomyocytes in cells from both TAC and Ang II-induced stressed hearts (Fig. 1d,e), suggesting that ANGPTL2 expression increases in stressed cardiomyocytes during pathological cardiac remodelling.

### NFAT increases *Angptl2* expression in cardiomyocytes

To identify factors governing ANGPTL2 expression in stressed heart tissues, we stimulated cultured neonatal rat cardiomyocytes (NRCMs) with Ang II or isoproterenol (Iso), drugs known to induce pathological remodelling. Treatment with either significantly increased *Angptl2* mRNA levels in NRCMs (Fig. 1f). Both Ang II and Iso reportedly promote nuclear translocation of NFAT^26^,^27^, a key transcription factor underlying development of pathological cardiac remodelling^28^, and we previously reported that the presence of a complex of NFAT, ATF2 and c-jun is associated with increased *Angptl2* transcription in lung and breast tumour cells^22^. Immunocytochemical analysis confirmed that NFATC1 and NFATC4, both expressed in cardiomyocytes^29^, undergo nuclear translocation after Ang II treatment, an activity inhibited by cyclosporine A (CsA), which inhibits the calcium-dependent protein phosphatase calcineurin (Supplementary Fig. 2a). CsA treatment also blocked Ang II- or Iso-dependent *Angptl2* upregulation in NRCMs (Fig. 1f). Ang II-stimulated increases in ANGPTL2 protein levels in NRCMs were blocked by CsA treatment (Fig. 1g). Moreover, treatment of NRCMs with xestospongin C (Xes C), an IP3 receptor antagonist that blocks elevation of cytosolic calcium concentration, inhibited Ang II-induced *Angptl2* expression (Fig. 1h). In addition, overexpression of constitutively active NFAT (CA-NFAT) increased *Angptl2* mRNA levels in NRCMs (Fig. 1i). Finally, immunocytochemical analysis revealed that NFATC4 underwent nuclear translocation in stressed heart tissue following either TAC or Ang II infusion (Supplementary Fig. 2b), suggesting that calcineurin-NFAT signalling increases ANGPTL2 expression in stressed heart in both contexts.

### Elevated ANGPTL2 impairs cardiomyocyte contractility

**Figure 2:**
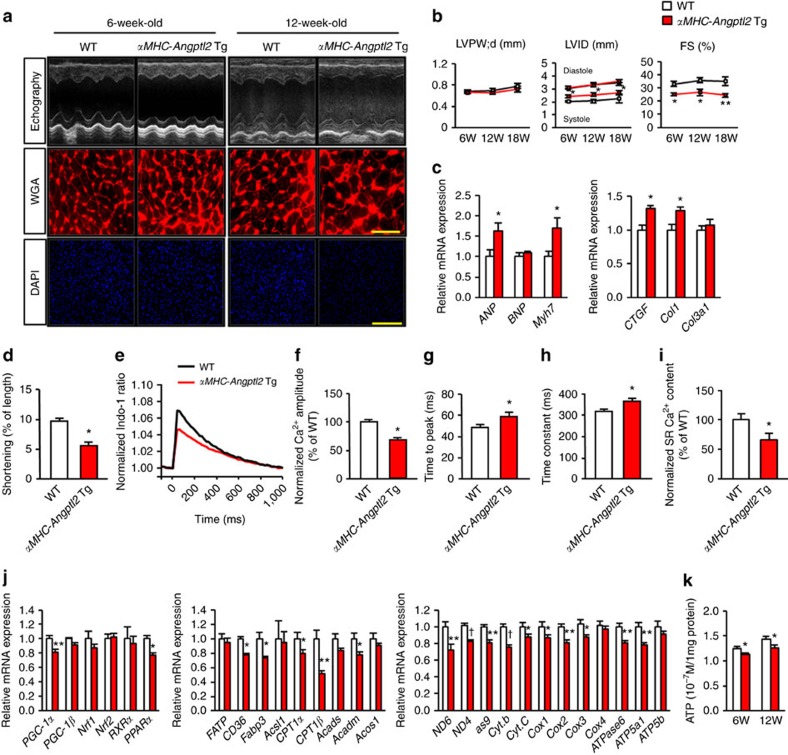
Cardiomyocyte-derived ANGPTL2 induces cardiac dysfunction in mice. (**a**) Shown are representative M-mode echocardiography recordings (upper row), left ventricle sections stained with wheat germ agglutinin (WGA; as an indicator of cardiomyocyte size) (middle, Scale bars, 50 μm) and DAPI staining (lower, Scale bars, 200 μm) of 6- and 12-week-old *αMHC-Angptl2* Tg and littermate WTWT mice. (**b**) Diastolic left ventricular posterior wall thickness (LVPW;d), left ventricular end-diastolic internal diameter (LVID;d) and percent fractional shortening (%FS) in 6-, 12- and 18-week-old *αMHC-Angptl2* Tg and WT mice (*n*=5–7 per group). (**c**) Relative expression of genes associated with HF and fibrosis in hearts of 12-week-old *αMHC-Angptl2* Tg relative to littermate mice. WT values were set to 1 (*n*=5–7 per group). (**d**) Percentage of shortening of cardiomyocytes isolated from *αMHC-Angptl2* Tg (*n*=31, *N*=3) and littermate WT mice (*n*=40, *N*=3). (**e**) Mean Ca^2+^ transients at 1 Hz stimulation, (**f**) peak amplitude of Ca^2+^ transients, (**g**) time to peak amplitude of Ca^2+^ transients, (**h**) decay time constant and (**i**) mean SR Ca^2+^ content in cardiomyocytes from *αMHC-Angptl2* Tg (*n*=54, *N*=3) and WT littermate mice (*n*=35, *N*=3). (**j**) Relative expression of genes associated with energy metabolism (left), β-fatty acid oxidation (middle) and mitochondrial biogenesis (right) in hearts of 10-week-old *αMHC-Angptl2* Tg and WT mice. WT values were set to 1. *n*=7–9 per group. (**k**) ATP levels in hearts of 6- and 12-week-old *αMHC-Angptl2* Tg and WT mice. *n*=7-9 per group for 6-week-old mice, *n*=11 per group for 12-week-old mice. Data are means±s.e.m. Statistical significance was determined by Student's *t* test. *P<0.05, **P<0.01, ^†^*P*<0.001 between genotypes. N, number of independent experiments.

We next asked whether increased levels of ANGPTL2 proteins alter cardiac physiology. To do so, we generated four independent Tg mouse lines overexpressing ANGPTL2 in cardiomyocytes under control of the *αMHC* promoter (*αMHC-Angptl2* Tg mice) (Supplementary Fig. 3a,b). All mice appeared normal at birth and showed no gross perturbations in cardiac development. Three lines (#1-9, #2-3 and #2-14) showed increased ANGPTL2 expression in heart comparable to that seen in TAC-induced pathological remodelling (Fig. 1a; Supplementary Fig. 3b) and two (#1-9 and #2-3) were chosen for further analysis. Compared with WT littermates, 6-week-old *αMHC-Angptl2* Tg mice of both lines showed decreased cardiac systolic contractile ability without histological cardiomyocyte enlargement. Twelve-week-old Tg mice exhibited cardiac systolic dysfunction and showed histological cardiomyocyte enlargement and increased expression of *ANP* and *Myh7* and *CTGF* and *Col1* compared with WT littermates of both lines (Fig. 2a–c; Supplementary Fig. 3c,d for representative results in #1-9). Although cardiomyocyte enlargement was seen in 12-week-old Tg mice of both lines, left ventricular (LV) wall thickness as evaluated by ultrasonic echocardiography was not present (Fig. 2a,b), suggesting that increased ANGPTL2 levels in heart primarily attenuate cardiac contractile capacity.

Cardiac contractile dysfunction is associated with abnormal Ca^2+^ handling in cardiomyocytes^30^. To assess excitation contraction (E-C) coupling of cardiomyocytes overexpressing ANGPTL2, we analyzed contractility and Ca^2+^ transients induced by electrical stimulation at 1 Hz in single cells isolated from *αMHC-Angptl2* Tg (#1-9) and WT control mice. Fractional shortening was markedly reduced in cardiomyocytes overexpressing ANGPTL2 relative to controls (Fig. 2d). In addition, in ANGPTL2-overexpressing cardiomyocytes, the magnitude of electrically evoked Ca^2+^ transients was 68% that of WT values (Fig. 2e,f). The time to peak [Ca^2+^]_i_ and the time constant of Ca^2+^ transient decay were prolonged relative to controls in ANGPTL2-overexpressing cardiomyocytes (Fig. 2e,g,h). Moreover, sarcoplasmic reticulum (SR) Ca^2+^ content in cardiomyocytes from *αMHC-Angptl2* Tg mice was significantly lower relative to that in WT littermates (Fig. 2i; Supplementary Fig. 3e). These findings indicate that increased ANGPTL2 expression impairs cardiomyocyte contractility and Ca^2+^ cycling.

Cardiac function requires constant intracellular ATP production^31^,^32^. Because *αMHC-Angptl2* Tg mice exhibit attenuated cardiac contractility, we compared expression of genes regulating myocardial energy metabolism in *αMHC-Angptl2* Tg and WT controls. Expression of energy-related genes, including *PGC-1α* and *PPARα* (both of which regulate β-oxidation and mitochondrial biogenesis)^33^,^34^, as well as genes that function in intracellular ATP production was significantly decreased in *αMHC-Angptl2* Tg (Fig. 2j,k), suggesting that increased ANGPTL2 expression in cardiomyocytes is associated with altered mitochondrial energy metabolism.

### Elevated cardiac ANGPTL2 predisposes to cardiac dysfunction

**Figure 3:**
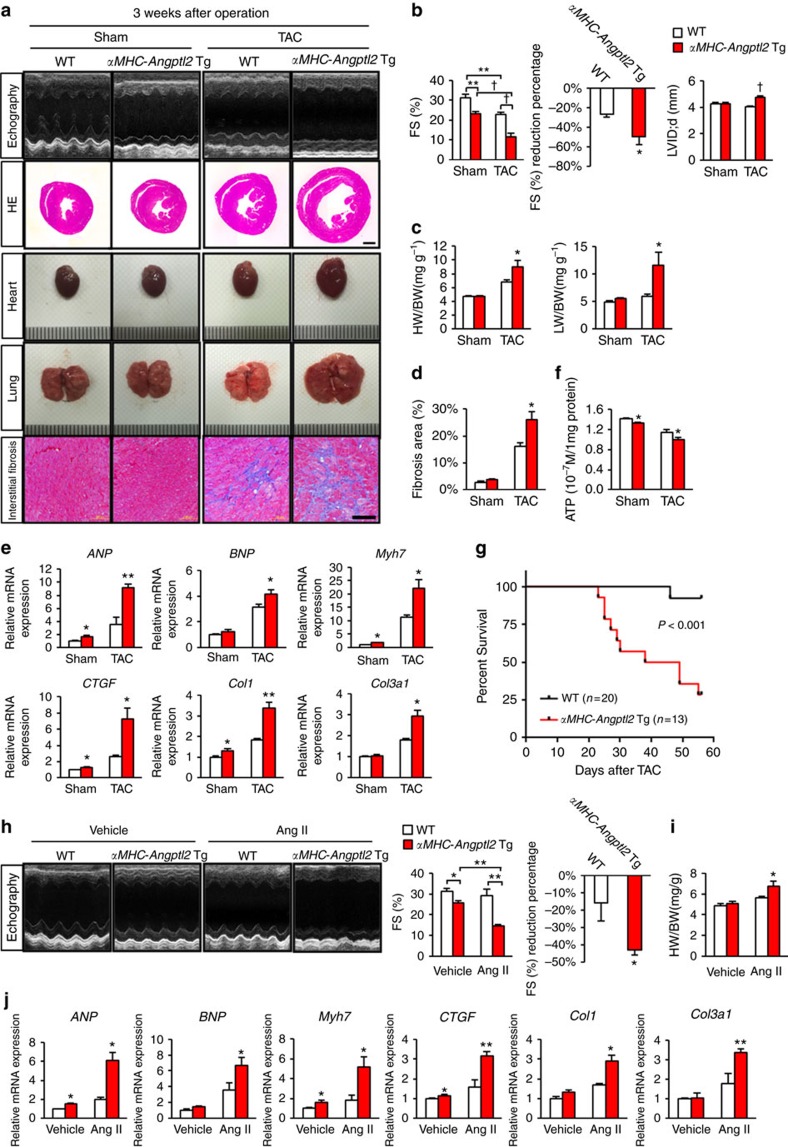
ANGPTL2-overexpressing mice are predisposed to HF. (**a**) Shown are representative M-mode echocardiography recordings (top row), HE-stained sections of heart mid-portion (second row, Scale bar, 1 mm), gross appearance of whole heart (third row) and lung (fourth row) and sections of Masson's Trichrome-stained heart tissue (bottom row, Scale bar, 100 μm) from *αMHC-Angptl2* Tg and WT littermate mice 3 weeks after TAC or sham surgery. Ruler tick marks represent 1 mm. (**b**–**e**) Comparison of heart tissue from *αMHC-Angptl2* Tg and WT littermate mice 3 weeks after TAC or sham surgery. (**b**) FS (%), reduction percentage of FS (%), and LVID;d. (**c**) Heart weight per body weight (HW/BW) ratio and lung weight per body weight (LW/BW) ratio. (**d**) Percentage of fibrosis area. (**e**) Relative expression of genes associated with HF and cardiac fibrosis. WT values were set to 1. *n*=6–10 per group. (**f**) ATP levels in hearts of *αMHC-Angptl2* Tg and WT mice 3 weeks after TAC or sham surgery. *n*=6–11 per group. (**g**) Kaplan–Meier curve analysis following TAC surgery in *αMHC-Angptl2* Tg (*n*=13) compared with WT (*n*=28) mice. *P*<0.001 between genotypes by log-rank test. (**h**) Representative M-mode echocardiography recordings (left), %FS (middle) and percent reduction of FS (right) in *αMHC-Angptl2* Tg and WT littermate mice 2 weeks after Ang II or vehicle treatment (*n*=4–5 per group). (**i**) HW/BW ratio from *αMHC-Angptl2* Tg and littermate mice 2 weeks after Ang II or vehicle treatment (*n*=4–5 per group). (**j**) Relative expression of markers of HF and cardiac fibrosis in heart of *αMHC-Angptl2* Tg and WT littermate mice 2 weeks after Ang II or vehicle treatment (*n*=4–5 per group). Levels seen in vehicle-treated WT mice were set to 1. Data are means±s.e.m. Statistical significance was determined by Student's *t* test (**c**–**f**,**i**,**j**, percent reduction of FS in **b** and **h**, and LVID;d in **b**) or two-way ANOVA (%FS in **b** and **h**). *P<0.05, **P<0.01, ^†^*P*<0.001 between genotypes or groups.

To assess a potential function for high ANGPTL2 levels seen in pathologically-remodeled hearts, we performed TAC in *αMHC-Angptl2* Tg mice and asked whether animals were predisposed to cardiac dysfunction. Three weeks after TAC, WT mice developed adaptive cardiac hypertrophy without left ventricular dilatation (Fig. 3a–c). By contrast, *αMHC-Angptl2* Tg mice developed marked left ventricular dilatation with an advanced decrease in fractional shortening, resulting in HFrEF development accompanied by lung congestion (Fig. 3a–c; Supplementary Fig. 3f). Expression levels of HF and fibrosis markers, as estimated by RT-PCR analysis of heart tissue, significantly increased in *αMHC-Angptl2* Tg relative to control TAC mice (Fig. 3e). Fibrosis area, as estimated by histological analysis of heart tissue, significantly increased in *αMHC-Angptl2* Tg relative to control TAC mice (Fig. 3a,d). After TAC, intracellular ATP production significantly decreased in *αMHC-Angptl2* Tg relative to WT TAC mice (Fig. 3f). By 8 weeks after TAC, about 70% of *αMHC-Angptl2* Tg mice had died, while only 10% of control mice had died (Fig. 3g). Moreover, in a model of HFpEF based on Ang II administration, ultrasonic echocardiography revealed that WT mice showed preserved cardiac systolic function, as estimated by fractional shortening analysis, while *αMHC-Angptl2* Tg mice developed HFrEF with severe cardiac systolic dysfunction caused by decreased fractional shortening (Fig. 3h). In addition, heart weight markedly increased relative to control WT mice (Fig. 3i), as did expression of markers of HF and fibrosis in *αMHC-Angptl2* Tg following Ang II administration (Fig. 3j).

We next asked whether excess cardiac ANGPTL2 expression could antagonize exercise-induced cardioprotection by comparing endurance capacity following running exercise in *αMHC-Angptl2* Tg and WT littermates. In the course of testing, we eliminated 5 of 8 *αMHC-Angptl2* Tg mice chosen for analysis as they were unable to sustain running due to exhaustion, whereas all WT littermates could perform the exercise (Supplementary Fig. 3g). These findings strongly suggest that cardiac remodelling in *αMHC-Angptl2* Tg mice is pathological rather than physiological.

### Endurance exercise training suppresses ANGPTL2 expression

We confirmed that exercise-trained WT mice exhibit cardiomyocyte enlargement (Supplementary Fig. 4a) and increases in cardiac systolic contractile ability, while their expression of heart and fibrosis markers decreased relative to sedentary controls (Supplementary Fig. 4b,c). Moreover, cardiac expression of *PGC-1α* and *PPARα* in exercise-trained mice showed a relative increase (Supplementary Fig. 4d), findings in agreement with the idea that cardiac hypertrophy in this context is not pathological.

**Figure 4:**
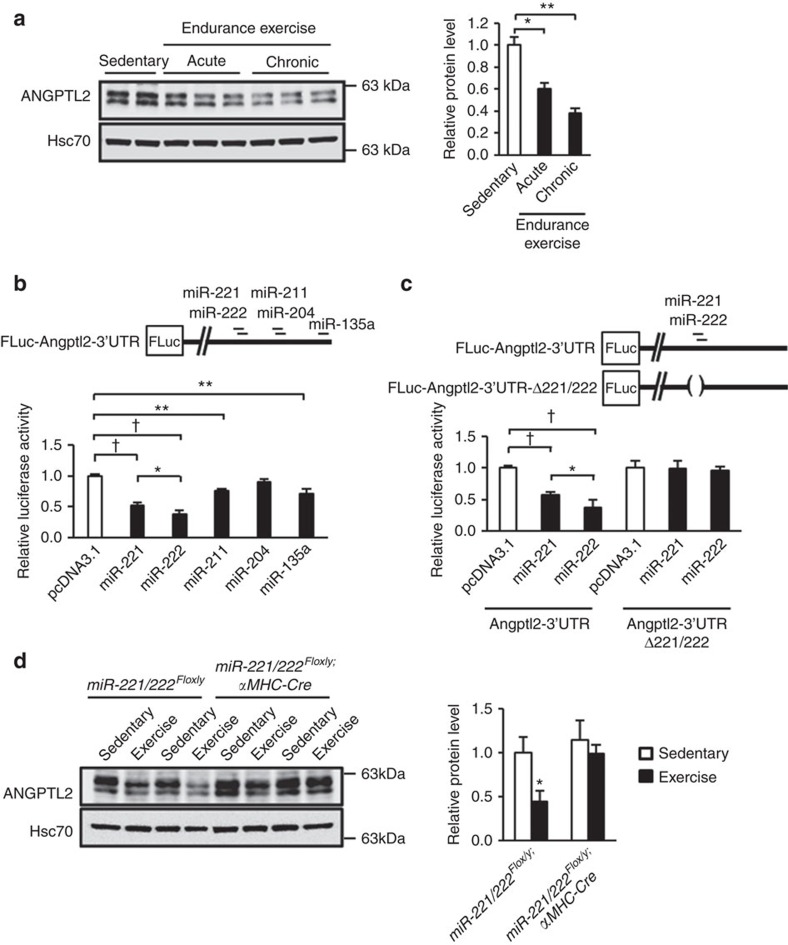
Endurance exercise training decreases ANGPTL2 expression in heart. (**a**) Representative immunoblotting and quantitation of ANGPTL2 protein from hearts of sedentary versus acute or chronic endurance-trained WT mice. Hsc70 served as a loading control. Values from sedentary WT mice are set to 1. *n*=6–8 per group. (**b**) Predicted miR-221, miR-222, miR-211, miR-204 and miR-135a binding sites in the mouse *Angptl2*-3′UTR in the FLuc-Angptl2-3′UTR reporter construct. Relative luciferase activity in NRCMs harbouring the reporter and transfected with control pcDNA3.1 vector or miR-221, miR-222, miR-211, miR-204 or miR-135a expression vectors. (**c**) Schematic showing reporter constructs with or without mutated miR-221/222 binding sites (upper). (Lower) relative luciferase activity in NRCMs harbouring WT or mutant reporter constructs and transfected with control or miR-221 or miR-222 expression vectors. Values from NRCMs transfected with vector alone were set to 1. *n*=10–12 per group. (**d**) Representative western blot (left) and quantification (right) of ANGPTL2 in heart of control and *miR-221/222* KO mice after chronic exercise training (*n*=7–8 per group). Hsc70 served as loading control. Levels in control mice in the sedentary group were set at 1. Data are means±s.e.m. Statistical significance was determined by Student's *t* test (**d**) or one-way ANOVA (**a**–**c**). *P<0.05, **P<0.01, ^†^*P*<0.001 between groups.

Interestingly, compared with outcomes seen in sedentary control mice, ANGPTL2 protein levels markedly decreased in hearts of mice undergoing acute treadmill training, and these decreases persisted in hearts of mice undergoing chronic training (Fig. 4a). To assess underlying mechanisms, we asked whether microRNAs might regulate ANGPTL2 expression in heart. Analysis of the microRNA.org database identified five candidates, including miR-135a, miR-204, miR-211, miR-221 and miR-222, predicted to bind to the *Angptl2* mRNA 3′UTR. We observed that miR-221 or miR-222 overexpression in NRCMs significantly attenuated activity of a luciferase reporter fused to the *Angptl2*-3′UTR (Fig. 4b), an activity blocked by deletion of miR-221/222 binding sequences from the UTR (Fig. 4c).

To determine whether miR-221/222 suppresses ANGPTL2 expression *in vivo*, we generated cardiomyocyte-specific miR-221/222 KO male mice by crossing *miR-221/222*^*Flox/y*^ mice with *αMHC-Cre* mice. We confirmed that *miR-221* and *miR-222* expression levels in heart of these were significantly decreased relative to control *miR-221/222*^*Flox/y*^ mice (Supplementary Fig. 5a). When we subjected *miR-221/222* KO and *miR-221/222*^*Flox/y*^ control mice to chronic exercise training, we found that ANGPTL2 protein levels in heart tissues of exercised *miR-221/222*^*Flox/y*^ control mice were significantly lower than that seen in sedentary *miR-221/222*^*Flox/y*^ controls (Fig. 4d). In contrast, an exercise-induced decrease in cardiac ANGPTL2 protein levels did not occur in hearts of exercised *miR-221/222* KO mice (Fig. 4d).

### *Angptl2* KO mice resist HF development

**Figure 5:**
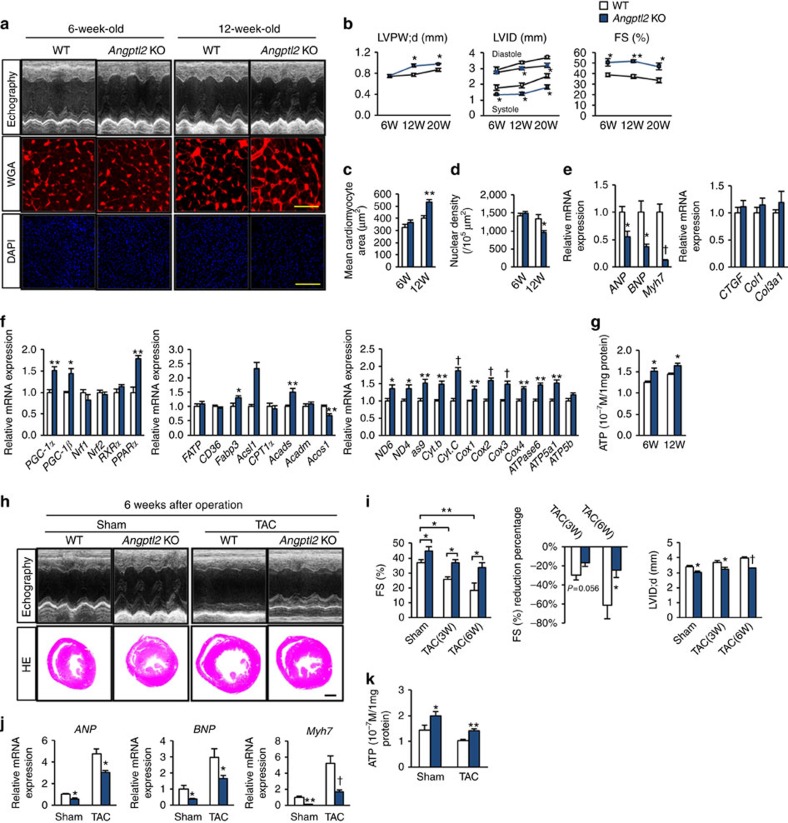
*Angptl2* KO mice show resistance to HF development. (**a**) Shown are representative M-mode echocardiography recordings (upper), WGA-stained left ventricle sections (middle, Scale bar, 50 μm) and DAPI staining (lower, Scale bar, 200 μm). (**b**–**d**) Quantitation of LVPW;d, LVID;d, and %FS in 6-, 12- and 20-week-old *Angptl2* KO and WT littermate mice (**b**) and cardiomyocyte cell size (**c**) and nuclear density (**d**) in tissues from 6- and 12-week-old *Angptl2* KO and WT littermate mice. (**e**) Relative expression of genes associated with HF and fibrosis in hearts of 12-week-old *Angptl2* KO compared with WT littermates. WT values were set to 1. (**f**) Relative expression of genes associated with energy metabolism (left), β-fatty acid oxidation (middle) and mitochondrial biogenesis (right) in hearts of 15-week-old *Angptl2* KO and WT mice. WT values were set to 1. (**g**) Quantitation of ATP levels in hearts of 6- and 12-week-old mice of indicated genotypes. (**h**) Representative M-mode echocardiography recordings (upper) and HE-stained sections of the heart mid-portion (lower, Scale bar, 1 mm) 6 weeks after TAC or sham surgery. (**i**) Comparisons of indicated heart parameters between *Angptl2* KO and WT littermate mice 6 weeks after TAC or sham surgery. (**j**) Relative expression of genes associated with HF in heart 6 weeks after TAC or sham surgery. *n*=5–6 (**a**–**f** and **h**–**j**) and 9 (**g**) per group. (**k**) ATP levels in hearts of *Angptl2* KO and WT littermate mice 3 weeks after TAC or sham surgery. *n*=7–9 per group. Data are means±s.e.m. Statistical significance was determined by Student's *t* test (**a**–**g**,**j**,**k**, and percent reduction of FS and LVID;d in **i**) or two-way ANOVA (%FS in **i**). *P<0.05, **P<0.01, ^†^*P*<0.001 between genotypes or groups.

We next assessed *Angptl2* KO mice to determine whether cardiac ANGPTL2 deficiency alters pathological remodelling^17^. *Angptl2* KO mice are grossly normal at birth and show no overt cardiac phenotypes. Compared with WT controls, 6-week-old *Angptl2* KO mice showed increased cardiac systolic contractile ability but no cardiomyocyte enlargement, and 12-week-old *Angptl2* KO mice showed increased cardiac systolic contractile ability and exhibited cardiomyocyte enlargement with LV wall thickness (Fig. 5a–d). However, expression of HF markers in hearts of 12-week-old *Angptl2* KO mice was relatively decreased, while fibrosis markers remained unchanged (Fig. 5e). Expression of energy-related genes in hearts of 12-week-old *Angptl2* KO mice significantly increased, as did intracellular ATP production in both 6- and 12-week-old KO mice relative to WT littermates (Fig. 5f,g). *PGC-1α* and *PPARα* expression and that of *CD36* (a receptor for fatty acids) also significantly increased in 6-week-old *Angptl2* KO relative to WT control mice (Supplementary Fig. 6a). Consistently, *PPARα* and *PGC-1α* expression and intracellular ATP production significantly increased in *Angptl2-*KD primary NRCMs, while *PPARα* expression (but not *PGC-1α*) and ATP production significantly decreased in NRCMs transduced with adenovirus-expressing ANGPTL2 (Supplementary Fig. 7a–d). These findings indicate that ANGPTL2 suppression in cardiomyocytes increases expression of genes promoting β-fatty acid oxidation, mitochondrial biogenesis and intracellular ATP production.

To further assess *Angptl2* function we evaluated effects of TAC on *Angptl2* KO mice. At 6 weeks after TAC, WT littermate controls developed left ventricular dilatation with reduced fractional shortening, leading to HFrEF development (Fig. 5h,i). *Angptl2* KO mice also showed decreased fractional shortening, but to a lesser degree, and retained adaptive cardiac hypertrophy without ventricular dilatation (Fig. 5h,i; Supplementary Fig. 6b). HF markers expression was significantly lower in *Angptl2* KO compared with control mice (Fig. 5j), whereas fibrosis markers were comparably expressed (Supplementary Fig. 6c). Intracellular ATP production was maintained at a higher level after TAC in *Angptl2* KO relative to control mice, in agreement with its function in preserving cardiac contractility (Fig. 5k).

We next asked whether cardiac phenotypes observed in *Angptl2* KO mice were attributable to deficiency of cardiomyocyte-derived ANGPTL2. To do so, we generated cardiomyocyte-specific *Angptl2* conditional KO mice (*Angptl2*^*Flox/Flox*^*;αMHC-Cre*), and confirmed that ANGPTL2 protein levels in whole heart tissue of *Angptl2*^*Flox/Flox*^*;αMHC-Cre* mice decreased significantly relative to controls (Supplementary Fig. 8a–e). Six weeks after TAC, *Angptl2*^*Flox/Flox*^ control mice showed clear evidence of decreased fractional shortening and ventricular dilation, changes not seen in *Angptl2*^*Flox/Flox*^;*αMHC-Cre* mice (Supplementary Fig. 8f,g). Moreover, increased expression of HF markers was attenuated in *Angptl2*^*Flox/Flox*^;*αMHC-Cre* mice (Supplementary Fig. 8h), suggesting that loss of cardiomyocyte-derived ANGPTL2 is cardioprotective.

### ANGPTL2 inactivates AKT-SERCA2a signalling in heart

**Figure 6:**
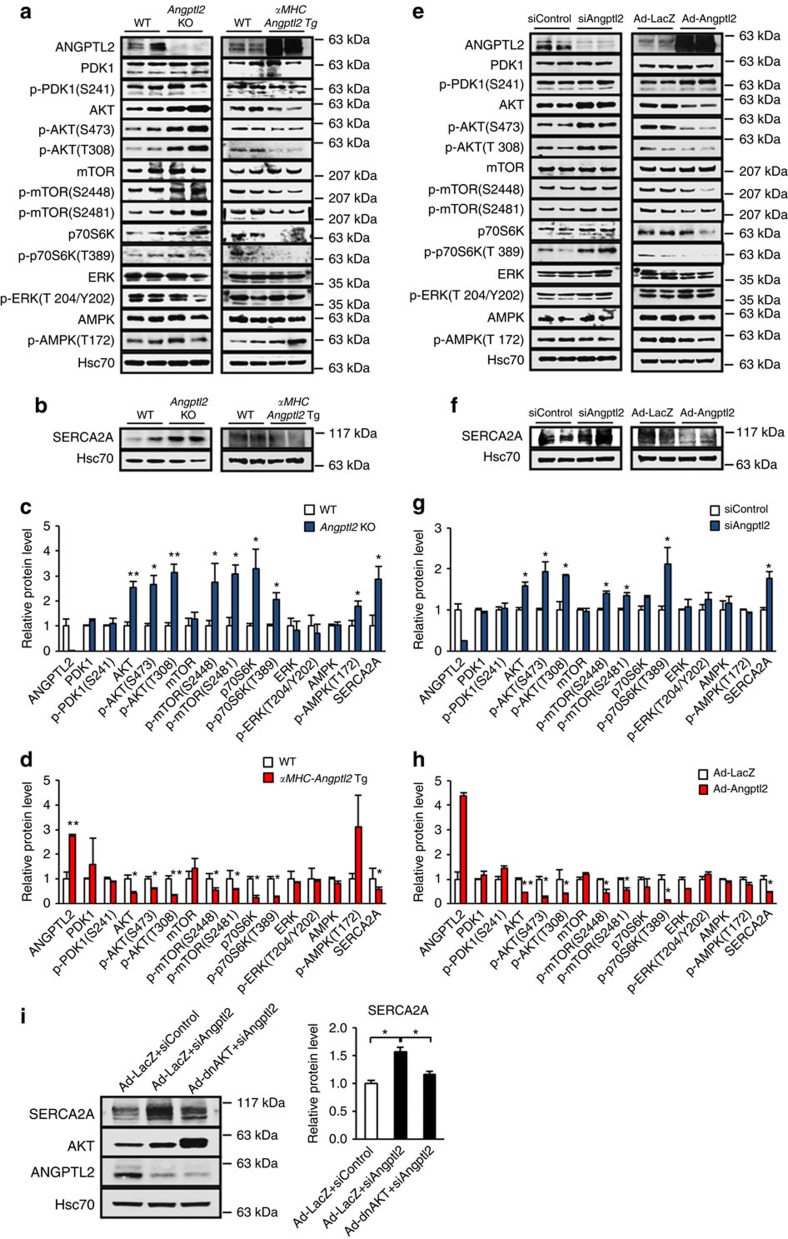
ANGPTL2 overexpression or deficiency alters AKT-SERCA2a signalling. (**a**–**d**) Representative western blotting and quantitation of ANGPTL2, various signalling proteins and SERCA2a in hearts of *Angptl2* KO (left), *αMHC-Angptl2* Tg (right) and respective littermate mice. Hsc70 served as a loading control. *n*=3–6 per group. (**e**–**h**) Representative western blots and quantitation of indicated factors in NRCMs transfected with ANGPTL2 or control siRNA (left) or infected with Ad-Angptl2 or control Ad-LacZ (right). *n*=3–5 per group. Experiments were performed at least two times. (**i**) Left, representative western blot showing SERCA2a, AKT and ANGPTL2 protein levels in NRCMs transduced with Ad-LacZ+ siRNA control, Ad-LacZ+ siAngptl2 or Ad-dnAKT (dominant-negative AKT)+siAngptl2 (*n*=3). Hsc70 served as a loading control. Right, quantification of SERCA2a protein levels in blot at left. Levels in Ad-LacZ+ siRNA control were set to 1. Data are means±s.e.m. Control values were set to 1. Statistical significance was determined by Student's *t* test (**c**,**d**,**g** and **h**) or one-way ANOVA (**i**). *P<0.05, **P<0.01 between genotypes or groups.

To identify mechanisms underlying the effect of ANGPTL2 on cardiac contractility, we employed a pathway scan comparing activation of signalling factors in hearts of *Angptl2* KO and WT control mice. That analysis showed that AKT and its effectors mTOR and p70S6K were slightly activated, but not in a statistically significant manner, in the heart of *Angptl2* KO mice (Supplementary Fig. 6d). We then performed western blotting analysis and observed activated AKT signalling and increased SERCA2a protein levels in hearts of *Angptl2* KO relative to control mice (Fig. 6a–c). Conversely, AKT signalling was attenuated and SERCA2a levels were significantly decreased in hearts of *αMHC-Angptl2* Tg mice (Fig. 6a,b,d). Moreover, AKT activation and higher SERCA2a protein levels were observed in *Angptl2*-KD NRCMs relative to controls (Fig. 6e–g), and increased SERCA2a levels induced by *Angptl2*-KD were significantly attenuated in *Angptl2*-KD NRCMs overexpressing a dominant-negative form of AKT (dnAKT) (Fig. 6i), suggesting that *Angptl2*-KD increases SERCA2a expression in an AKT-dependent manner. Conversely, AKT signalling was markedly inhibited and SERCA2a protein levels were comparably decreased in NRCMs overexpressing ANGPTL2 (Fig. 6e,f,h). Thus, activation of AKT-SERCA2a signalling following ANGPTL2 loss likely increases cardiac contractility, while ANGPTL2 overexpression promotes the opposite phenotype.

### ANGPTL2 perturbs the AKT-SERCA2a pathway via Integrin β1

Our *in vitro* analysis suggests that ANGPTL2 is secreted from cardiomyocytes, an idea confirmed by observation that its secretion significantly decreased in supernatants of NRCMs treated with *Angptl2* siRNA (Supplementary Fig. 7e). Moreover, AKT-SERCA2a signalling was significantly downregulated in NRCMs treated with recombinant human ANGPTL2 protein (Supplementary Fig. 7f), a phenomenon consistent with results seen in heart of *αMHC-Angptl2* Tg mice and in NRCMs overexpressing ad-ANGPTL2 (Fig. 6a,b,d–f,h).

We previously reported that integrin receptors play critical roles in transducing ANGPTL2 signals in several tissues, however these analyses did not include cardiomyocytes^17^,^21^. To investigate whether ANGPTL2 activity in cardiomyocytes requires integrin receptors, we prepared three siRNAs designed to target integrin β1 and tested their efficiency in NRCMs (Supplementary Fig. 7g). On the basis of that analysis we chose two siRNAs to test whether integrin KD would alter ANGPTL2 signalling in cardiomyocytes. As expected, NRCMs transfected control siRNA showed downregulated AKT-dependent SERCA2a protein levels in response to transfection with adenovirus-expressing ANGPTL2. By contrast we observed little or no effect of ANGPTL2 on AKT-dependent SERCA2a protein levels in cells transfected with either of the integrin β1 siRNAs (Supplementary Fig. 7h), suggesting that integrin β1 is required for ANGPTL2 activity in this context.

### Prevention of ANGPTL2 upregulation blocks HF development

**Figure 7:**
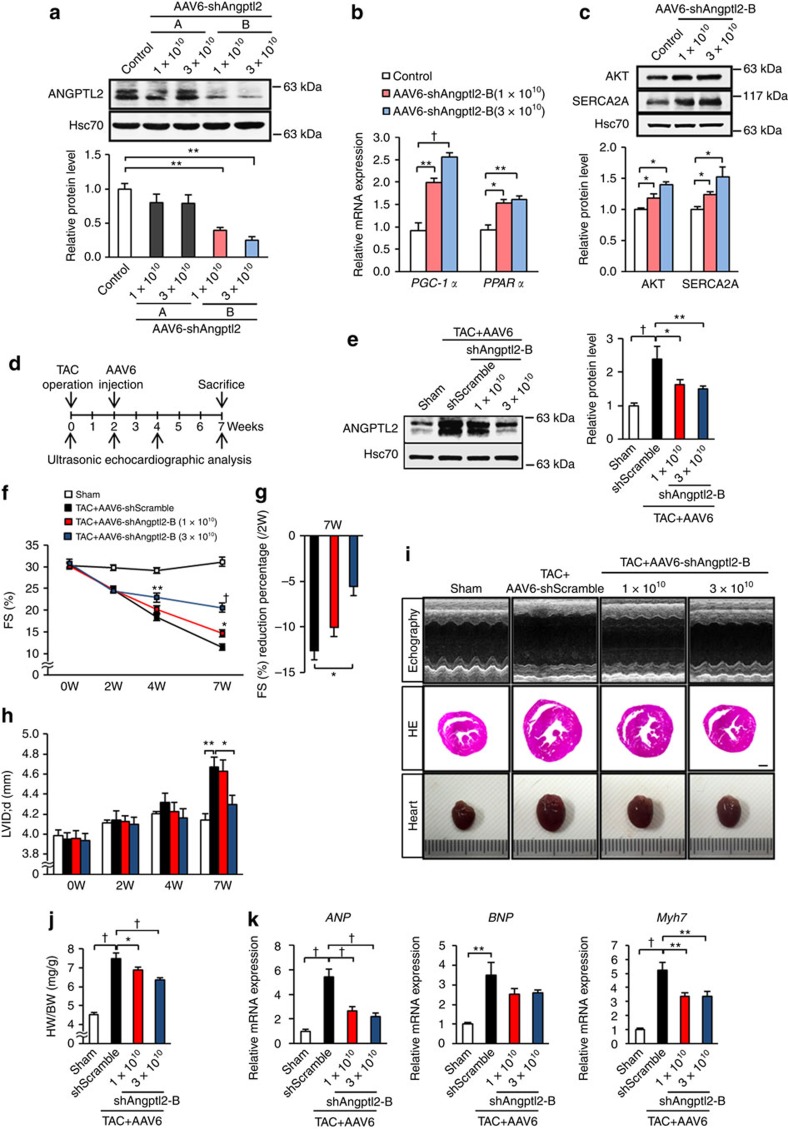
ANGPTL2 down-regulation in heart blocks progression to HF. (**a**–**c**) Representative immunoblot and quantitation of ANGPTL2 (**a**), relative levels of *PGC-1α* and *PPARα* transcripts (**b**) and levels of AKT and SERCA2a proteins (**c**) in hearts of WT mice 2 weeks after intravenous injection of 1 × 10^10^ or 3 × 10^10^ vg per mouse of AAV6-shAngptl2-A or -B relative to uninjected controls. Values from uninjected mice without were set to 1. *n*=4 per group. (**d**) Time line for AAV6-shAngptl2-B treatment and echocardiography analysis in the TAC model, 8–10 per group. (**e**) Representative western blot and quantitation of ANGPTL2 in TAC-induced hypertrophied heart, 4 weeks after injection of 3 × 10^10^ vg per mouse of AAV6-shScramble or 1 × 10^10^ or 3 × 10^10^ vg per mouse of AAV6-shAngptl2-B. (**f**–**h**) Comparison of indicated parameters among mice injected with 3 × 10^10^ vg per mouse of AAV6-shScramble or 1 × 10^10^ or 3 × 10^10^ vg per mouse of AAV6-shAngptl2-B. (**i**) Representative M-mode echocardiography recordings (upper), HE-stained sections of heart mid-portion (middle, Scale bar, 1 mm) and gross appearance of whole heart (bottom) in sham animals, and in TAC animals 4 weeks after injection of AAV6-shScramble or AAV6-shAngptl2-B (1 × 10^10^ or 3 × 10^10^ vg per mouse). Ruler tick marks indicate 1 mm. (**j**) HW/BW ratios of animals in **h**. (**k**) Relative expression of genes associated with HF. Control values from sham surgeries were set to 1 (**e**,**k**). Hsc70 served as a loading control. Data are means±s.e.m. Statistical significance was determined by one-way (**a**–**c**,**e**,**g**,**h**,**j**,**k**) or two-way (**f**) ANOVA. *P<0.05, **P<0.01, ^†^*P*<0.001.

To assess whether *in vivo* transfer of anti-ANGPTL2 reagents could counteract pathological cardiac remodelling, we delivered *Angptl2* shRNA cassettes into mouse heart by intravenous injection of recombinant adeno-associated virus serotype 6 (AAV6) vector harbouring *Angptl2* shRNA, as it has been shown that systemic delivery of recombinant AAV6 vectors preferentially transduces cardiac muscle^35^. We generated two constructs expressing mouse *Angptl2* shRNAs (AAV6-shAngptl2-A and -B), and intravenously injected 1 × 10^10^ vg and 3 × 10^10^ vg per mouse of each separately into WT mice. Two weeks later, ANGPTL2 expression in hearts of mice injected with AAV6-shAngptl2-B significantly decreased, whereas the AAV6-shAngptl2-A construct had no effect (Fig. 7a). Heart tissues from mice injected with AAV6-shAngptl2-B showed increased expression of *PGC-1α* and *PPARα* (Fig. 7b) and increased activation of AKT-SERCA2a signalling (Fig. 7c) relative to controls. Notably, we observed no adverse effects in terms of body weight, heart weight or expression of inflammatory markers in hearts of mice injected with AAV6-shAngptl2-B (Supplementary Fig. 9a–c), suggesting minimal cardiotoxicity.

Next, we asked whether intravenous injection of AAV6-shAngptl2-B suppressed ANGPTL2 upregulation *in vivo* in the TAC model, in which hypertrophy develops 2 weeks after surgery. To do so, we divided mice showing TAC-induced cardiac dysfunction at 2 weeks into three groups and confirmed that there was no difference in cardiac dysfunction, as evaluated by ultrasonic echocardiographic analysis among three groups (Fig. 7f,h; Supplementary Fig. 9d). We then intravenously injected mice with 1 or 3 × 10^10^ vg per mouse of recombinant AAV6-shAngptl2-B or an equivalent doses of Scramble control virus and examined cardiac function by ultrasonic echocardiography at various time points after injection (Fig. 7d). Both AAV6-shAngptl2-B doses suppressed increases in ANGPTL2 concentrations seen in the circulation of control animals (Supplementary Fig. 9e) as well as ANGPTL2 upregulation in stressed heart under pressure overload (Fig. 7e). Control mice showed left ventricular dilatation with reduced fractional shortening, whereas mice receiving either AAV6-shAngptl2-B dose showed attenuated cardiac dysfunction and a block in dilatation of the left ventricle, although ventricular wall thickness was comparable in all mice (Fig. 7f–i; Supplementary Fig. 9f). Heart weight per body weight ratios decreased in mice injected with either AAV6-shAngptl2-B dose relative to controls (Fig. 7j). Expression of HF markers significantly increased in hearts of control mice, while hearts of mice treated with either AAV6-shAngptl2-B dose did not show *ANP* or *Myh7* upregulation (Fig. 7k).

### ANGPTL2 suppression in human iPS-derived cardiomyocytes

**Figure 8:**
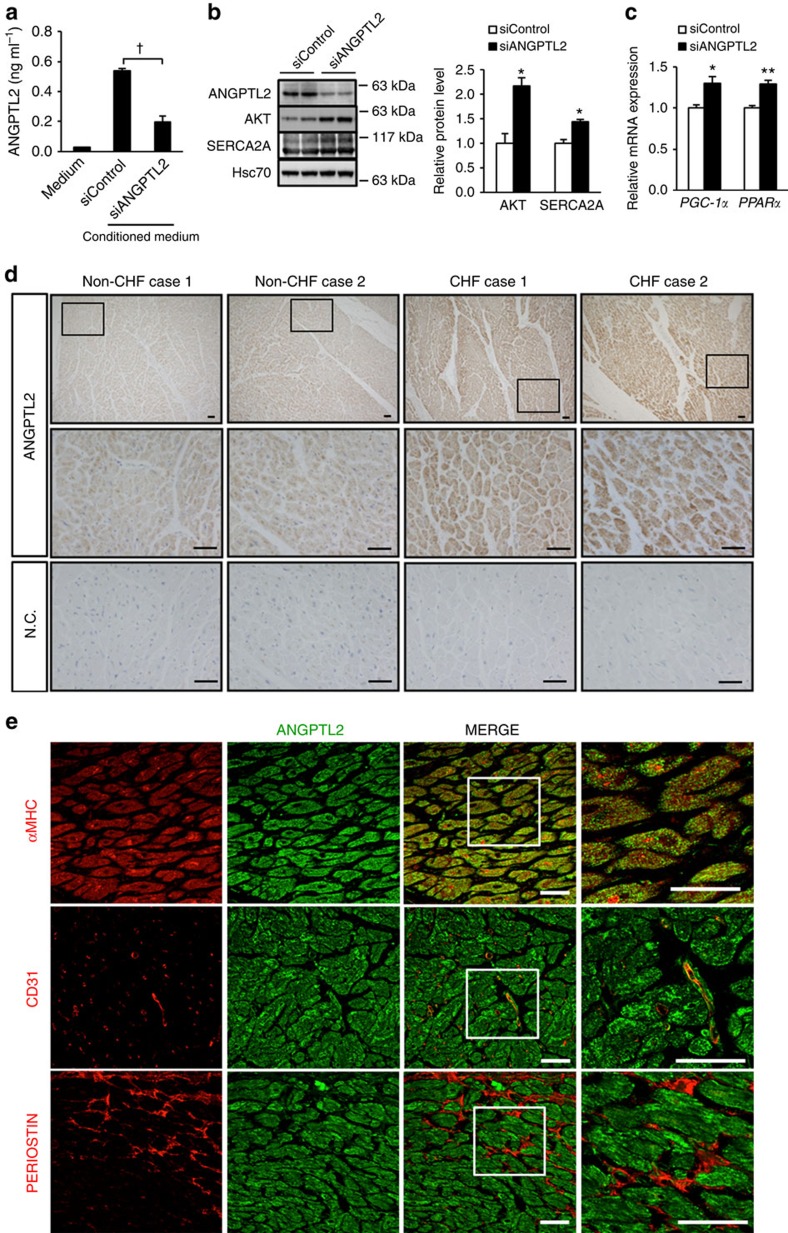
Effects of ANGPTL2*-*KD on human cardiomyocytes. (**a**) ANGPTL2 protein levels in conditioned medium of human iPS-derived cardiomyocytes transfected with siRNA targeting ANGPTL2 (siANGPTL2) or control siRNA (siControl) (*n*=4 per group). Maintenance medium for iPS-derived cardiomyocytes (medium) served as a negative control. (**b**) Representative western blot of ANGPTL2, AKT and SERCA2A proteins (left) and quantification of SERCA2A protein levels (right) in cardiomyocytes transfected with siANGPTL2 or control siRNA. Experiments were performed at least three times. Hsc70 served as a loading control. Levels in control siRNA cells were set to 1. (**c**) Relative expression of energy-related genes in cardiomyocytes transfected with siANGPTL2 or control siRNA (*n*=6 per group). Levels in control cells were set to 1. Data are means±s.e.m. Statistical significance was determined by Student's *t* test. *P<0.05, **P<0.01, ^†^*P*<0.001 between groups. (**d**) Immunohistochemical staining using antibody against ANGPTL2 or isotype control IgG (N.C.) in serial sections of heart tissues from patients with non-chronic HF (non-CHF) (Case 1: 77-year-old male with acute malignant lymphoma; Case 2: 82-year-old female with carcinomatous peritonitis) or with chronic HF (CHF) (Case 1: 63-year-old male; Case 2: 38-year-old female). Middle panels show magnified images of boxed regions in upper panels. Scale bars, 50 μm. (**e**) Immunofluorescent staining of *α*MHC (red; upper), CD31 (an endothelial cell marker) (red; middle), PERIOSTIN (a marker of cardiac fibroblasts) (red; lower) and ANGPTL2 (green) in heart tissues from a patient with CHF (Case 3: 85-year-old female). Magnified images of areas in squares are shown at right. Scale bars, 50 μm.

Next we asked whether ANGPTL2 suppression activated AKT-SERCA2a signalling and enhanced energy metabolism in human cardiomyocytes differentiated from iPS cells derived from human fibroblasts (see ‘Methods' section). That analysis revealed that human cardiomyocytes transfected with *ANGPTL2* siRNA showed decreased ANGPTL2 production and secretion, activated AKT-SERCA2a signalling and increased *PGC-1α* and *PPARα* expression relative to Scrambled siRNA controls (Fig. 8a–c).

### Upregulation of cardiac ANGPTL2 production in HF patients

Finally, we asked whether ANGPTL2 functions in pathological cardiac remodelling by evaluating ANGPTL2 protein levels in heart tissues obtained at autopsy from patients diagnosed with chronic HF (*n*=3) versus individuals who died in circumstances unrelated to heart disease (*n*=5). Immunohistochemistry with an anti-ANGPTL2 antibody revealed abundant ANGPTL2 protein in heart tissues from all three CHF patients (Fig. 8d for representative results from CHF cases 1 and 2). By contrast, all five control individuals showed cardiac ANGPTL2 protein levels lower than those seen in CHF patients (Fig. 8d for representative results from non-CHF cases 1 and 2). We next asked which cell types expressed Angptl2 in heart. Immunohistochemistry with an anti-ANGPTL2 antibody revealed abundant ANGPTL2 protein in αMHC-positive cardiomyocytes and CD31-positive capillary vessel endothelial cells but not in periostin-positive cardiac fibroblasts in tissues from all three CHF patients (Fig. 8e for representative results from CHF case 3).

The coronary sinus (Cs) drains blood primarily from the left ventricular chamber of the heart. Therefore, potential differences in ANGPTL2 concentration between the Cs and aortic root (Ao) would reflect ANGPTL2 secretion from heart tissues, including cardiomyocytes in the left ventricular chamber. To determine whether that type of expression pattern correlates with ventricular contractile dysfunction in patients, we compared ANGPTL2 concentration in the Cs and Ao of 58 patients with non-secondary and non-familial dilated cardiomyopathy (DCM) and observed significantly elevated (>35% increase) ANGPTL2 production in heart tissues of 23 (Supplementary Table 1). However, we observed no difference in severity of cardiac dysfunction between these 23 and the 35 patients that did not exhibit this pattern, as determined by several clinical indices (Supplementary Table 1). Patients with signs of increased cardiac ANGPTL2 secretion were generally older and exhibited mildly enlarged left ventricular end-diastolic dimension (LVDd) diameter compared with other patients (Supplementary Table 1).

## Discussion

The main findings of this study are: (1) cardiac ANGPTL2 production increases in stressed mouse heart as it undergoes pathological remodelling and potentially in some DCM patients; (2) pathological stimuli increase cardiomyocyte ANGPTL2 production via calcineurin-NFAT signalling; (3) ANGPTL2 protein secreted from cardiomyocytes predisposes heart to HF development likely by perturbing left ventricular contractile ability, an outcome attributable to inactivation of AKT-SERCA2a signalling and decreased myocardial energy metabolism in cardiomyocytes; (4) exercise training and/or miR-221/222 activity decreases cardiac ANGPTL2 expression; (5) *Angptl2* KO mice are protected from pathological cardiac remodelling, and their heart phenotypes resemble those induced by exercise; (6) AAV6-shAngptl2 blocks cardiac ANGPTL2 upregulation in heart stressed by pressure overload and blocks the transition to HF by activating AKT-SERCA2a signalling and improving energy metabolism in cardiomyocytes in mice; and (7) AKT-SERCA2a is activated and myocardial energy metabolism is enhanced in ANGPTL2-KD human iPS-derived cardiomyocytes. Overall, we showed that increased cardiac ANGPTL2 activity accelerates cardiac dysfunction and that therapeutic ANGPTL2 suppression could antagonize this dysfunction.

Others reported that circulating ANGPTL2 levels in HF patients were higher than in control individuals, and that cardiac function was inversely correlated with levels of ANGPTL2 circulating in the periphery^36^. The authors did not address underlying mechanisms, but noted that increased levels of circulating ANGPTL2 in these patients could be secondary to increased ANGPTL2 secretion from adipose tissues under pathological conditions of HF. Such an outcome is feasible given induction of adipose tissue inflammation and subsequent insulin resistance seen in HF conditions^37^,^38^ and the fact that circulating ANGPTL2 levels are relatively high in subjects with adipose tissue inflammation and/or insulin resistance^17^. However, our study strongly suggests that pathological stimuli activate ANGPTL2 production in cardiomyocytes, and that heart-derived ANGPTL2 is the likely source of increased ANGPTL2 levels in circulation in HF conditions. Conversely, suppression of cardiac ANGPTL2 production by treatment of AAV6-shAngptl2 decreased ANGPTL2 levels in circulation (Supplementary Fig. 9e). On the other hand, to date we and others have independently reported that circulating ANGPTL2 levels increase in various pathological conditions other than obesity accompanied by adipose tissue inflammation, such as atherosclerotic vascular disease and type 2 diabetes and that these levels are associated with disease severity^17^,^20^,^39^,^40^,^41^. Because, some of these conditions are risk factors for HF development, further studies are needed to investigate whether circulating ANGPTL2 affects cardiac function.

Non-pro-inflammatory ANGPTL2 function in cardiomyocytes reported in this study is noteworthy, as ANGPTL2 reportedly acts primarily by promoting progression of chronic inflammation^16^. However, we do not exclude the possibility that cardiomyocyte-derived ANGPTL2 may have a pro-inflammatory effect during pathological cardiac remodelling. Indeed, we have also reported that ANGPTL2 induces pro-inflammatory phenotypes in macrophages (Supplementary Fig. 10)^18^,^42^, suggesting that cardiomyocyte-derived ANGPTL2 modulates activity of cells other than cardiomyocytes, such as infiltrated macrophages, in a paracrine manner. If so, activities in those cells could promote heart tissue inflammation, worsening cardiac dysfunction^43^.

Interestingly, a recent paper demonstrated that cardiac miR-222 expression increased after endurance exercise training, an activity required for physiological cardiomyocyte growth in adult hearts, and that miR-222 expression protected against adverse cardiac remodelling^44^. miR-221 and miR-222 target the identical RNA sequence, and miR-221/222 overexpression in mice antagonized pressure overload-induced pathological cardiac remodelling^45^. However, it has been reported that pressure overload-induced cardiac miR-221 promotes HF development^46^,^47^. Thus, the role of cardiac miR-221/222 remains controversial. Here, we found that expression of both miR-221 and miR-222 markedly increased in pressure overload-induced cardiac stress following TAC (Supplementary Fig. 5b). Notably, we also found that cardiomyocyte-specific miR-221/222 KO mice were predisposed to HF development (Supplementary Fig. 5c–f), suggesting that miR-221/222 functions in cardioprotection. Accordingly, miR-221/222 KO mice showed significantly increased ANGPTL2 protein levels in stressed heart after TAC (Supplementary Fig. 5g), suggesting that ANGPTL2 is a direct target of miR-221/222 (ref. 42) (Fig. 4b,c) and that miR-221/222 promotes cardioprotection via ANGPTL2 suppression (Supplementary Fig. 5).

Myocardial hypoxia is a critical pathology in HF development^48^,^49^. Here, we also found that chronic hypoxia occurs in heart tissues of TAC animals (Supplementary Fig. 11). A recent paper reported that amelioration of aging-related capillary and blood supply deficits is associated with downregulated expression of vascular endothelial growth factor (VEGF), a key angiogenic factor, and its receptor (VEGFR) in heart^50^. That study also reported that cardiac VEGF and VEGFR mRNA levels increase following exercise training, increasing cardiac capillarization^50^. These findings indicate overall that VEGF signalling is critical for cardioprotective heart tissue remodelling through maintaining myocardial oxygen levels. Our study shows decreased ANGPTL2 production in mouse heart after endurance exercise training (Fig. 4a) and increased ANGPTL2 production in hearts of aged mice (Supplementary Fig. 12). Moreover, both anti-VEGF therapy^51^ and chronic hypoxia^23^ increase ANGPTL2 expression in tumour tissue. Since ANGPTL2, like VEGF, possesses pro-angiogenic activity^22^,^23^, increased ANGPTL2 expression in stressed hearts might be caused by hypoxia. However, our observations support the idea that increased ANGPTL2 in stressed hearts likely decreases cardiac contractility by perturbing AKT-SERCA2a signalling and myocardial energy metabolism rather than counteracting hypoxia by increasing angiogenesis. Thus, we hypothesize that cardiac ANGPTL2 production is partially regulated by myocardial oxygen levels, potentially down-regulating ANGPTL2 in physiological exercise-induced cardiac remodelling and up-regulating it in pathological myocardial hypoxia-associated remodelling.

Recent studies indicate that exercise-trained mice are protected from HF development^11^,^12^ and that activation of PI3K-AKT signalling is necessary for exercise-induced cardioprotection^28^. Furthermore, Tg mice overexpressing AKT in cardiomyocytes show cardiac hypertrophy with enhanced left ventricular function associated with increased SERCA2a expression^52^,^53^. We also observed cardioprotection following AKT-SERCA2a activation in *Angptl2* KO mice, while ANGPTL2 upregulation antagonized AKT activation. Our findings in cultured cardiomyocytes suggest that AKT is destabilized in the presence of ANGPTL2 (unpublished observations), although further investigation of this activity is required.

Interestingly, we observed ANGPTL2 production in heart of ∼40% of DCM patients tested here, suggesting that cardiac dysfunction may be worsened by ANGPTL2 activity and that this subset of patients might be candidates for therapeutic ANGPTL2 suppression. These findings also suggest that mechanisms underlying DCM vary among individuals and that personalized medicine approaches are required to treat these diseases. Moreover, we were surprised to find that left ventricular dilatation in cases of DCM harbouring some ANGPTL2-associated cardiac dysfunction was milder than that seen in DCM cases lacking an association with ANGPTL2. We also observed that DCM patients showing upregulated cardiac ANGPTL2 production were generally older than other DCM patients (Supplementary Table 1). These findings suggest that the impact of ANGPTL2 on progression of DCM may be less than that of other mechanisms, and that evaluating changes in cardiac ANGPTL2 production in DCM pathology might be useful to predict DCM progression, although further investigation is required. Moreover, it remains important to determine whether cardiac ANGPTL2 production contributes to cardiac dysfunction in HF patients without DCM. Interestingly, we also found that ANGPTL2 production in hearts of aged mice increased (Supplementary Fig. 12). Recently, abundant expression of ANGPTL2 was reported as a senescence-associated secretory phenotype factor in senescent cells^54^. Because pathologies are well documented in the aging heart, cardiac ANGPTL2 activation might be associated with dysfunction associated with aging.

ANGPTL2 reportedly interacts with the intracellular domain of the Ang II type1a (AT1A) receptor and promotes its recycling in non-cardiomyocyte cells^55^. In addition, we also recently reported down-regulation of ANGPTL2/Arap1 function in sepsis-induced hypotension due to reduced AT1A receptor recycling in vascular cells^56^. Ang II signalling through AT1A closely follows the progress of cardiac pathology, whereas suppression of ANGPTL2/Arap1 expression did not alter Ang II downstream signalling in cardiomyocytes (Supplementary Fig. 13), suggesting that ANGPTL2/Arap1 activity is not essential for AT1A receptor recycling in cardiomyocytes and that other AT1A receptor-associated proteins, such as ATRAP, might regulate recycling in this cellular context^57^.

**Figure 9:**
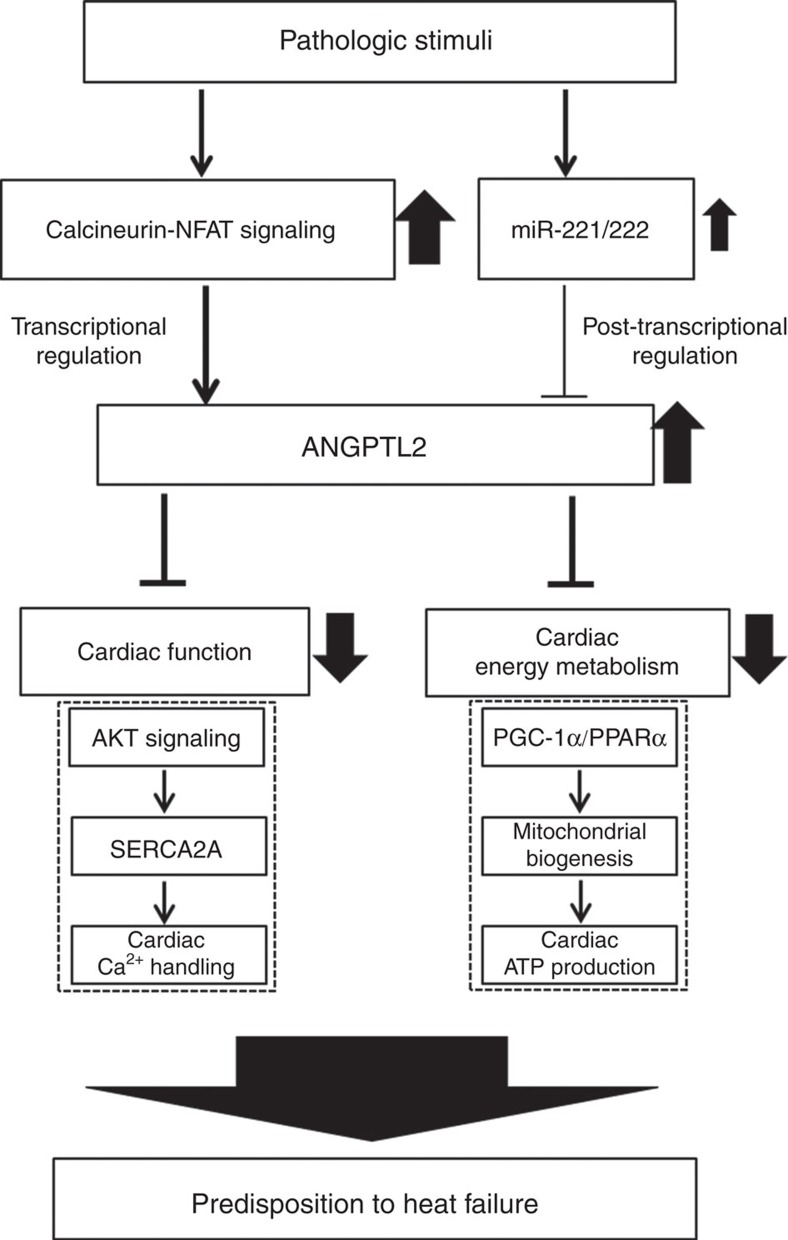
Proposed mechanism underlying ANGPTL2 activity in heart. Pathologic stimuli increase cardiomyocyte ANGPTL2 expression through Calcineurin-NFAT signalling. Cardiac ANGPTL2 activity causes pathological remodeling by down-regulating AKT-SERCA2A signalling, promoting cardiac dysfunction and decreasing cardiac energy metabolism via PGC-1α and PPAR-*α* down-regulation. Such pathological remodeling facilitates the transition to HF.

In summary, this is the first study to report that increased ANGPTL2 activity induced in pathologically stressed heart accelerates cardiac dysfunction (Fig. 9). ANGPTL2 suppression restored cardiac function and myocardial energy metabolism, blocking pathological remodelling.

## Methods

### Animal studies

All experimental procedures were approved by the Kumamoto University Ethics Review Committee for Animal Experimentation. All animals were fed a normal diet (ND; CE-2, CLEA, Tokyo, Japan), bred in a mouse house with automatically controlled lighting (12 h on, 12 h off), and maintained at a stable temperature of 23 °C. Genetically engineered mice used in the study were: *Angptl2* KO mice (*Angptl2* KO)^17^ on a C57BL/6NJcl background, Tg mice overexpressing EGFP driven by the murine *αMHC* promoter (*αMHC-EGFP)* on a C57BL/6J background, purchased from the Laboratory Animal Resource Bank, National Institutes of Biomedical Innovation, Health and Nutrition (Osaka, Japan), and Tg mice overexpressing *Cre* driven by the murine *αMHC* promoter (*αMHC-Cre*)^58^, kindly provided by Prof. Kinya Otsu (King's College London, United Kingdom), on a C57BL/6N background. *Angptl2* KO mice were maintained by heterozygous breeders. *Angptl2* KO (6-, 10- and 12-week-old), *αMHC-EGFP* (10-week-old), and *αMHC-Cre* (10-week-old) male mice were used in this study.

### Generation of αMHC-Angptl2 transgenic mice

cDNA encoding mouse *Angptl2* was cloned into the murine *αMHC* promoter expression vector in Supplementary Fig. 3a, kindly provided by Dr Jeffrey Robbins (Heart Institute of Cincinnati Children's Hospital Medical Center)^59^. To identify Tg offspring, genomic PCR was performed with forward (5′-ACTTCTACATGAGATCATTC-3′) and reverse (5′-GGTATTCTCAGGCTTCACCAGGTA-3′) primers. To maintain an isogenic strain, mice were propagated as heterozygotes by breeding with WT C57BL/6NJcl mice (CLEA). Animals from F2 or F3 generations were used in all studies.

### Generation of miR-221/222 conditional KO mice

*miR-221/222* conditional KO mice (*miR-221/222*^*Flox/y*^(male) and *miR-221/222*^*Flox/Flox*^(female)) on a C57BL/6N background were provided by the German Research Center for Environmental Health (GmbH; Neuherberg, Germany). To genotype offspring, genomic PCR of tail DNA was performed with forward (5′-GCTCTGTTTTCCTAAGTGATGG-3′) and reverse (5′-CTGACAGGAAGTAAATCATCTTAGC-3′) primers. To maintain an isogenic strain, mice were propagated as heterozygotes by breeding with WT C57BL/6NJcl mice (CLEA).

### Generation of *Angptl2* conditional KO mice

*Angptl2* conditional KO mice (*Angptl2*^*Flox/Flox*^) on a C57BL/6J background were generated by UNITECH (Chiba, Japan) as a custom order. In brief, to generate an *Angptl2*^*Flox/Flox*^ construct, genomic fragments from an *Angptl2* BAC clone (Roswell Park Cancer Institute) were cloned into the FRT-neo-FRT-loxP-DTA targeting vector, such that *Angptl2* Exon2 was flanked by loxP sites (Supplementary Fig. 8a). The targeting construct was linearized by *Sac*II and electroporated into C57BL/6J embryonic stem cells. Homologously recombined embryonic stem (ES) cell clones were identified by PCR and Southern blot. Chimeras were generated by injecting positive ES cells into Balb/c blastocysts and subsequently identified by coat colour. Chimeric male founder mice were mated to C57BL/6J female mice to generate F1 heterozygotes for the *Angptl2*^*Flox/Flox*^ line. Heterozygotes were crossed with FLP mice to delete the neomycin cassette flanked by FRT sites. To genotype offspring, genomic PCR of tail DNA was performed with forward (5′-ACACATGGAACAGAGTTACAGCTTC-3′) and reverse (5′-AACTTTTTCAGAGATTTAGCACAGG-3′) primers (Supplementary Fig. 8b).

### Establishment of hypertrophy mouse models

Male mice approximately 10-weeks-old (body weight of 23–25 g) were subjected to pressure overload using TAC surgery. In brief, mice were anaesthetized by intraperitoneal injection of pentobarbital, and anesthesia effects were assessed by a lack of response to toe pinching. Respiration was artificially controlled during surgery. The aortic arch was accessed via a left thoracotomy, and the thoracic aorta at the arch was surgically constricted using a 27-gauge needle to generate trans-stenotic pressure, as described elsewhere^49^. Sham mice underwent the same procedure without aortic banding.

### Echocardiography for mice

Mice were preconditioned by chest hair removal with a topical depilatory (FujiFilm VisualSonics, Toronto, Canada), anaesthetized with 1.5–2.5% isoflurane administered via inhalation, and maintained in a supine position on a dedicated animal handling platform with limbs attached for electrocardiogram gating during imaging. Body temperature was kept constant by feeding the signal of a rectal probe back to a heating pad, while heart and respiratory rates were continuously monitored. Transthoracic echocardiography was performed using a high frequency ultrasound system dedicated to small animal imaging (VisualSonics Vevo 2100, FujiFilm VisualSonics, Toronto, Canada) using a MS 400 linear array transducer (18–38 MHz). M-mode recording was performed at the midventricular level. All images were analyzed using dedicated software (Vevo 2100 version 1.4). LV wall thickness and internal cavity diameters at diastole (LVID;d) and systole (LVID;s) were measured. Per cent LV fractional shortening (%FS) was calculated from M-mode measurements. All procedures were performed under double-blind conditions with regard to genotype or treatment.

### Angiotensin II treatment

Angiotensin II (Ang II, Peptide Institute, Osaka, Japan) was dissolved in 150 mM NaCl and 1 mM acetic acid. Dorsal subcutaneous tissues of mice (male, 10-week-old) were implanted with a mini-osmotic pump (Model 2004, Alzet Corp., Palo Alto, CA, USA) to infuse Ang II (3 mg kg^−1^ per day) continuously for 2 weeks. Vehicle-treated groups underwent the same procedure with vehicle (150 mM NaCl and 1 mM acetic acid)-filled pumps.

### Isolation of cardiomyocytes and non-cardiomyocytes

Ventricles (three hearts per sample) were harvested from *αMHC-EGFP* transgenic mice (male, 10-week-old), and tissue was minced into small pieces and digested by 0.075% collagenase, 0.12% trypsin and 0.02% DNase at 37 °C for 40 min. Cells were collected, resuspended and then passed through a 100-μm mesh filter into 50-ml centrifuge tubes. Cells were finally resuspended with 0.5 ml FACS buffer (phosphate-buffered saline (PBS)/0.1% BSA) and GFP-positive (cardiomyocyte) and GFP-negative (non-cardiomyocyte) cells were isolated using a cell sorter FACSAria II (Becton Dickinson, San Jose, CA, USA).

To confirm that *Angptl2* is specifically deleted in cardiomyocytes of *Angptl2*^*Flox/Flox*^; *aMHC-Cre* mice, we isolated cardiomyocytes and non-cardiomyocytes from heart tissue of *Angptl2*^*Flox/Flox*^; *aMHC-Cre* and *Angptl2*^*Flox/Flox*^ mice (male, 10-week-old), as reported previously^60^. Briefly, ventricles were harvested from these mice and digested in 0.15% collagenase, 0.12% trypsin and 0.02% DNase I for 40 min. After digestion, cardiomyocytes and non-cardiomyocytes were separated by percoll density gradient centrifugation.

### Histological analysis

Mouse heart tissue samples were fixed in 4% paraformaldehyde for 24 h and embedded in paraffin. Blocks were cut into 4-μm-thick sections, air-dried and deparaffinized. Sections were stained with hematoxylin and eosin (H&E; Wako, Osaka, Japan) to evaluate morphology, or with wheat germ agglutinin (WGA) to evaluate cardiomyocyte size or Masson's Trichrome (Muto Pure Chemicals, Tokyo, Japan) to evaluate cardiac fibrosis. Quantification of cardiomyocyte size after Alexa Fluor 594-conjugated WGA (Life Technologies, Carlsbad, CA, USA) and DAPI (4′,6-diamidio-2-phenylindole, Life Technologies) staining was performed. In each ventricle, 100 cardiomyocytes were measured using BZ-H2A software (Keyence). Measurements were limited to cardiomyocytes cut perpendicular to their long axis at the level of a centred round cardiomyocyte nucleus. Quantification of fibrosis areas was undertaken by visualizing blue-stained areas. The fibrotic area was calculated using Image J software (National Institutes of Health) as the summation of blue-stained areas divided by total ventricular area. For cardiac hypoxia analysis, 3 or 6 weeks after TAC mice were sacrificed 1 hour after intraperitoneal injection of 10 mg ml^−1^ hypoxyprobe-1 (pimonidazole; Cosmo Bio, Tokyo, Japan) in saline, a dosage of 60 mg kg^−1^. Heart tissues were harvested and paraffin-embedded. Blocks were cut into 4-μm-thick sections, air-dried and deparaffinized. For immunostaining of pimonidazole adducts, specimens were incubated with FITC-conjugated IgG1 mouse monoclonal anti-hypoxyprobe-1 antibody (5 μg ml^−1^) at 4 °C overnight. After washing with PBS, secondary antibody reaction was performed using Histofine mouse stain kit (Nichirei Biosciences Inc) according to the manufacturers' instruction. Peroxidase activity was visualized by incubation with a 3,3-diaminobenzidine solution. Slides were mounted and examined using a BIOREVO BZ-9000 microscope (Keyence, Osaka, Japan).

### Real-time quantitative RT-PCR analysis

Total RNA was isolated using an RNeasy Mini Kit (Qiagen, Valencia, CA, USA). DNase-treated RNA was reverse transcribed using a PrimeScript RT reagent Kit (Takara Bio Inc, Shiga, Japan). Heart tissue was homogenized using a Multi-beads shocker (Yasui Kikai, Osaka, Japan). Real-time quantitative RT-PCR was performed using SYBER Premix Ex Taq II (Takara Bio Inc) and a Thermal Cycler Dice Real-Time system (Takara Bio). Relative transcript abundance was normalized to that of *18S* rRNA levels in mouse, rat and human samples. Primer sets used for RT-PCR are listed in Supplementary Tables 2–4.

### Measurement of cell shortenings and Ca^2+^ transients

After enzymatic isolation of ventricular cardiomyocytes, cell shortenings and Ca^2+^ transients in single cardiomyocytes were measured as described^61^. Ca^2+^ transients were recorded in cardiomyocytes loaded with 10 mM Indo-1 AM (Dojindo, Kumamoto, Japan). Briefly, isolated cardiomyocytes were stimulated in an electrical field at 1 Hz using a two-platinum electrode insert connected to a bipolar stimulator (SEN-3301, Nihon Kohden, Tokyo, Japan) on the stage of an inverted microscope (IX71, Olympus, Tokyo, Japan) with a 20 × water immersion objective lens (UApo N340, Olympus). Cell shortenings and Indo-1 fluorescent signals were recorded using a high-performance Evolve EMCCD camera (Photometrics, Tucson, AZ, USA) and analyzed by MetaMorph software (version 7.7.1.0; Molecular Devices, Sunnyvale, CA, USA). SR Ca^2+^ content was estimated by rapid extracellular application of 10 mM caffeine as described^61^. Caffeine-induced Ca^2+^ transients were recorded in cardiomyocytes loaded with 5 μM Fura-2 AM (Dojindo). The Fura-2 fluorescence ratio was determined using a Lambda DG-4 Ultra High Speed Wavelength Switcher (Sutter Instruments, Novato, CA, USA). The maximal amplitude of Ca^2+^ transients following caffeine application served as an index of SR Ca^2+^ content and was analyzed using MetaFluor software (Molecular Devices). Experiments were performed in cells superfused with Tyrode solution supplemented with 2 mg ml^−1^ BSA.

### Western blot analysis

Mouse heart tissue was homogenized in lysis buffer (10 mM Tris–HCl, 1% Triton X-100, 50 mM NaCl, 30 mM sodium pyrophosphate, 50 mM NaF, 5 mM EDTA, 0.1 mM Na_3_VO_4_, plus a protease inhibitor cocktail (Nacalai Tesque, Kyoto, Japan), pH 7.5) using a Multi-beads shocker (Yasui Kikai, Osaka Japan). Total protein (20 μg) or serum (0.1 μl) was separated by SDS–PAGE, and transferred to PVDF membranes. Membranes were incubated with anti-PDK1 (#3062), anti-p-PDK1 (S241) (#3438S), anti-AKT (#9272S), anti-p-AKT (s473), (#9271), anti-p-AKT (T308) (#4056), anti-mTOR (#2983), anti-p-mTOR(s2448) (#5536), anti-p-mTOR(s2481) (#2974), anti-p70S6k (#9202), anti-p-p70S6k(T389) (#9205S), anti-Erk (#9102S), anti-p-Erk(T204/y202) (#9106S), anti-AMPK (#2532S), anti-p-AMPK (T172) (#2535), anti-p-PKC(pan) (#9371s), anti-PKCα (#2056s; Cell Signaling Technology, Danvers, MA) or anti-Serca2a (ab3625; Abcam, Cambridge, MA, USA) antibodies diluted at 1:1,000 at 4 °C overnight. After washing with PBST, membranes were incubated with 1:2,000 diluted horseradish peroxidase (HRP)-conjugated sheep anti-rabbit IgG (GE Healthcare Life Science, Piscataway, NJ, USA) antibodies at room temperature for 60 min. For Angptl2 immunoblotting, membranes were incubated with 1:3,000 diluted biotinylated goat anti-Angptl2 antibody (BAF1444; R&D Systems, Minneapolis, MN, USA) at 4 °C overnight. After washing with PBST, membranes were incubated with 1:6,000 diluted HRP-conjugated streptavidin (Thermo Fisher Scientific Inc., Waltham, MA, USA) at room temperature for 60 min. Internal controls were 1:2,000 diluted mouse anti-Hsc70 (sc-7298; Santa Cruz Biotechnology, Santa Cruz, CA, USA) and 1:2,000 diluted HRP-conjugated sheep anti-mouse IgG (GE Healthcare Life Science) antibodies, which were used as primary and secondary antibodies, respectively. Blots were incubated with ECL Western Blotting Detection Reagent (GE Healthcare Life Science), visualized using a Luminescent Image Analyzer LAS-4000 system (Fujifilm, Tokyo, Japan) and quantified with Multi Gauge software (Fujifilm). Hsc70 was used for normalization. Uncropped scans of all immunoblots are shown in Supplementary Fig. 14.

### Measurement of intracellular ATP

ATP levels in heart tissues were determined using an ATP assay kit (Toyo Ink, Tokyo, Japan) according to the manufacturer's instructions. Briefly, heart tissue pieces (100 mg) were homogenized in 10 ml homogenate buffer (0.25 M sucrose in 10 mM HEPES-NaOH, pH 7.4) and centrifuged at 1,000*g* at 4 °C for 10 min. Supernatants were diluted eightfold on ice in homogenate buffer. Then, 100 μl of the mixture was added to 100 μl ATP extraction solution and luminescence was measured in Luminometer model TD-20/20 (Turner Designs, Promega, Japan).

ATP levels in NRCMs were determined using the same kit. Briefly, 48 hrs after cells were transduced, NRCMs (2 × 10^5^ cells) were washed with PBS twice and suspended in 100 μl of PBS. The cell suspension was inoculated into 96-well plates followed by the addition of 100 μl of ATP assay solution. After shaking for 1 min and incubation for 10 min at room temperature, luminescence was measured using a fluoroskan ascent microplate luminometer (Thermo Fisher Scientific Inc) and analyzed using Ascent software Version 2.6.

### NRCM culture

NRCMs were prepared as described previously^62^. In brief, ventricles of 1- to 2- day-old neonatal Wistar rats (Kyudo, Saga, Japan) were dissociated in 0.06% trypsin, 0.025% collagenase type II and 20 μg ml^−1^ of DNase I for 40 min. Cardiomyocytes were separately prepared by a percoll density gradient procedure. NRCMs were collected and seeded at 1 × 10^5^ cells per cm^2^ on Collagen (type I)-coated 24-well culture plates (Wako). Cells were grown in 199/DMEM medium (Life Technologies) supplemented with 10% fetal calf serum and antibiotics in a humidified atmosphere at 37 °C with 5% CO_2_. After 24 h, media was changed before treatment with various reagents or adenovirus infection unless otherwise specified.

ANGPTL2*-*KD in NRCMs were established by transfection with Mission siRNA Universal Negative Control (siControl; Sigma-Aldrich) or with three validated Mission siRNAs targeting *Angptl2* (siAngptl2-A: SASI_Rn01_00093802: GGAUCUUACUCACUCAAGATT, siAngptl2-B: SASI_Rn01_00093800: GAGAGUACAUUUACCUCAATT, siAngptl2-C: SASI_Rn01_00093799: CCAGAAAGCGAGUACUAUATT, Sigma-Aldrich) using Lipofectamine RNAiMAX reagent (Life Technologies) according to the manufacturer's instructions. At 48 h later, cells were treated with TRI Reagent (Cosmo Bio, Tokyo, Japan) in preparation for real-time RT-PCR or harvested and lysed with lysis buffer for immunoblot analyses.

For constitutively active NFAT overexpression in NRCMs, NRCMs were electroporated with pcDNA3.1 as a negative control or constitutively active mouse NFATc3 expression plasmid, kindly provided by Dr Takashi Minami (Institute of Resource Development and Analysis, Kumamoto University, Japan), using Amaxa rat cardiomyocyte-neonatal nucleofector Kit and Nucleofector Device (Lonza, Anaheim, CA, USA) according to the manufacturer's instructions. After electroporation, cells were plated and cultured for 24 h.

Production of recombinant adenovirus-expressing *Angptl2* (Ad-Angptl2) was conducted with Takara Bio. In brief, mouse *Angptl2* cDNA was cloned into the SmiI site of the pAxCAwtit2 cosmid vector (Takara Bio), which was used to transfect 293 cells. Recombinant adenoviruses expressing dominant-negative AKT (Ad-dnAKT) and LacZ (Ad-LacZ) were kindly provided by Dr Takashi Kadowaki (Department of Metabolic Diseases, The University of Tokyo, Japan)^63^. NRCMs were infected with adenovirus vectors 1 hr after seeding at a multiplicity of infection of 50. Culture medium was then replaced with new medium for 48 h.

For treatment with exogenous protein, NRCM cultures were incubated with 10 μg ml^−1^ recombinant human ANGPTL2 for 24 h (ref. 23).

For Ang II and Isoproterenol (Iso; Sigma-Aldrich) treatment, NRCMs were stimulated with 100 nM Ang II or 100 nM Iso for 6 and 12 h. For cyclosporine A (CsA; Wako) treatments, NRCMs were pretreated with 10 μM CsA for 30 min before treatment with Ang II and Iso. For xestospongin C (Xes C; R&D systems) treatments, NRCMs were pretreated with 1 μM Xes C for 30 min before treatment with Ang II.

For NFAT immunocytochemistry staining, NRCMs were plated on collagen-coated coverslips with or without 100 nM Ang II in serum-free medium. After 12 h, cells were rinsed with PBS, fixed with 4% paraformaldehyde in PBS for 5 min and permeabilized with 0.4% Triton X-100 for 15 min. Nonspecific binding was minimized by blocking with 3% normal goat serum in PBS. Cells were incubated with anti-NFATC1 (sc-7294; Santa Cruz Biotechnology) or NFATC4 polyclonal antibodies (ab62613; Abcam) at 1 μg ml^−1^, followed by incubation with Alexa Fluor 488-conjugated anti-rabbit antibodies (Life Technologies). Nuclei were counterstained with 4′,6′-diamidino-2-phenylindole (DAPI, Life Technologies, 1:200). Images were obtained using a fluorescence microscope (model BZ-9000; Keyence).

### RAW264.7 cell culture and recombinant ANGPTL2 treatment

Mouse macrophage cell line RAW264.7 (TIB-71, American Type Culture Collection) was cultured in RPMI-1640 (Wako) containing 10% FCS in 5% CO/95% air at 37 °C. RAW264.7 cells were maintained in RPMI-1640 containing 1% FCS for 12 h and then incubated for additional 6 h with or without 5 μg ml^−1^ recombinant mouse ANGPTL2 protein prepared as described previously^23^. After incubation, for real-time RT-PCR analyses, total RNA from the cells was isolated as described above. TaqMan probes for *Mcp-1, Tnf-α, Ifnγ* and *18S rRNA* were purchased from Applied Biosystems. Real-time RT-PCR was performed using a Light Cycler 480 (Roche Diagnostics).

### Endurance exercise training

Ten-week-old male C57BL/6NJcl mice (CLEA Japan) were allowed to adapt to the treadmill chamber (Model MK-690S/4M, Muromachi, Japan) for 30 min with unlimited movement. Mice were then subjected to warm-up treadmill running for 15 min (at 5 m min^−1^ for 5 min, 10 m min^−1^ for 5 min and 15 m min^−1^ for 5 min) before real endurance exercise training began. Mice began treadmill running at a 20 m min^−1^ for 60 min as an acute endurance exercise. For chronic endurance exercise, mice repeatedly performed a warm-up for 15 min and subsequent treadmill running at 20 m min^−1^ for 60 min 5 days a week for 3 weeks. Mice were sacrificed 3 h after the last running exercise and heart tissue analyzed.

To assess endurance capacity, *αMHC-Angptl2* Tg and WT littermate male mice were adapted to the treadmill chamber for 30 min with unlimited movement and then subjected to warm-up running for 15 min (at 5 m min^−1^ for 5 min, 10 m min^−1^ for 5 min and 15 m min^−1^ for 5 min) before training began. Mice then began treadmill running at 20 m min^−1^ for 60 min as an acute exercise. As a chronic endurance exercise, mice repeatedly performed the warm-up for 15 min and then performed treadmill running at 20 m min^−1^ for 60 min 5 days a week for 3 weeks. Exhaustion was defined as falling back onto the grid more than three times in 10 s or resting on the electric grid for more than 15 s.

### Luciferase reporter assays

To construct a reporter plasmid (FLuc-Angptl2-3′UTR), the 3′UTR of mouse *Angptl2* was amplified from genomic DNA by PCR and then cloned into an XbaI site downstream of the firefly luciferase (FLuc) gene in the pGL3-Promotor Vector (Promega). To delete the miR-221/222 binding site from the *Angptl2*-3′UTR, we designed the following primer set: 5′-CATTTCTCATGTTCTGTGTATATATAAAAGGGAGG-3′ and 5′-AGAACATGAGAAATGCTGAGGTAACAGGGCAG-3′. Deletion of the miR-221/222 binding site in the Angptl2-3′UTR reporter (Fluc-Angptl2-3'UTR-Δ 221/222) was performed using a PrimeSTAR mutagenesis basal kit (Takara Bio) according to the manufacturer's instructions miR-221, miR-222, miR-211, miR-204 or miR-135a overexpression vectors were constructed by inserting sequences including the full-length mature microRNA sequences into pBApo-CMV (Takara Bio). NRCMs were co-transfected with pcDNA3.1 as a negative control or plasmids encoding microRNA plus the phRL-TK vector (Promega), which encodes renilla luciferase (RLuc) and either the FLuc-Angptl2-3′UTR or FLuc-Angptl2-3′UTR-Δ221/222 using Lipofectamine 3000 reagent (Life Technologies). Luciferase activity was determined using a Dual Glo luciferase assay system (Promega) according to the manufacturer's instructions.

### Recombinant AAV treatment

Production and purification of recombinant AAV6 vectors were conducted with Takara Bio. In brief, for shRNA synthesis, single-stranded DNA oligos A and B harbouring mouse *Angptl2*-targeting siRNA and complementary strands were designed as follows: A-top 5′-CTAGAGAGAGTACATTTACCTCAATAGTGCTCCTGGTTGTTGAGGTAAATGTACTCTCTTTTTTA-3′ and A-bottom 5′-CTAGTAAAAAAGAGAGTACATTTACCTCAACAACCAGGAGCACTATTGAGGTAAATGTACTCTCT-3′; B-top 5′-CTAGAGCCAGAAAGCGAGTACTATATAGTGCTCCTGGTTGTATAGTACTCGCTTTCTGGCTTTTTTA-3′ and B-bottom 5′-CTAGTAAAAAAGCCAGAAAGCGAGTACTATACAACCAGGAGCACTATATAGTACTCGCTTTCTGGCT-3′; Scramble-top 5′-CTAGAGTCTTAATCGCGTATAAGGCTAGTGCTCCTGGTTGGCCTTATACGCGATTAAGACTTTTTTA-3′ and Scramble -bottom 5′-CTAGTAAAAAAGTCTTAATCGCGTATAAGGCCAACCAGGAGCACTAGCCTTATACGCGATTAAGACT-3′. Single-stranded oligos (shAngptl2-A, shAngptl2-B and shScramble) were annealed and cloned into the pAAV-2 × U6 vector (Takara Bio). Recombinant AAV6 vectors were produced with an AAVpro Helper Free System (Takara Bio), purified by cesium chloride density gradient centrifugation and dialyzed against PBS. The genome copy number was determined using an AAVpro Titration Kit (for Real-time PCR) Ver. 2 (Takara Bio).

For analysis of TAC animals, 10-week-old male C57BL/6NJcl mice (CLEA Japan) were subjected to TAC surgery and 2 weeks later anesthetized with 2% isoflurane and intravenously injected with recombinant AAV6 vectors at 1 × 10^10^ vg or 3 × 10^10^ vg. Cardiac function was examined using echocardiography before injection (at 2 weeks after operation) and at 2 and 5 weeks afterwards. After echocardiography at 5 weeks, mice were sacrificed and heart tissues subjected to histological, real-time RT-PCR and immunoblot analyses. In some experiments (Fig. 7a–c), 10-week-old male C57BL/6NJcl mice were intravenously injected with recombinant AAV6 vectors at 1 × 10^10^ vg or 3 × 10^10^ vg. Two weeks after injection, mice were sacrificed and heart tissues subjected to real-time RT-PCR and immunoblot analyses.

### ANGPTL2-KD in human iPS-derived cardiomyocytes

Human iPS cell lines 253G4 or 836B3 served as pluripotent cells. Cardiomyocyte differentiation of both cell lines was induced as reported^64^,^65^. Derived cardiomyocytes were transfected with Mission siRNA Universal Negative Control (Sigma-Aldrich) or human *ANGPTL2*-targeting siRNA (s23854: GAGAGUUCAUUUACCUAAATT, Life Technologies) with Lipofectamine RNAi MAX reagent (Life Technologies) according to the manufacturer's instructions. At 12 h after transfection, the medium was changed and cells were incubated for an additional 48 h. ANGPTL2 concentration in conditioned medium from transfected cells was estimated with an ANGPTL2 ELISA kit (IBL, Fujioka, Japan) according to the manufacturer's instructions. Cells were treated with TRI Reagent (Cosmo Bio) for real-time RT-PCR analyses or harvested and lysed with RIPA (50 mM Tris–HCl, 150 mM NaCl, 0.5% sodium deoxycholate, 0.1% SDS, 1% Nonidet P-40, 1 mM EDTA, Protease inhibitor (Roche Diagnostics), pH 7.5) buffer for immunoblot analyses.

### Human studies

A total of 58 consecutive patients with DCM (40 men and 18 women; mean age±s.e.m., 54.7±1.7 years, Supplementary Table 1) were enroled in the study. Twenty-six were classified as New York Heart Association (NYHA) class I, 27 were class II and 5 were class III. Individuals with an episode of acute HF within the previous 3 months or with renal dysfunction (estimated glomerular filtration rate (eGFR) of <30 ml min^−1^ 1.73 m^−2^) were excluded from the study. All subjects underwent coronary angiography to exclude coronary artery disease and endomyocardial biopsy to exclude myocarditis or specific muscle disease. DCM was defined as the presence of both a LVEF of <50% (as revealed by contrast left ventriculography) and a dilated LV cavity in the absence of coronary artery stenosis of >50%, valvular heart disease, arterial hypertension and secondary cardiac muscle disease attributable to any known systemic condition^66^. No patients had histories of acute viral myocarditis or familial DCM. There was also no evidence that immune triggers functioned in DCM development in any patient. Patients were in stable condition before their referral to Nagoya University Hospital for cardiac catheterization. Endomyocardial biopsy was performed to exclude myocarditis or specific muscle disease. Written informed consent was obtained from each patient before cardiac catheterization, and the study was approved by the Human Ethics Committee of the Nagoya University School of Medicine, Japan (protocol approval No. 359-7, Marc 16th/2015).

### Cardiac catheterization analysis

All patients underwent diagnostic right and left heart catheterization as previously described^67^. In brief, pulmonary arterial wedge pressure and cardiac output (CO) were measured with the use of a Swan–Ganz catheter inserted through the right internal jugular vein. Cardiac index (CI) was calculated as follows: CI=CO per body surface area (l min^−1^ m^−2^). Coronary angiography and left ventriculography via the right radial or femoral artery were also performed. A 6F fluid-filled pigtail catheter was positioned in the left ventricle for measurement of LV pressure. EF was assessed by left ventriculography using the area-length method. To examine transcardiac release of serum ANGPTL2, at the time of biventricular catheterization ANGPTL2 from the aortic root (Ao) and ANGPTL2 from the coronary sinus (CS) were collected simultaneously. Serum ANGPTL2 levels were determined with human ANGPTL2 ELISA kits (IBL).

### Ultrasonic echocardiographic analysis of human patients

Two-dimensional echocardiography was performed using a ViVid 7 system (ViVid 7, GE Healthcare, Milwaukee, WI, USA) as described^67^. We measured LV end-diastolic dimension (LVDd), LV end-systolic dimension (LVDs) and left arterial dimension (LAD). Percent fractional shortening (%FS) was calculated from LVDd and LVDs. The LV mass index (LVMI) was calculated from two-dimensional measurements according to a formula approved by the American Society of Echocardiology^68^.

### Immunohistological analysis

Human heart tissue samples were obtained from autopsy cases, including from congestive HF (CHF) patients and non-CHF patients. In all cases, written informed consent was obtained from relevant families. The study was also approved by the Ethics Committees of Kumamoto University. Human heart tissue samples were fixed in 4% paraformaldehyde for 24 h and embedded in paraffin. Blocks were cut into 4-μm-thick sections, air-dried and deparaffinized. For immunohistochemistry, sections were pretreated with periodic acid (Nichirei, Tokyo, Japan) to inhibit endogenous peroxidases. Subsequently, specimens were incubated overnight with rabbit polyclonal anti-human ANGPTL2 antibody (1 μg ml^−1^) produced by immunizing rabbits with a synthetic peptide corresponding to amino acids 383–400 (SFRLEPESEYYKLRLGRY) of human ANGPTL2 at 4 °C (ref. 23). After washing with PBS, specimens were incubated with 500-fold diluted goat anti-rabbit IgG conjugated with peroxidase (GE Healthcare Life Science) as second antibody at room temperature for 60 min, and specimens were then counterstained with hematoxylin. As negative controls, the same procedures were performed using isotype control IgG in place of the primary antibodies. Peroxidase activity was visualized by incubation with a 3,3-diaminobenzidine solution and analyzed by light microscopy (model BZ-9000; Keyence). For double immunofluorescent staining, a rabbit polyclonal anti-human ANGPTL2 antibody (1 μg ml^−1^) was used with goat polyclonal anti-human MYH6 (sc-168676; Santa Cruz Biotechnology, 1 μg ml^−1^), mouse monoclonal anti-human CD31 (JC70A; DKO, Glostrup, Denmark, 2 μg ml^−1^) and goat polyclonal anti-human PERIOSTIN (sc-49480; Santa Cruz Biotechnology, 2 μg ml^−1^). Alexa Fluor 488-conjugated anti-rabbit or Alexa Fluor 594-conjugated anti-goat/mouse antibody (Life Technologies, 10 μg ml^−1^) served as secondary antibody. After washing with PBS, fluorescent images were captured by confocal laser microscopy (LSM410, Zeiss, Jena, Germany).

### Statistical analysis

No statistical methods were used to determine sample size, but the sample sizes were similar to those of previous reports^49^. No exclusion/inclusion criteria were applied to the mice used in this study. Group allocation and outcome assessment were performed in a blinded manner. *In vitro* experiments were repeated at least three times. All values were reported as the mean±s.e.m. Data were assessed with two-group comparisons of variables by unpaired two-tailed *t* test, with multiple comparisons by one-way ANOVA or two-way ANOVA with Tukey's multiple comparisons test between each group. Mouse survival data was analyzed by the Kaplan–Meier log-rank test. Analysis of Kaplan–Meier and ANOVA data was performed using GraphPad Prism software (version 6.0, GraphPad Software). A value of *P*<0.05 was considered statistically significant.

### Data availability

All data generated or analyzed during this study are included in this published article and its Supplementary information files, or are available from the corresponding author on reasonable request.

## Additional Information

**How to cite this article:** Tian, Z. *et al*. ANGPTL2 Activity in cardiac pathologies accelerates heart failure by perturbing cardiac function and energy metabolism. *Nat. Commun.* **7,** 13016 doi: 10.1038/ncomms13016 (2016).

## Supplementary Material

Supplementary Information Supplementary Figures 1-14 and Supplementary Tables 1-4.

## References

[b1] Bui A. L., Horwich T. B. & Fonarow G. C. Epidemiology and risk profile of heart failure. Nat. Rev. Cardiol. 8, 30–41 (2011). 21060326 10.1038/nrcardio.2010.165 PMC3033496

[b2] Giordano F. J. Oxygen, oxidative stress, hypoxia, and heart failure. J. Clin. Invest. 115, 500–508 (2005). 15765131 10.1172/JCI200524408 PMC1052012

[b3] Bhatia R. S. . Outcome of heart failure with preserved ejection fraction in a population-based study. N. Engl. J. Med. 355, 260–269 (2006). 16855266 10.1056/NEJMoa051530

[b4] Kass D. A., Bronzwaer J. G. & Paulus W. J. What mechanisms underlie diastolic dysfunction in heart failure? Circ. Res. 94, 1533–1542 (2004). 15217918 10.1161/01.RES.0000129254.25507.d6

[b5] Ingwall J. S. & Weiss R. G. Is the failing heart energy starved? On using chemical energy to support cardiac function. Circ. Res. 95, 135–145 (2004). 15271865 10.1161/01.RES.0000137170.41939.d9

[b6] McKinsey T. A. & Olson E. N. Cardiac hypertrophy: sorting out the circuitry. Curr. Opin. Genet. Dev. 9, 267–274 (1999). 10377279 10.1016/s0959-437x(99)80040-9

[b7] Molkentin J. D. & Dorn G. W. 2nd. Cytoplasmic signaling pathways that regulate cardiac hypertrophy. Annu. Rev. Physiol. 63, 391–426 (2001). 11181961 10.1146/annurev.physiol.63.1.391

[b8] Chien K. R. Stress pathways and heart failure. Cell 98, 555–558 (1999). 10490095 10.1016/s0092-8674(00)80043-4

[b9] Feldman A. M., Weinberg E. O., Ray P. E. & Lorell B. H. Selective changes in cardiac gene expression during compensated hypertrophy and the transition to cardiac decompensation in rats with chronic aortic banding. Circ. Res. 73, 184–192 (1993). 8508529 10.1161/01.res.73.1.184

[b10] Mann N. & Rosenzweig A. Can exercise teach us how to treat heart disease? Circulation 126, 2625–2635 (2012). 23184282 10.1161/CIRCULATIONAHA.111.060376 PMC4000019

[b11] McMullen J. R. & Jennings G. L. Differences between pathological and physiological cardiac hypertrophy: novel therapeutic strategies to treat heart failure. Clin. Exp. Pharmacol. Physiol. 34, 255–262 (2007). 17324134 10.1111/j.1440-1681.2007.04585.x

[b12] Gielen S., Schuler G. & Adams V. Cardiovascular effects of exercise training: molecular mechanisms. Circulation 122, 1221–1238 (2010). 20855669 10.1161/CIRCULATIONAHA.110.939959

[b13] Santulli G. Angiopoietin-like proteins: a comprehensive look. Front. Endocrinol. (Lausanne) 5, 4 (2014). 24478758 10.3389/fendo.2014.00004 PMC3899539

[b14] Kim I. . Molecular cloning, expression, and characterization of angiopoietin-related protein. angiopoietin-related protein induces endothelial cell sprouting. J. Biol. Chem. 274, 26523–26528 (1999). 10473614 10.1074/jbc.274.37.26523

[b15] Kubota Y. . Cooperative interaction of Angiopoietin-like proteins 1 and 2 in zebrafish vascular development. Proc. Natl Acad. Sci. USA 102, 13502–13507 (2005). 16174743 10.1073/pnas.0501902102 PMC1224617

[b16] Kadomatsu T., Endo M., Miyata K. & Oike Y. Diverse roles of ANGPTL2 in physiology and pathophysiology. Trends Endocrinol. Metab. 25, 245–254 (2014). 24746520 10.1016/j.tem.2014.03.012

[b17] Tabata M. . Angiopoietin-like protein 2 promotes chronic adipose tissue inflammation and obesity-related systemic insulin resistance. Cell Metab. 10, 178–188 (2009). 19723494 10.1016/j.cmet.2009.08.003

[b18] Tazume H. . Macrophage-derived angiopoietin-like protein 2 accelerates development of abdominal aortic aneurysm. Arterioscler. Thromb. Vasc. Biol. 32, 1400–1409 (2012). 22556334 10.1161/ATVBAHA.112.247866

[b19] Tian Z. . Perivascular adipose tissue-secreted angiopoietin-like protein 2 (Angptl2) accelerates neointimal hyperplasia after endovascular injury. J. Mol. Cell Cardiol. 57, 1–12 (2013). 23333801 10.1016/j.yjmcc.2013.01.004

[b20] Horio E. . Role of endothelial cell-derived angptl2 in vascular inflammation leading to endothelial dysfunction and atherosclerosis progression. Arterioscler. Thromb. Vasc. Biol. 34, 790–800 (2014). 24526691 10.1161/ATVBAHA.113.303116

[b21] Aoi J. . Angiopoietin-like protein 2 is an important facilitator of inflammatory carcinogenesis and metastasis. Cancer Res. 71, 7502–7512 (2011). 22042794 10.1158/0008-5472.CAN-11-1758

[b22] Endo M. . Tumor cell-derived angiopoietin-like protein ANGPTL2 is a critical driver of metastasis. Cancer Res. 72, 1784–1794 (2012). 22345152 10.1158/0008-5472.CAN-11-3878

[b23] Odagiri H. . The secreted protein ANGPTL2 promotes metastasis of osteosarcoma cells through integrin alpha5beta1, p38 MAPK, and matrix metalloproteinases. Sci. Signal. 7, ra7 (2014). 24448647 10.1126/scisignal.2004612

[b24] Mori J. . Agonist-induced hypertrophy and diastolic dysfunction are associated with selective reduction in glucose oxidation: a metabolic contribution to heart failure with normal ejection fraction. Circ. Heart Fail. 5, 493–503 (2012). 22705769 10.1161/CIRCHEARTFAILURE.112.966705

[b25] Dai D. F. . Mitochondrial oxidative stress mediates angiotensin II-induced cardiac hypertrophy and Galphaq overexpression-induced heart failure. Circ. Res. 108, 837–846 (2011). 21311045 10.1161/CIRCRESAHA.110.232306 PMC3785241

[b26] Pedram A., Razandi M., Aitkenhead M. & Levin E. R. Estrogen inhibits cardiomyocyte hypertrophy in vitro. Antagonism of calcineurin-related hypertrophy through induction of MCIP1. J. Biol. Chem. 280, 26339–26348 (2005). 15899894 10.1074/jbc.M414409200 PMC1249515

[b27] Morimoto T. . Calcineurin-GATA4 pathway is involved in beta-adrenergic agonist-responsive endothelin-1 transcription in cardiac myocytes. J. Biol. Chem. 276, 34983–34989 (2001). 11435416 10.1074/jbc.M005498200

[b28] Molkentin J. D. Parsing good versus bad signaling pathways in the heart: role of calcineurin-nuclear factor of activated T-cells. Circ. Res. 113, 16–19 (2013). 23788503 10.1161/CIRCRESAHA.113.301667 PMC3836615

[b29] van Rooij E. . Requirement of nuclear factor of activated T-cells in calcineurin-mediated cardiomyocyte hypertrophy. J. Biol. Chem. 277, 48617–48626 (2002). 12226086 10.1074/jbc.M206532200

[b30] Morgan J. P., Erny R. E., Allen P. D., Grossman W. & Gwathmey J. K. Abnormal intracellular calcium handling, a major cause of systolic and diastolic dysfunction in ventricular myocardium from patients with heart failure. Circulation 81, III21–III32 (1990). 2153479

[b31] Stanley W. C. & Chandler M. P. Energy metabolism in the normal and failing heart: potential for therapeutic interventions. Heart Fail. Rev. 7, 115–130 (2002). 11988636 10.1023/a:1015320423577

[b32] Huss J. M. & Kelly D. P. Nuclear receptor signaling and cardiac energetics. Circ. Res. 95, 568–578 (2004). 15375023 10.1161/01.RES.0000141774.29937.e3

[b33] Lin J., Handschin C. & Spiegelman B. M. Metabolic control through the PGC-1 family of transcription coactivators. Cell Metab. 1, 361–370 (2005). 16054085 10.1016/j.cmet.2005.05.004

[b34] Barger P. M., Browning A. C., Garner A. N. & Kelly D. P. p38 mitogen-activated protein kinase activates peroxisome proliferator-activated receptor alpha: a potential role in the cardiac metabolic stress response. J. Biol. Chem. 276, 44495–44501 (2001). 11577087 10.1074/jbc.M105945200

[b35] Gregorevic P. . Systemic delivery of genes to striated muscles using adeno-associated viral vectors. Nat. Med. 10, 828–834 (2004). 15273747 10.1038/nm1085 PMC1365046

[b36] Huang C. L., Wu Y. W., Wu C. C., Hwang J. J. & Yang W. S. Serum angiopoietin-like protein 2 concentrations are independently associated with heart failure. PLoS ONE 10, e0138678 (2015). 26397985 10.1371/journal.pone.0138678 PMC4580406

[b37] Shimizu I. . p53-induced adipose tissue inflammation is critically involved in the development of insulin resistance in heart failure. Cell Metab. 15, 51–64 (2012). 22225876 10.1016/j.cmet.2011.12.006

[b38] Ashrafian H., Frenneaux M. P. & Opie L. H. Metabolic mechanisms in heart failure. Circulation 116, 434–448 (2007). 17646594 10.1161/CIRCULATIONAHA.107.702795

[b39] Hata J. . Serum angiopoietin-like protein 2 is a novel risk factor for cardiovascular disease in the community: the Hisayama study. Arterioscler. Thromb. Vasc. Biol. 36, 1686–1691 (2016). 27365403 10.1161/ATVBAHA.116.307291

[b40] Doi Y. . Angiopoietin-like protein 2 and risk of type 2 diabetes in a general Japanese population: the Hisayama study. Diabetes Care 36, 98–100 (2013). 22966088 10.2337/dc12-0166 PMC3526200

[b41] Endo M. . Serum ANGPTL2 levels reflect clinical features of breast cancer patients: implications for the pathogenesis of breast cancer metastasis. Int. J. Biol. Markers 29, e239–e245 (2014). 24585434 10.5301/jbm.5000080

[b42] Morinaga J. . Angiopoietin-like protein 2 increases renal fibrosis by accelerating transforming growth factor-beta signaling in chronic kidney disease. Kidney Int. 89, 327–341 (2016). 26806834 10.1016/j.kint.2015.12.021

[b43] Anker S. D. & von Haehling S. Inflammatory mediators in chronic heart failure: an overview. Heart 90, 464–470 (2004). 15020532 10.1136/hrt.2002.007005 PMC1768165

[b44] Liu X. . miR-222 is necessary for exercise-induced cardiac growth and protects against pathological cardiac remodeling. Cell Metab. 21, 584–595 (2015). 25863248 10.1016/j.cmet.2015.02.014 PMC4393846

[b45] Peters T. . The microRNA-221/222 family is differentially regulated in cardiac disease and counteracts pressure overload-induced cardiac remodeling in mice. Cardiovasc. Res. Suppl. 103, S9–S46 (2014).

[b46] Wang C. . MiR-221 promotes cardiac hypertrophy in vitro through the modulation of p27 expression. J. Cell Biochem. 113, 2040–2046 (2012). 22275134 10.1002/jcb.24075

[b47] Su M. . MicroRNA-221 inhibits autophagy and promotes heart failure by modulating the p27/CDK2/mTOR axis. Cell Death Differ. 22, 986–999 (2015). 25394488 10.1038/cdd.2014.187 PMC4423182

[b48] De Boer R. A., Pinto Y. M. & Van Veldhuisen D. J. The imbalance between oxygen demand and supply as a potential mechanism in the pathophysiology of heart failure: the role of microvascular growth and abnormalities. Microcirculation 10, 113–126 (2003). 12700580 10.1038/sj.mn.7800188

[b49] Sano M. . p53-induced inhibition of Hif-1 causes cardiac dysfunction during pressure overload. Nature 446, 444–448 (2007). 17334357 10.1038/nature05602

[b50] Iemitsu M., Maeda S., Jesmin S., Otsuki T. & Miyauchi T. Exercise training improves aging-induced downregulation of VEGF angiogenic signaling cascade in hearts. Am. J. Physiol. Heart Circ. Physiol. 291, H1290–H1298 (2006). 16617130 10.1152/ajpheart.00820.2005

[b51] Crawford Y. . PDGF-C mediates the angiogenic and tumorigenic properties of fibroblasts associated with tumors refractory to anti-VEGF treatment. Cancer Cell 15, 21–34 (2009). 19111878 10.1016/j.ccr.2008.12.004

[b52] Shiojima I. & Walsh K. Regulation of cardiac growth and coronary angiogenesis by the Akt/PKB signaling pathway. Genes Dev. 20, 3347–3365 (2006). 17182864 10.1101/gad.1492806

[b53] Kim Y. K. . Mechanism of enhanced cardiac function in mice with hypertrophy induced by overexpressed Akt. J. Biol. Chem. 278, 47622–47628 (2003). 13129932 10.1074/jbc.M305909200

[b54] Shimamoto A. . Reprogramming suppresses premature senescence phenotypes of Werner syndrome cells and maintains chromosomal stability over long-term culture. PLoS ONE 9, e112900 (2014). 25390333 10.1371/journal.pone.0112900 PMC4229309

[b55] Guo D. F., Chenier I., Tardif V., Orlov S. N. & Inagami T. Type 1 angiotensin II receptor-associated protein ARAP1 binds and recycles the receptor to the plasma membrane. Biochem. Biophys. Res. Commun. 310, 1254–1265 (2003). 14559250 10.1016/j.bbrc.2003.09.154

[b56] Mederle K. . The angiotensin II AT1 receptor-associated protein Arap1 is involved in sepsis-induced hypotension. Crit. Care 17, R130 (2013). 23844607 10.1186/cc12809 PMC4056110

[b57] Tanaka Y. . The novel angiotensin II type 1 receptor (AT1R)-associated protein ATRAP downregulates AT1R and ameliorates cardiomyocyte hypertrophy. FEBS Lett. 579, 1579–1586 (2005). 15757644 10.1016/j.febslet.2005.01.068

[b58] Oka T. . Cardiac-specific deletion of Gata4 reveals its requirement for hypertrophy, compensation, and myocyte viability. Circ. Res. 98, 837–845 (2006). 16514068 10.1161/01.RES.0000215985.18538.c4

[b59] Gulick J., Subramaniam A., Neumann J. & Robbins J. Isolation and characterization of the mouse cardiac myosin heavy chain genes. J. Biol. Chem. 266, 9180–9185 (1991). 2026617

[b60] Zhou Y. Y. . Culture and adenoviral infection of adult mouse cardiac myocytes: methods for cellular genetic physiology. Am. J. Physiol. Heart Circ. Physiol. 279, H429–H436 (2000). 10899083 10.1152/ajpheart.2000.279.1.H429

[b61] Ujihara Y. . Induced NCX1 overexpression attenuates pressure overload-induced pathological cardiac remodelling. Cardiovasc. Res. 111, 348–361 (2016). 27229460 10.1093/cvr/cvw113

[b62] Yoshikawa N. . Ligand-based gene expression profiling reveals novel roles of glucocorticoid receptor in cardiac metabolism. Am. J. Physiol. Endocrinol. Metab. 296, E1363–E1373 (2009). 19293335 10.1152/ajpendo.90767.2008

[b63] Kamon J. . A novel IKKbeta inhibitor stimulates adiponectin levels and ameliorates obesity-linked insulin resistance. Biochem. Biophys. Res. Commun. 323, 242–248 (2004). 15351728 10.1016/j.bbrc.2004.08.083

[b64] Tohyama S. . Glutamine oxidation is indispensable for survival of human pluripotent stem cells. Cell Metab. 23, 663–674 (2016). 27050306 10.1016/j.cmet.2016.03.001

[b65] Uosaki H. . Efficient and scalable purification of cardiomyocytes from human embryonic and induced pluripotent stem cells by VCAM1 surface expression. PLoS ONE 6, e23657 (2011). 21876760 10.1371/journal.pone.0023657 PMC3158088

[b66] Oakley C. M. Report of the WHO/ISFC task force on the definition and classification of cardiomyopathies. Br. Heart J. 44, 672–673 (1980). 7459150 10.1136/hrt.44.6.672 PMC482464

[b67] Okamoto R. . Usefulness of serum cardiac troponins T and I to predict cardiac molecular changes and cardiac damage in patients with hypertrophic cardiomyopathy. Int. Heart J. 54, 202–206 (2013). 23924931 10.1536/ihj.54.202

[b68] Cheitlin M. D. . ACC/AHA/ASE 2003 guideline update for the clinical application of echocardiography: summary article: a report of the American College of Cardiology/American Heart Association Task Force on Practice Guidelines (ACC/AHA/ASE Committee to Update the 1997 Guidelines for the Clinical Application of Echocardiography). Circulation 108, 1146–1162 (2003). 12952829 10.1161/01.CIR.0000073597.57414.A9

