# Supplementary material for: ANGPTL2 activity in cardiac pathologies accelerates heart failure by perturbing cardiac function and energy metabolism
Source: Nat Commun. 2016 Sep 28;7:13016. doi: 10.1038/ncomms13016 (PMC5052800; doi:10.1038/ncomms13016)
Supplement: Supplementary Information — Supplementary Figures 1-14 and Supplementary Tables 1-4. [file ncomms13016-s1.pdf]

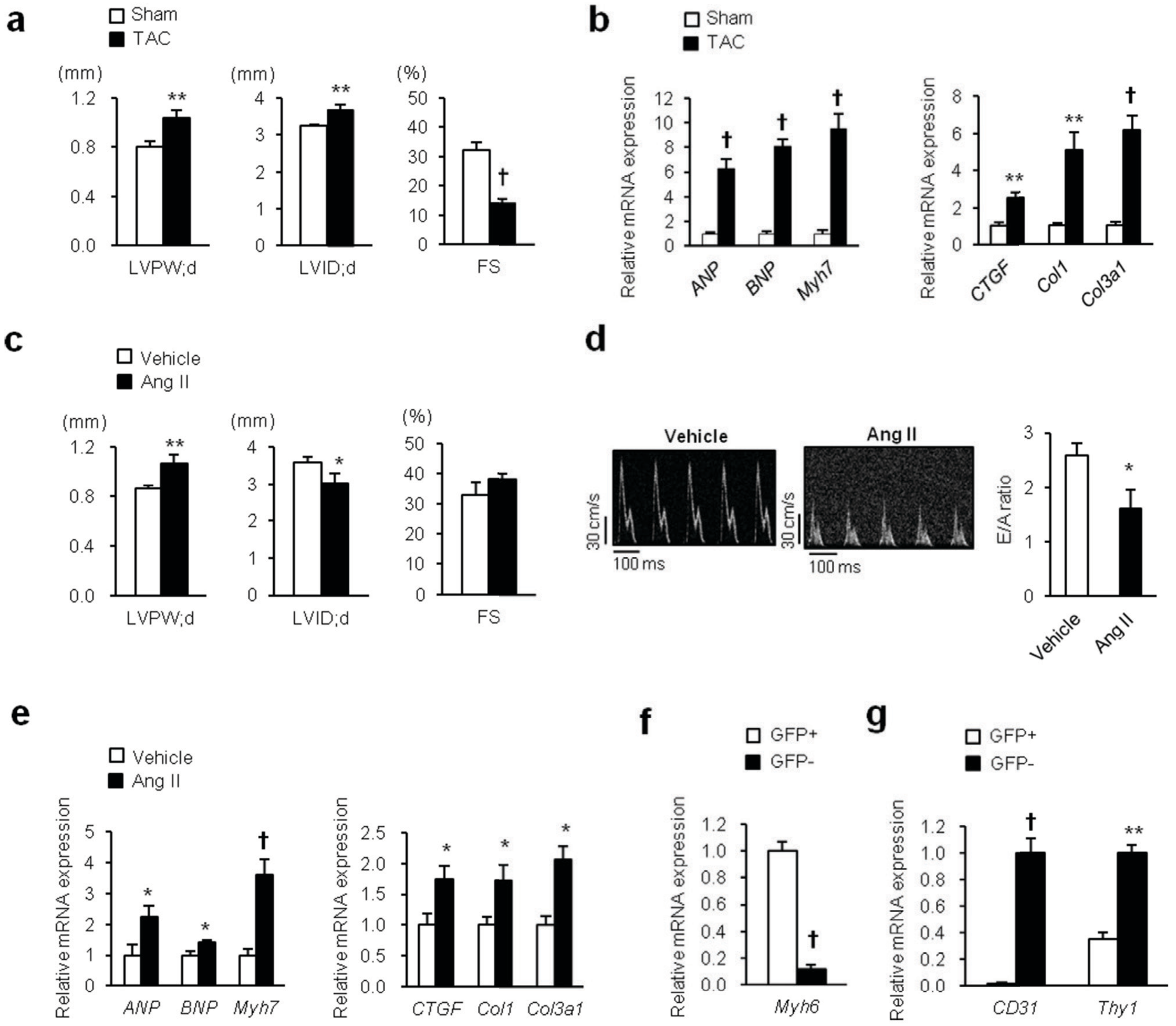

Supplementary Figure 1. Evaluation of cardiac function in mouse hypertrophy models.

(**a**) Diastolic left ventricular posterior wall thickness (LVPW;d) (left), left ventricular end diastolic internal diameter (LVID;d) (middle), and percent fractional shortening (% FS) (right) of WT mice 6 weeks after TAC or sham surgery (n = 5–7 per each group). (**b**) Relative expression of genes associated with heart failure (*ANP*, *BNP*, and *Myh7*) and cardiac fibrosis (*CTGF*, *Col1*, and *Col3a1*) in hearts of WT mice 6 weeks after TAC or sham surgery (n = 5–7 per each group). Values in sham-operated mice were set to 1. (**c**) LVPW;d, LVID;d, and % FS of WT mice 2 weeks after Angiotensin II (Ang II) or vehicle treatment (n = 8 per each group). (**d**) Representative transmitral doppler flow patterns (left) and the peak early distolic filing velocity (E-wave) per the peak atrial filing velocity (A-wave) ratio (E/A ratio) (right) of Ang II- or vehicle-treated WT mice (n = 7 per each group). (**e**) Relative expression of genes associated with heart failure and cardiac fibrosis in hearts of WT mice 2 weeks after Ang II or vehicle treatment (n = 8 per each group). Levels in vehicle-treated mice were set to 1. (**f**) Relative expression of mRNAs encoding the cardiomyocyte marker *Myh6* in GFP+ and GFP- cells isolated from heart of *Myh6-EGFP* Tg mice (n = 3). Levels in GFP+ cells were set to 1. (**g**) Relative expression of mRNAs encoding the non-cardiomyocyte markers *CD31* and *Thy1* in GFP+ and GFP- cells isolated from heart of *Myh6-EGFP* Tg mice (n = 3). Levels in GFP- cells were set to 1. Data are means ± SEM. Statistical significance was determined by Student's *t*-test. $*p < 0.05$, $**p < 0.01$, $^{\dagger}p < 0.001$ between groups.

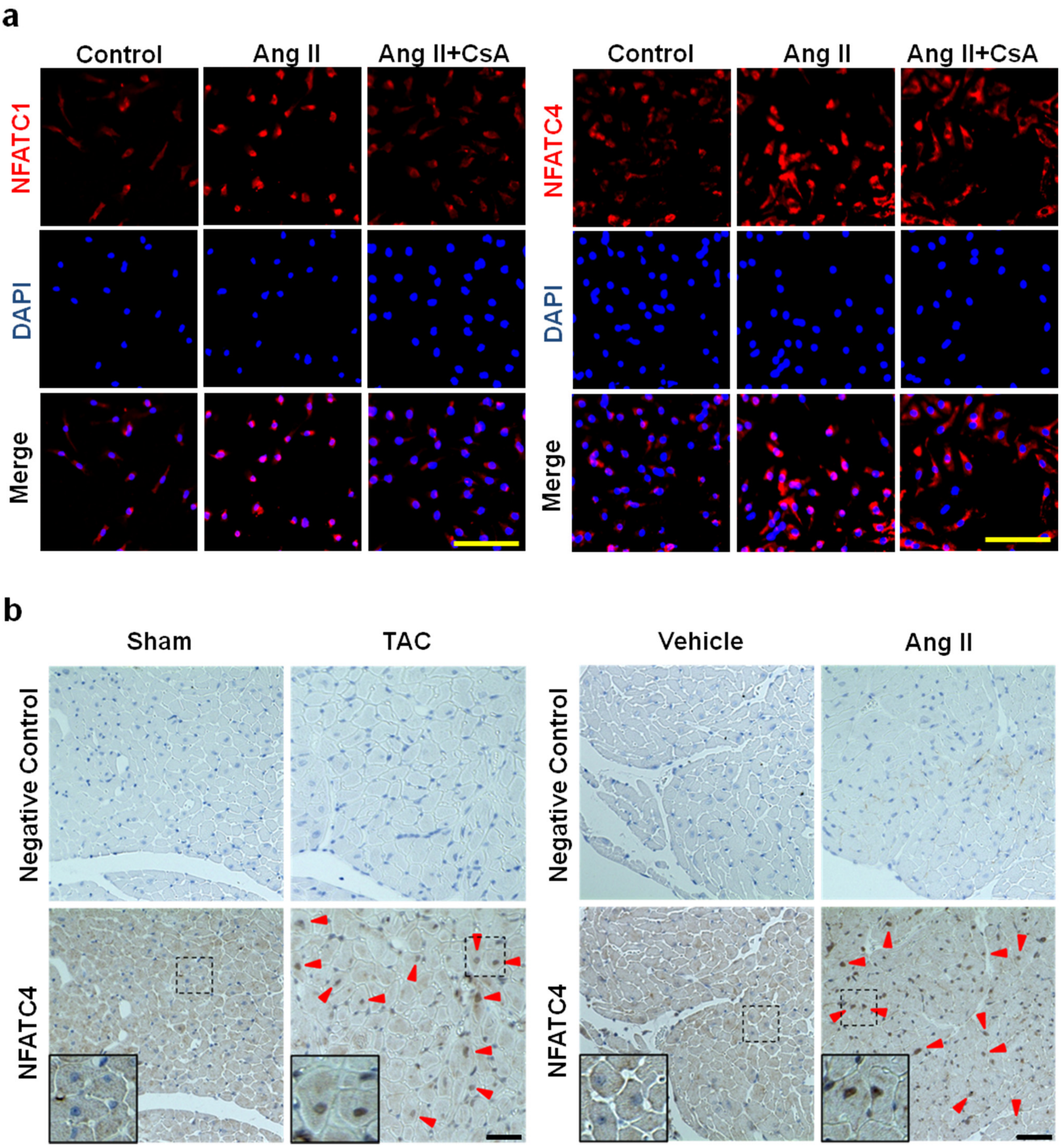

Supplementary Figure 2. TAC- and Ang II-induced nuclear translocation of NFAT in cardiomyocytes. (**a**) Immunofluorescent staining of NFATC1 (left) and NFATC4 (right) in NRCMs 12 h after Ang II treatment in the absence or presence of Cyclosporine A (CsA). Nuclei are counterstained with DAPI. Scale bars: 100 μm. (**b**) Immunohistochemical staining of NFATC4 (lower row) in mouse heart tissues 6 weeks after TAC or sham surgery (left) and 2 weeks after Ang II or vehicle administration (right). Negative controls (upper row) show the same location on the slide with no second antibody. Scale bars: 50 μm.

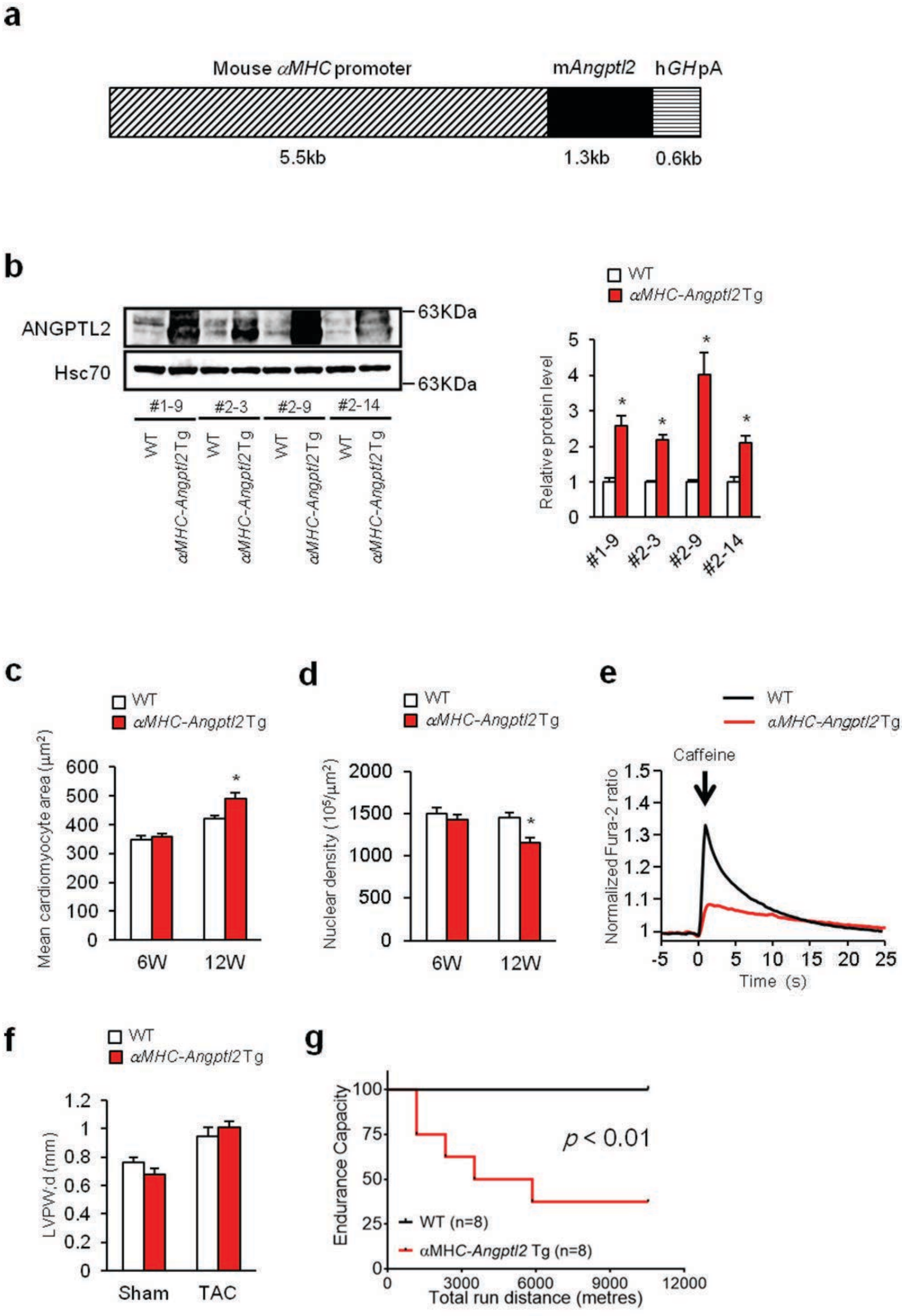

Supplementary Figure 3. Establishment of *αMHC-Angptl2* transgenic mice.
(**a**) Schematic showing the transgene used to generate *αMHC-Angptl2* transgenic mice (*αMHC-Angptl2* Tg). h*GH* pA, human growth hormone poly A. (**b**) Representative western blot (left) and quantification (right) of ANGPTL2 abundance in heart of 4 independent Tg and littermate WT lines (n = 4 per each group). Hsc70 was used as a

loading control. Levels in WT mice were set to 1. (**c,d**) Quantitation of area of cross-sections of cardiomyocytes shown in Figure 2A (middle and lower panels) (**c**) and nuclear density (**d**) in 8- and 12-week-old *αMHC-Angptl2* Tg and littermate WT mice (n = 5–6 per each group). (**e**) Representative caffeine-induced Ca^{2+} transients recorded in cardiomyocytes of *αMHC-Angptl2* Tg and littermate WT mice. (**f**) LVPW;d of *αMHC-Angptl2* Tg and littermate WT mice at 3 weeks after TAC or sham operation. (**g**) Endurance capacity as analyzed by treadmill training of 8-month-old *αMHC-Angptl2* Tg and WT mice (n = 8 per group). $p < 0.01$ between genotypes by log-rank test. Data are means ± SEM. Statistical significance was determined by Student's *t*-test (**b**–**e**). *$p < 0.05$ between genotypes.

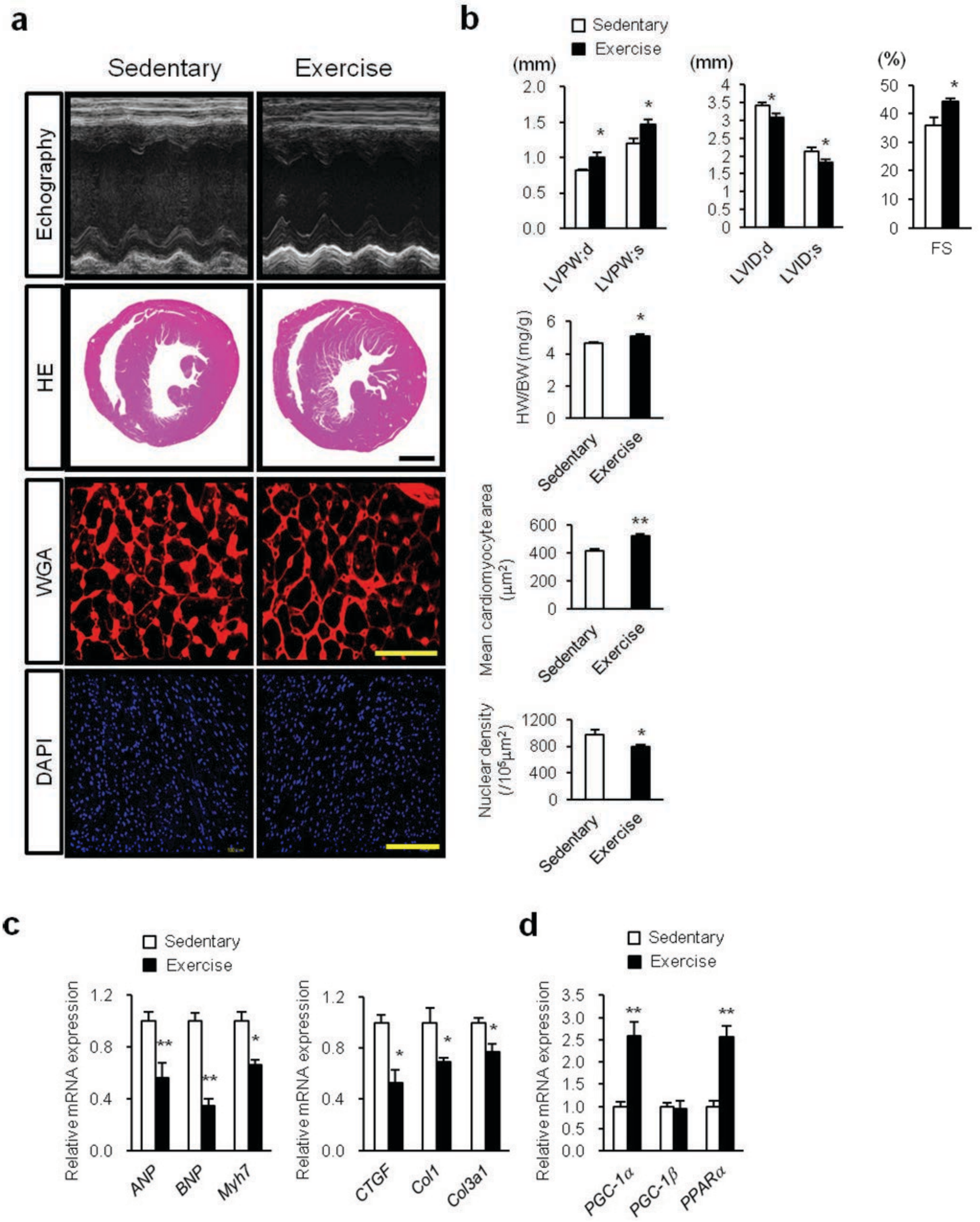

Supplementary Figure 4. Exercise training induces physiological cardiac hypertrophy and enhances cardiac function.

Comparison of hearts of WT mice maintained in sedentary conditions (Sedentary) or animals subjected to chronic endurance exercise training (Exercise). (**a**) Representative M-mode echocardiography recordings (top row),

HE-stained cross-sections of the heart mid-portion (second row; scale bar: 1 mm), left ventricle sections stained with WGA (third row; scale bar: 50 μm), and DAPI staining (bottom row; scale bar: 200 μm). (**b**) Shown are LVPW;d, LVPW;s LVID;d, LVID;s, %FS, heart weight per body weight (HW/BW) ratio, area of cardiomyocyte cross-sections, and nuclear density (n = 4 per group). (**c,d**) Relative expression of genes associated with heart failure (**c**, left), cardiac fibrosis (**c**, right), and energy metabolism (**d**) in hearts (n = 8 per each group). Levels in sedentary group were set at 1. Data are means ± SEM. Statistical significance was determined by Student's *t*-test. *$p < 0.05$, **$p < 0.01$ between groups.

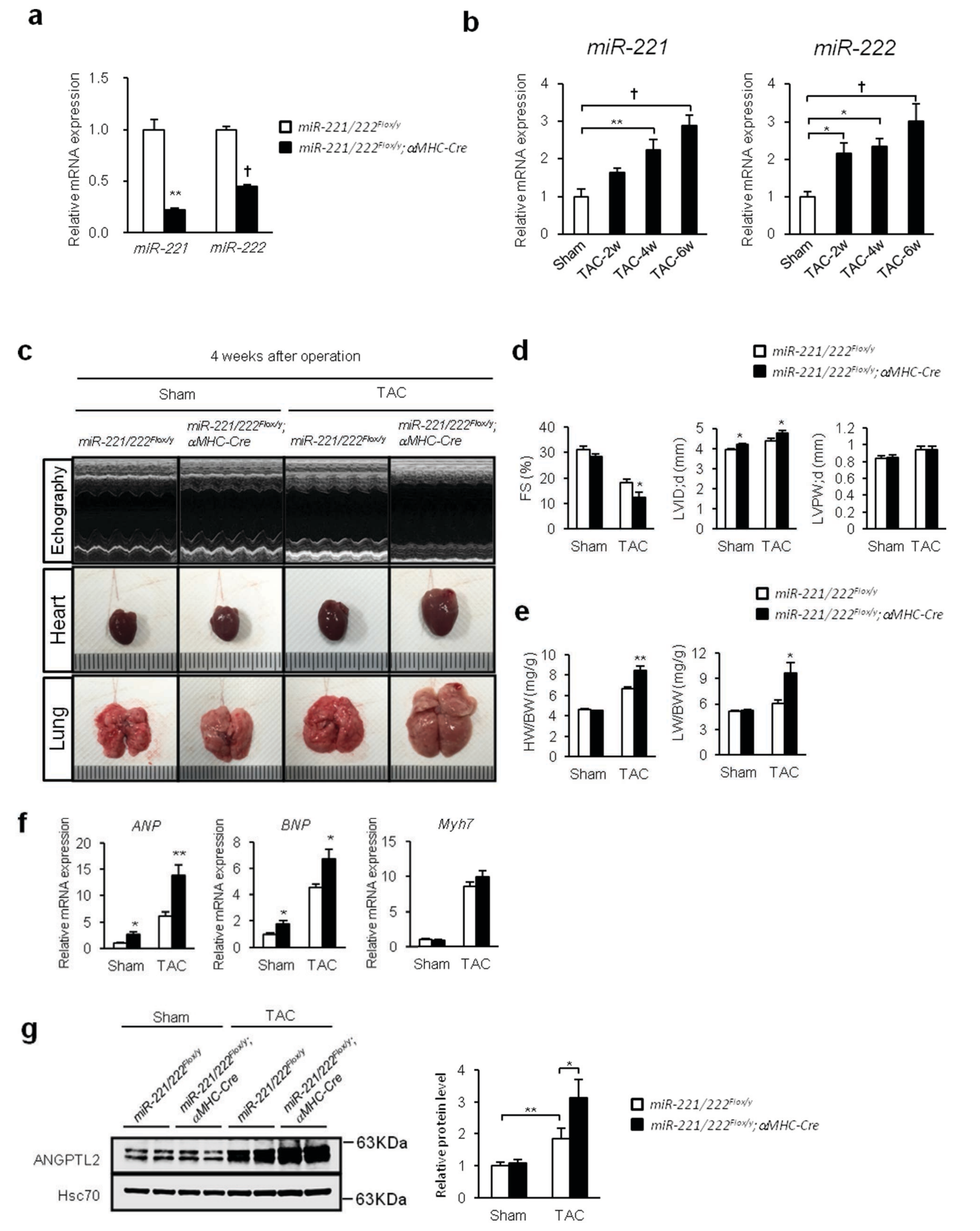

Supplementary Figure 5. Analysis of miR-221/222-deficient mice following exercise training or chronic pressure overload.

(**a**) Relative expression of *miR-221* and *miR-222* in heart of control *miR-221/222*$^{Flox/y}$ and knockout (KO) *miR-221/222*$^{Flox/y}$*;αMHC-Cre* mice (n = 3 per group). Levels in control mice were set to 1. (**b**) Relative expression of *miR-221* and *miR-222* in heart 2, 4, 6 weeks after TAC compared to corresponding sham-surgery groups (n = 4–6 per each group). Levels in sham mice were set to 1. (**c**) Representative M-mode echocardiography recordings (top

row) and gross appearance of whole heart (second row) and lung (third row) of *miR-221/222* KO and control mice 4 weeks after TAC or sham surgery. (**d-f**) Analysis of heart tissue from *miR-221/222* KO and control mice 4 weeks after sham or TAC surgery (n = 5–7 per each group). (**d**) FS (%), LVID;d, and LVPW;d. (**e**) Heart weight per body weight ratio and lung per body weight ratio. (**f**) Relative expression of genes associated HF. Levels in control mice in the sham group were set at 1. (**g**) Representative western blot (left) and quantification (right) of ANGPTL2 in heart of *miR-221/222* KO and control mice 4 weeks after TAC surgery (n = 5–8 per group). Hsc70 served as a loading control. Levels in control mice in the sham group were set at 1. Data are means ± SEM. Statistical significance was determined by Student's *t*-test (**a** and **d**–**f**) or one-way (**b**) or two-way (**g**) ANOVA. $^{*}p < 0.05$, $^{**}p < 0.01$, $^{\dagger}p < 0.001$ between groups.

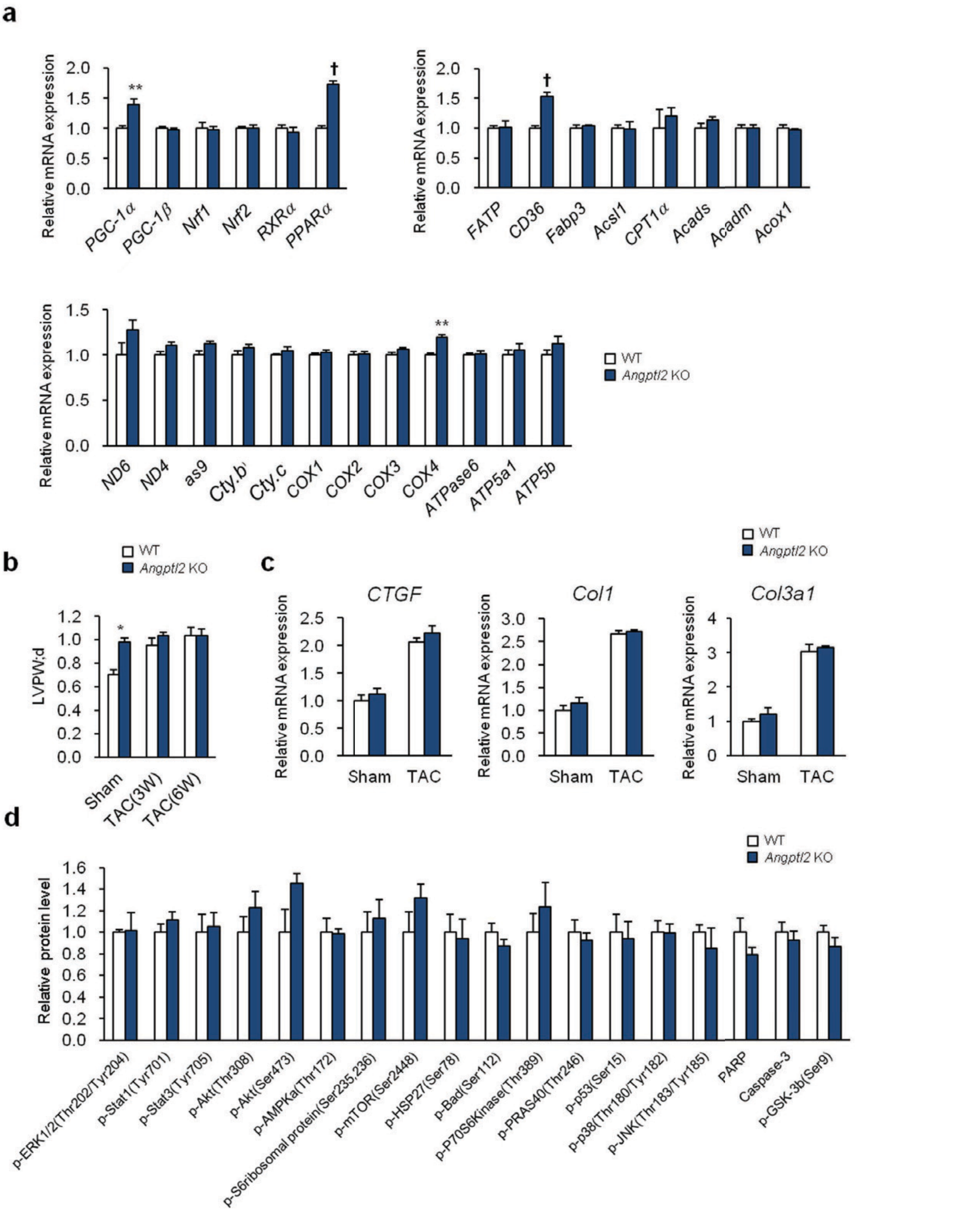

Supplementary Figure 6. Analysis of heart tissue of *Angptl2* KO mice.
(**a**) Relative expression of genes associated with energy metabolism (upper left), β-fatty acid oxidation (upper right), and mitochondrial biogenesis (lower left) in hearts of 6-week-old *Angptl2* KO mice and littermate WT mice (n = 5 per each group). Levels in WT mice were set to 1. (**b**) LVPW;d in *Angptl2* KO and WT littermate mice at indicated time points after TAC or sham surgery (n = 5–6 per group). (**c**) Relative expression of genes associated with cardiac fibrosis in heart of *Angptl2* KO and WT littermate mice 6 weeks after TAC or sham surgery (n = 5–6 per group). (**d**) Pathway scan analysis in heart tissues of *Angptl2* KO and WT littermate mice (n = 6–8 per group). Levels in WT mice were set at 1. Data are means ± SEM. Statistical significance was determined by Student's *t*-test. $^{**}p < 0.01$, $^{\dagger}p < 0.001$ between genotypes.

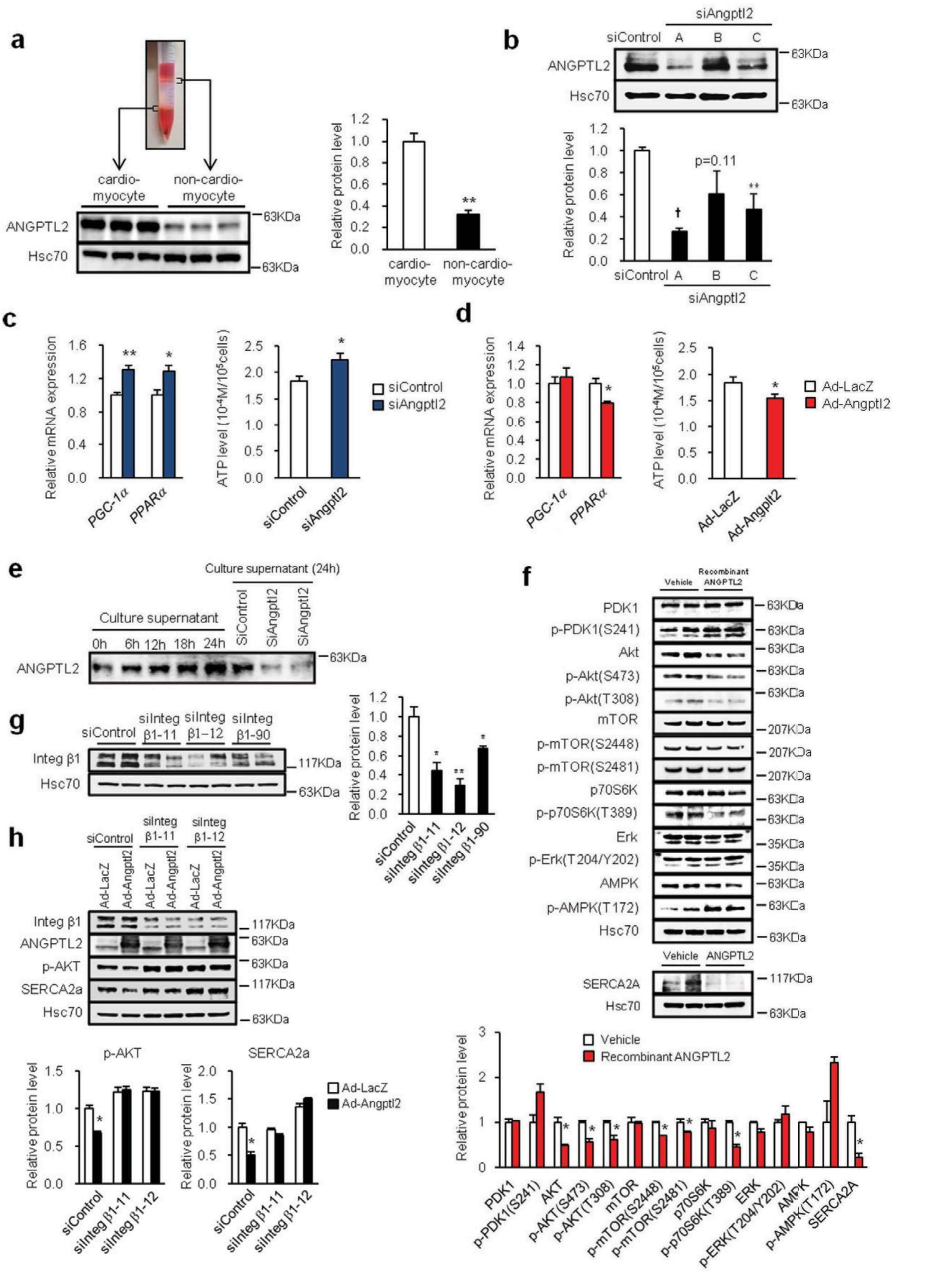

Supplementary Figure 7. Analysis of ANGPTL2-misexpressing or -knockdown NRCMs.
(**a**) Representative image of Percoll density gradient of neonatal rat heart tissue (upper left; see Material and Methods). Cardiomyocytes and non-cardiomyocytes were isolated from the indicated layers. Representative

western blot (lower left) and quantification (right) of ANGPTL2 protein in isolated cells indicated (n = 3). Hsc70 was used as a loading control. Levels in cardiomyocytes were set at 1. (**b**) Representative western blot (upper) and quantification (lower) of ANGPTL2 protein in NRCMs transfected with control (siControl) siRNA or Angptl2 (siAngptl2)-A, -B, and -C (n = 3). Hsc70 was used as a loading control. Levels in control siRNA cells were set at 1. (**c**) Relative expression of genes associated with energy metabolism (left) and quantitative ATP levels (right) in NRCMs transfected with control siRNA or Angptl2 siRNA-A (n = 8). Expression in siRNA control cells were set at 1. (**d**) Relative expression of genes associated with energy metabolism (left) and quantitative ATP levels (right) in NRCMs infected with recombinant adenovirus expressing Angptl2 (Ad-Angptl2) or control LacZ (Ad-LacZ) (n = 4). Expression in Ad-LacZ controls was set at 1. (**e**) Representative western blot of ANGPTL2 protein in 5 μl of culture supernatant from NRCMs or NRCMs transfected with either siAngptl2 or siControl at indicated times after changing the culture medium. (**f**) Representative western blot (upper) and quantification (bottom) of various signaling factors associated with AKT and SERCA2a in NRCMs treated with recombinant ANGPTL2 protein or vehicle. Hsc70 served as a loading control. Levels in the vehicle group were set at 1 (n = 3–6 per group). (**g**) Representative western blot (left) and quantification (right) of integrin β1 in NRCMs transfected with control siRNA (siControl) or three different siRNAs targeting β1 integrin (siInteg β1-11, β1-12 or β1-90) (n = 3–5). Hsc70 served as a loading control. (**h**) Representative western blot (upper) and quantification (bottom) of pAKT and SERCA2a protein in NRCMs treated with SiControl or SiInteg β1-11 or β1-12 and transduced with either control Ad-LacZ or Ad-Angptl2 (n = 3–4 per group). Hsc70 serve as a loading control. Levels in NRCMs infected with control Ad-LacZ in the siControl group were set at 1. Data are means ± SEM. Statistical significance was determined by Student's *t*-test (**a**, **c**, **d**, **f**, and **h**) or one-way ANOVA (**b** and **g**). $^{*}p < 0.05$, $^{**}p < 0.01$ between groups.

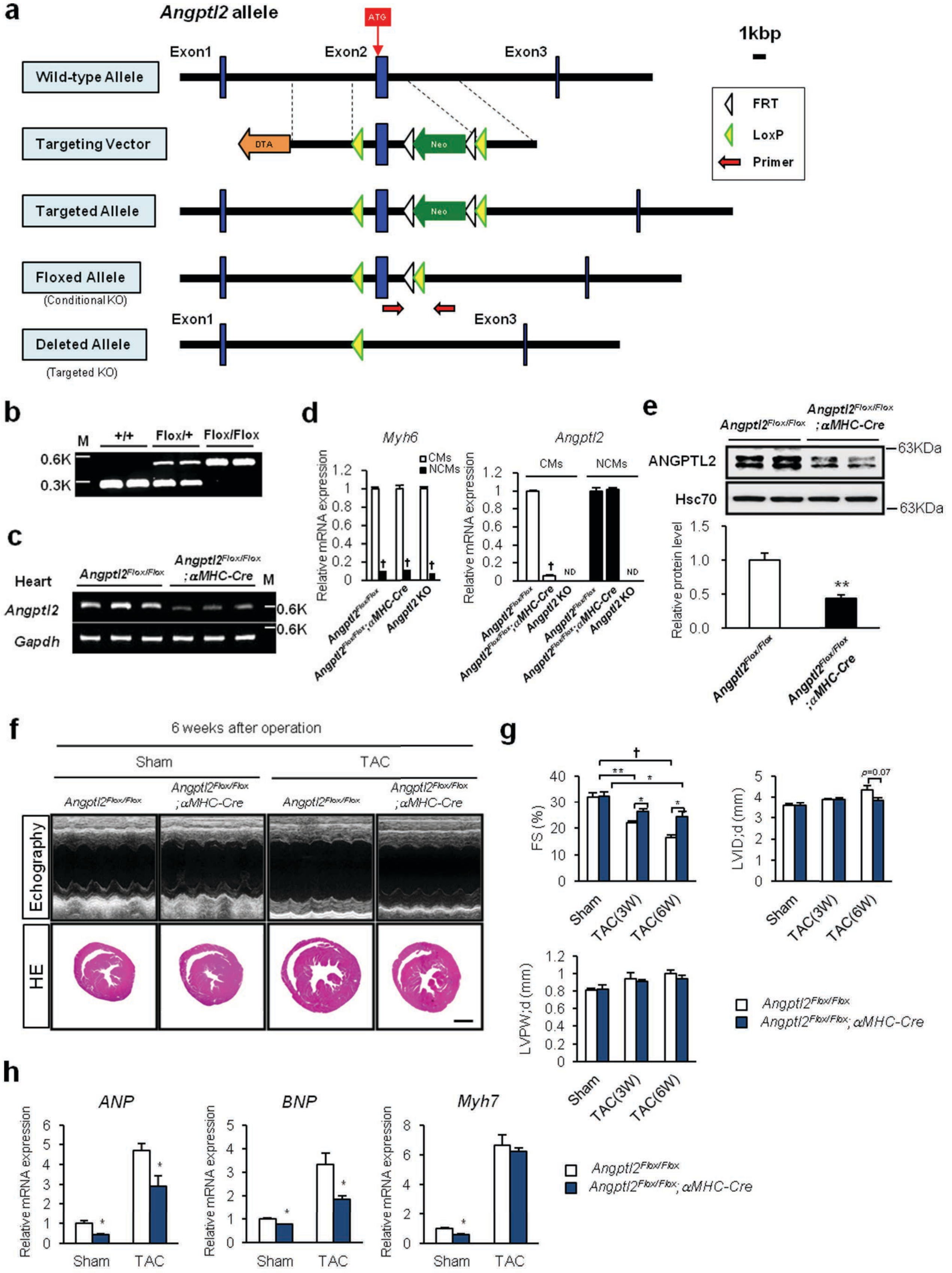

Supplementary Figure 8. Cardiomyocyte-specific conditional *Angptl2* knockout mice show resistance to HF development under chronic pressure overload.

(**a**) Schematic representation of the mouse *Angptl2* gene, the targeting vector, the floxed targeted allele, and the Cre-disrupted *Angptl2* gene. (**b**) Representative gels showing genotyping of wild-type (+/+), $Angptl2^{Flox/+}$ (Flox/+), and $Angptl2^{Flox/Flox}$ (Flox/Flox) mice using PCR using primers indicated in (**a**). (**c**) Semi-quantitative RT-PCR analysis of *Angptl2* and *Gapdh* expression in heart of control $Angptl2^{Flox/Flox}$ mice and $Angptl2^{Flox/Flox}$*; αMHC-Cre* mice. (**d**) Relative expression of *Myh6* and *Angptl2* in isolated cardiomyocytes (CMs) and non-cardiomyocyte (NCMs) from $Angptl2^{Flox/Flox}$, $Angptl2^{Flox/Flox}$*; αMHC-Cre* and *Angptl2* KO mice (n = 3 per group). Expression levels of *Myh6* and *Angptl2* in CMs and in $Angptl2^{Flox/Flox}$ mice respectively, were set at 1. (**e**) Representative western blot (upper) and quantification (bottom) of ANGPTL2 protein in heart of $Angptl2^{Flox/Flox}$ and $Angptl2^{Flox/Flox}$*; aMHC-Cre* (n = 6 per group) mice. Hsc70 served as loading control. Levels in control mice were set at 1. (**f**–**h**) Comparison of heart tissues from $Angptl2^{Flox/Flox}$ and $Angptl2^{Flox/Flox}$*; αMHC-Cre* mice 6 weeks after TAC or sham surgery. (n = 5 per group) (**f**) Representative M-mode echocardiography recordings (upper) and HE-stained cross-sections of midportion of the heart (bottom; scale bar: 1 mm). (**g**) FS (%), LVID;d, and LVPW;d. (**h**) Relative expression of genes associated with HF. Levels control mice in the sham group were set at 1. Data are means ± SEM. Statistical significance was determined by Student's *t*-test (**d**, **e**, **h**, and LVID;d, and LVPW;d in **g**) or two-way ANOVA (%FS in **g**). $*p < 0.05$, $**p < 0.01$, $^{\dagger}p < 0.001$ between groups.

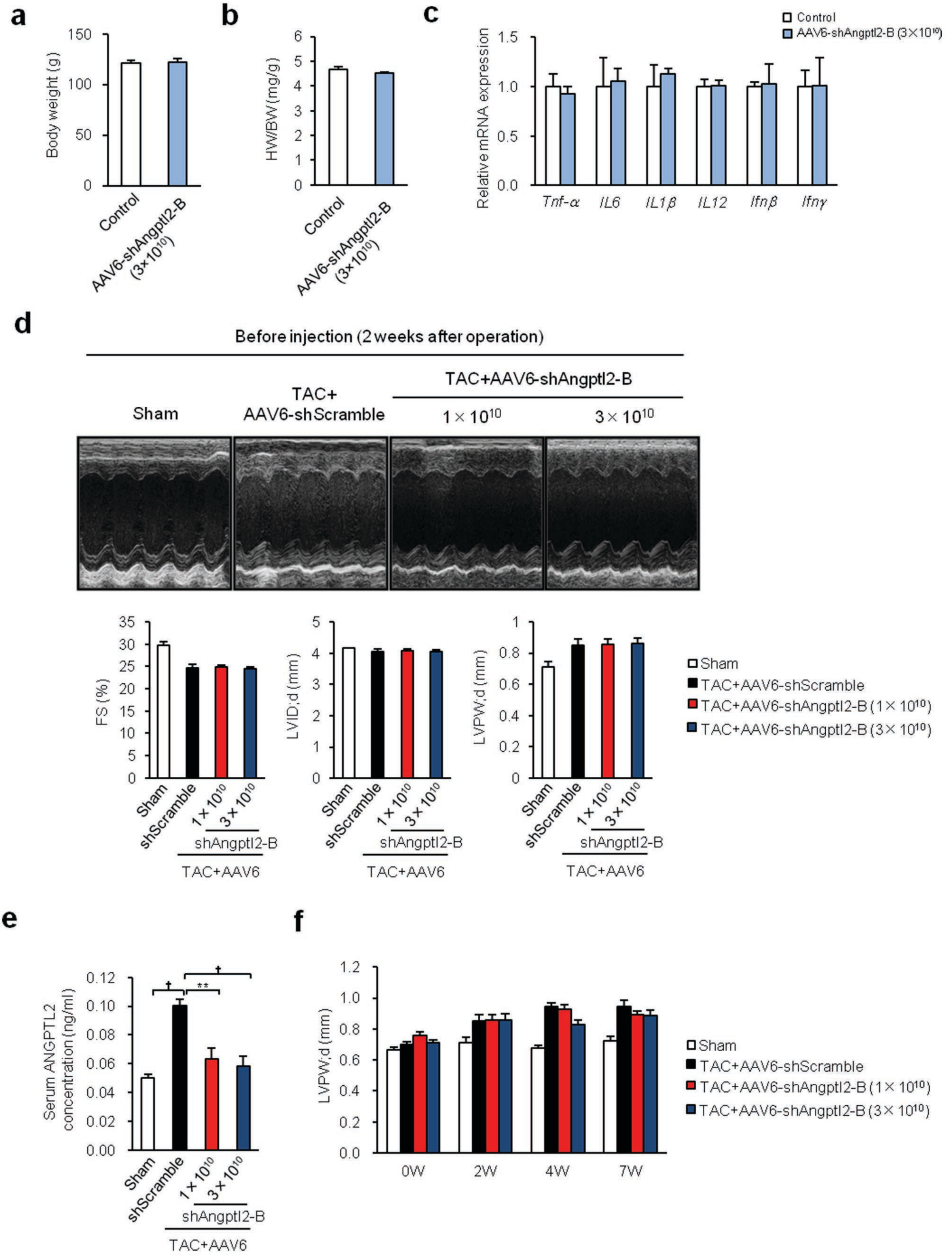

Supplementary Figure 9. The effects of injection of Angptl2 shRNA adeno-associated viral constructs on mouse heart tissues and serum ANGPTL2 levels.

(**a-c**) Assessment of potential side effects of AAV6-shAngptl2-B injection in mice (n = 5 per group). Body weight (**a**), heart weight per body weight (HW/BW) ratio (**b**), and relative expression of inflammatory genes in heart tissue

(**c**) of mice 4 weeks after AAV6-shAngptl2-B injection (3×10^{10} vg per mouse). Expression levels in non-treated control mice (Control) were set at 1. (**d**) Representative M-mode echocardiography recordings (upper) and associated parameters (%FS, LVID;d and LVPW;d) in mice before AAV6-shAngptl2-B injection (at 2 weeks after TAC or sham surgery) (n = 9–10 per group). (**e**) Serum ANGPTL2 concentration of mice injected with 3×10^{10} vg per mouse of AAV6-shScramble or 1×10^{10} or 3×10^{10} vg per mouse of AAV6-shAngptl2-B (n = 6–10 per group). (**f**) Comparison of LVPW;d among mice injected with 3×10^{10} vg/mouse of AAV6-shScramble or 1×10^{10} or 3×10^{10} vg per mouse of AAV6-shAngptl2-B (n = 6–10 per group). Data are means ± SEM. Statistical significance was determined by Student's *t*-test (**a**–**c**) or one-way ANOVA (**d**–**f**). $^{**}p < 0.01$, $^{\dagger}p < 0.001$ between groups.

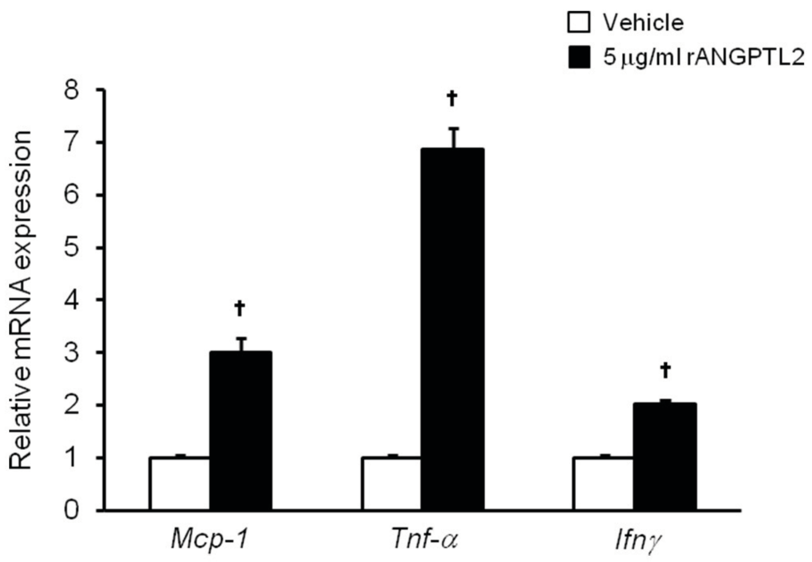

Supplementary Figure 10. ANGPTL2 induces pro-inflammatory gene expression in macrophages.
Relative expression of *Mcp-1, Tnf-α, and Ifnγ* in RAW264.7 cells treated with or without (Vehicle) 5.0 μg ml^{-1} recombinant ANGPTL2 (rANGPTL2) for 6 hours (n = 3–6). Expression in vehicle-treated cells was set at 1. Data are means ± SEM. Statistical significance was determined by Student's *t*-test. $^{\dagger}p < 0.001$ between groups.

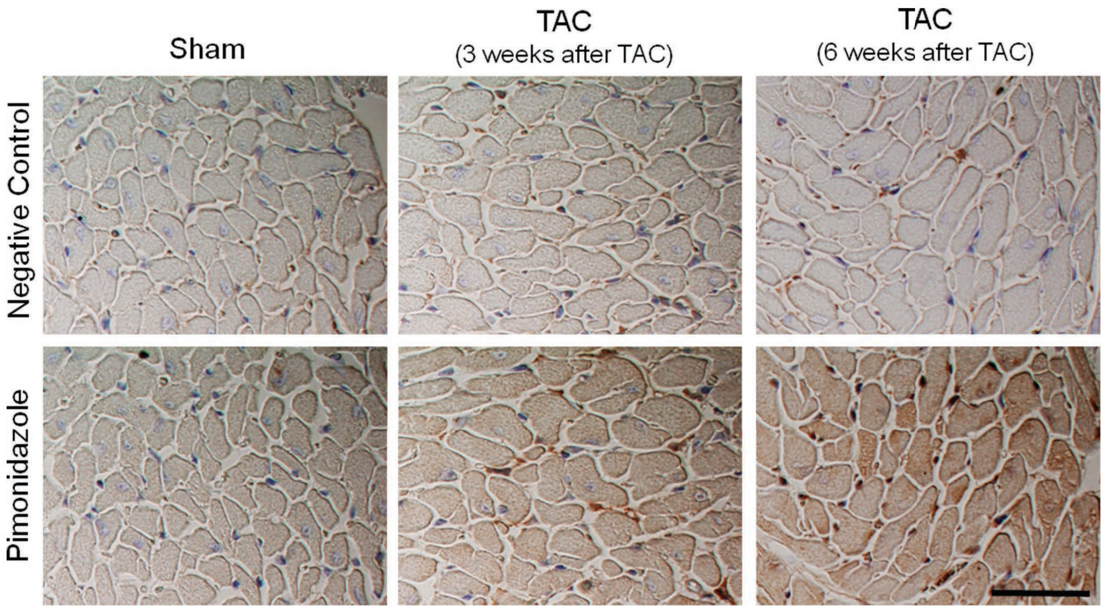

Supplementary Figure 11. TAC surgery induces cardiac hypoxia.
Immunohistochemical staining of pimonidazole adducts (lower row) in mouse heart tissues 3 or 6 weeks after TAC or sham surgery (n = 4–6 per group). Negative controls (upper row) were from the same location of the slide with no second antibody. (Scale bar: 50 μm).

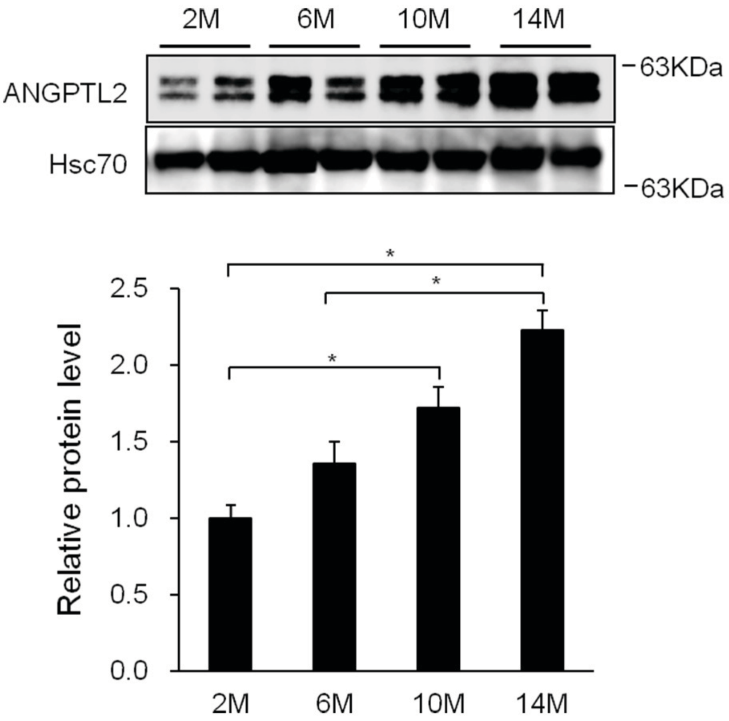

Supplementary Figure 12. Cardiac ANGPTL2 protein levels increase in aging mice.
Representative western blot (upper) and quantification (bottom) of ANGPTL2 in heart from 2-, 6-, 10-, or 14-month-old mice (n = 3 per group). Hsc70 served as a loading control. Levels in 2-month-old mice were set at 1. Data are means ± SEM. Statistical significance was determined by one-way ANOVA. *$p < 0.05$ between groups.

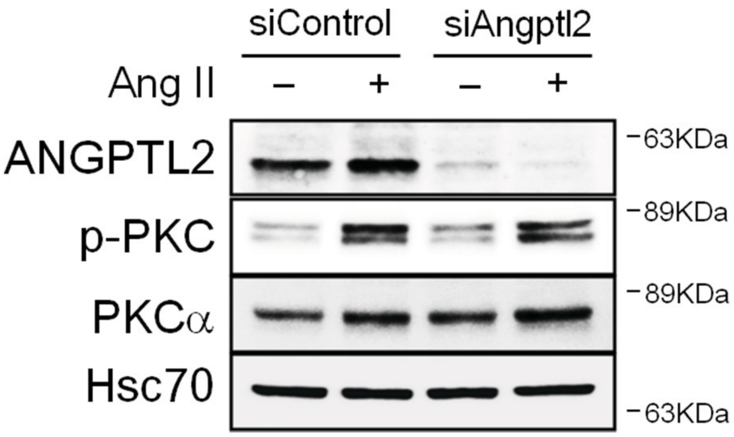

Supplementary Figure 13. ANGPTL2 does not mediate AT1A receptor recycling in cardiomyocytes. Representative western blot of phosphorylated PKC, PKCα, and Hsc70 in Angptl2 siRNA- or control siRNA-transfected NRCMs after 6 hours of Ang II (100 nM) treatment.

Fig. 1a

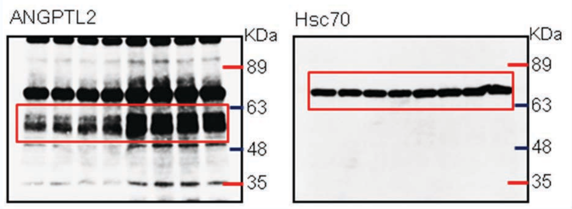

Fig. 1b

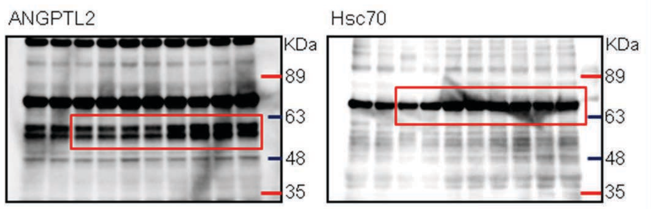

Fig. 1c

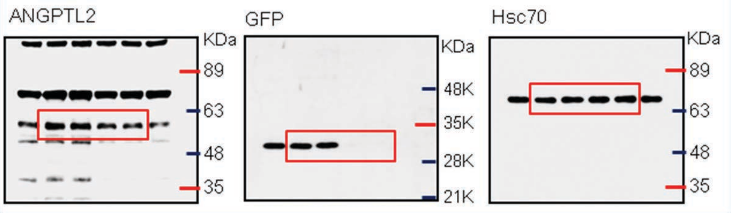

Fig. 1g

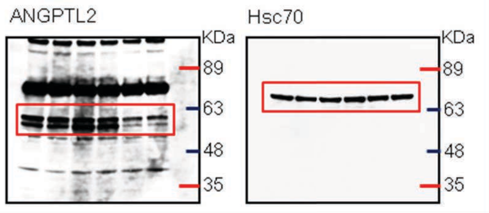

Fig. 4a

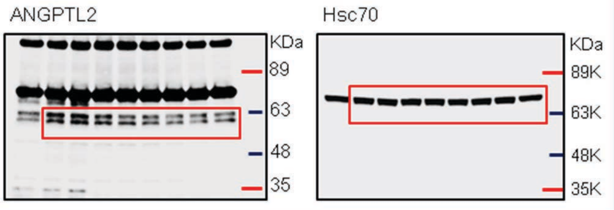

Fig. 4d

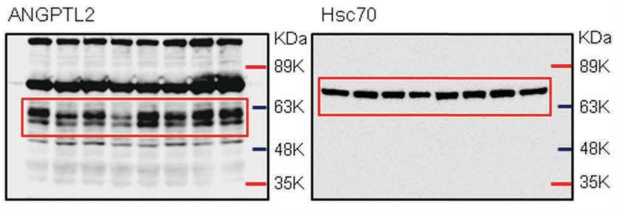

Fig. 6a (left)

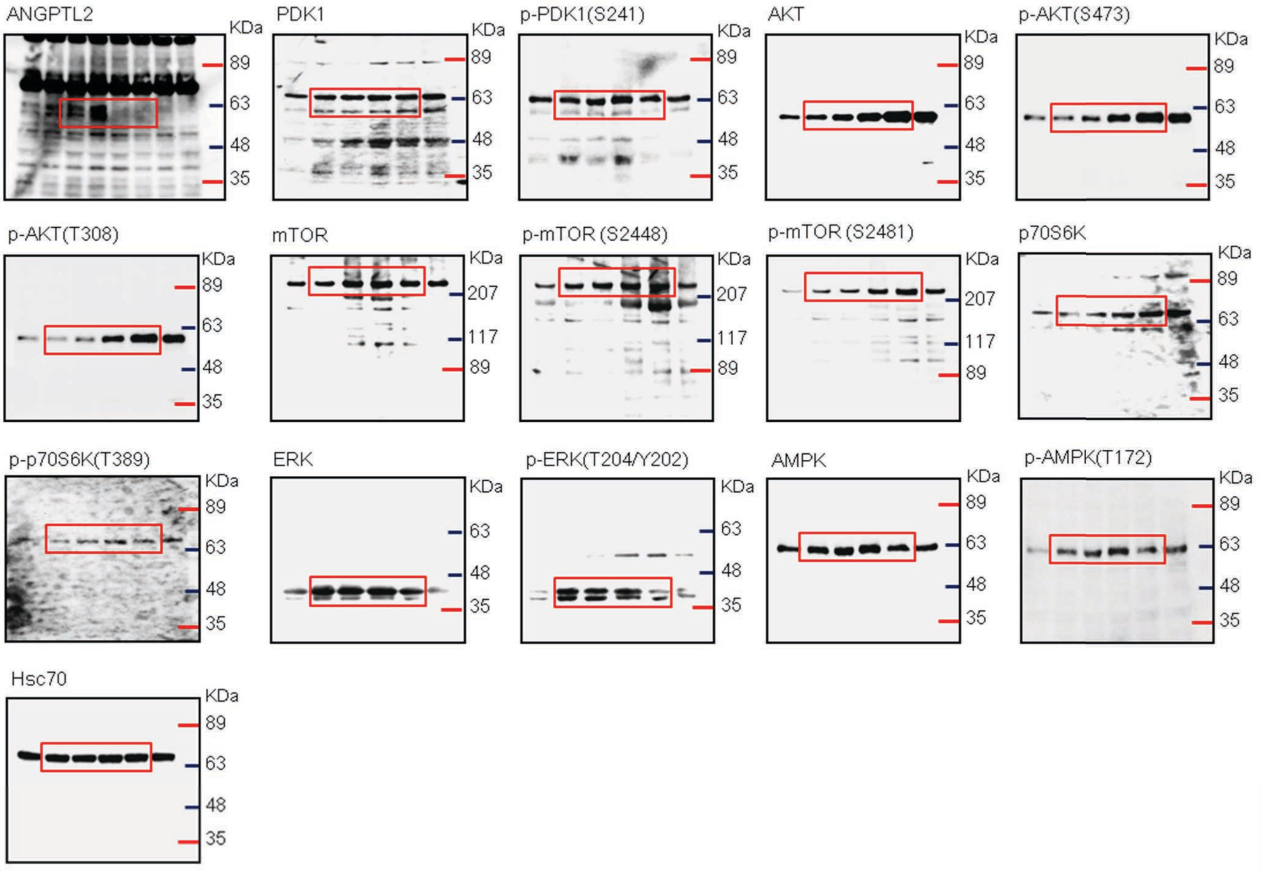

Fig. 6a (right)

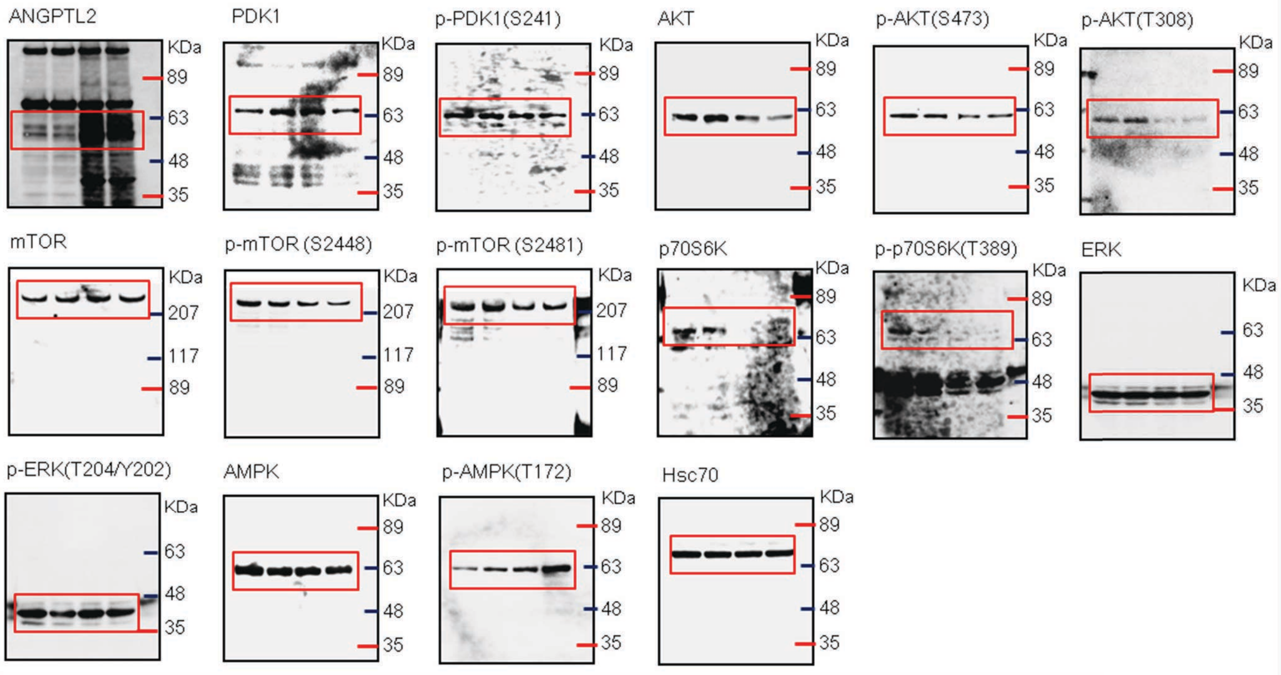

Fig. 6b (left)

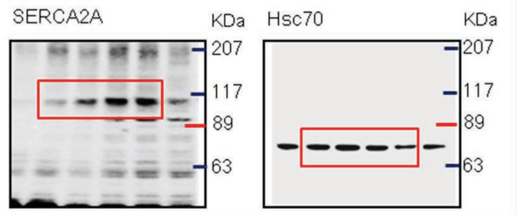

Fig. 6b (right)

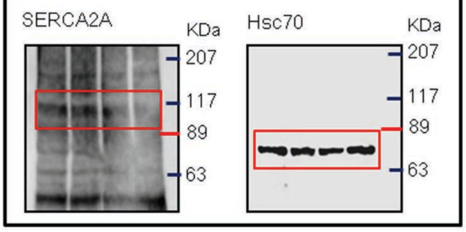

Fig. 6e (left)

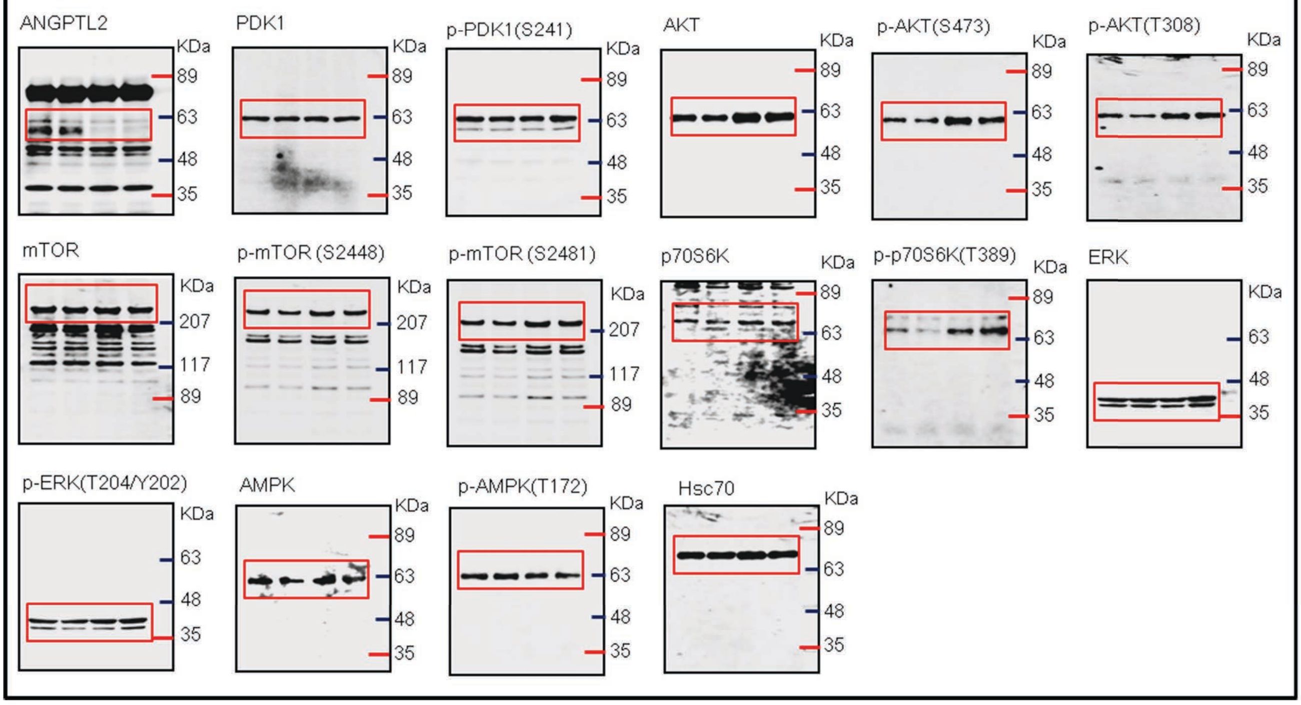

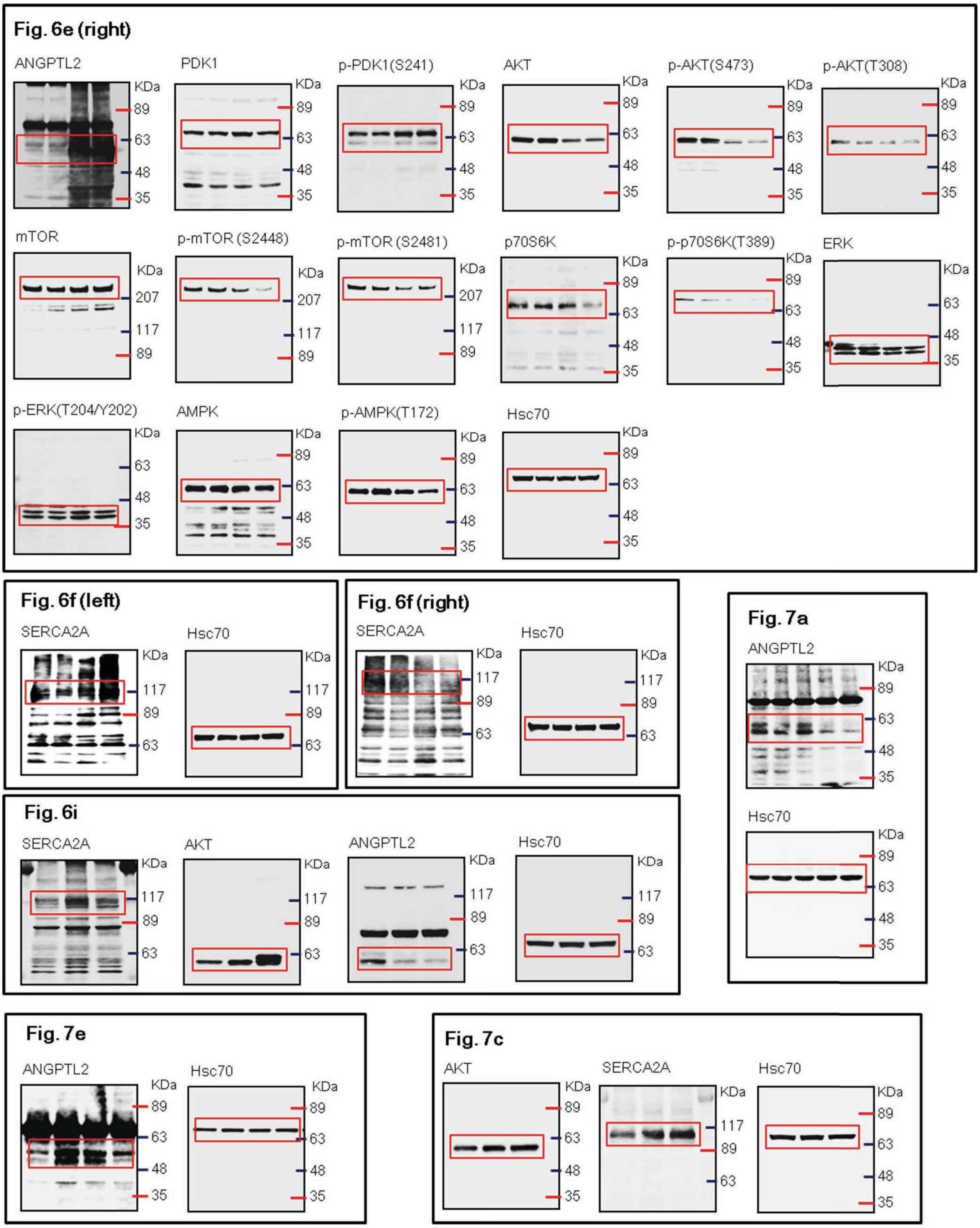
Fig. 6e (right)
ANGPTL2
PDK1
p-PDK1(S241)
AKT
p-AKT(S473)
p-AKT(T308)
mTOR
p-mTOR (S2448)
p-mTOR (S2481)
p70S6K
p-p70S6K(T389)
ERK
p-ERK(T204/Y202)
AMPK
p-AMPK(T172)
Hsc70
Fig. 6f (left)
SERCA2A
Hsc70
Fig. 6f (right)
SERCA2A
Hsc70
Fig. 7a
ANGPTL2
Hsc70
Fig. 6i
SERCA2A
AKT
ANGPTL2
Hsc70
Fig. 7e
ANGPTL2
Hsc70
Fig. 7c
AKT
SERCA2A
Hsc70
KDa

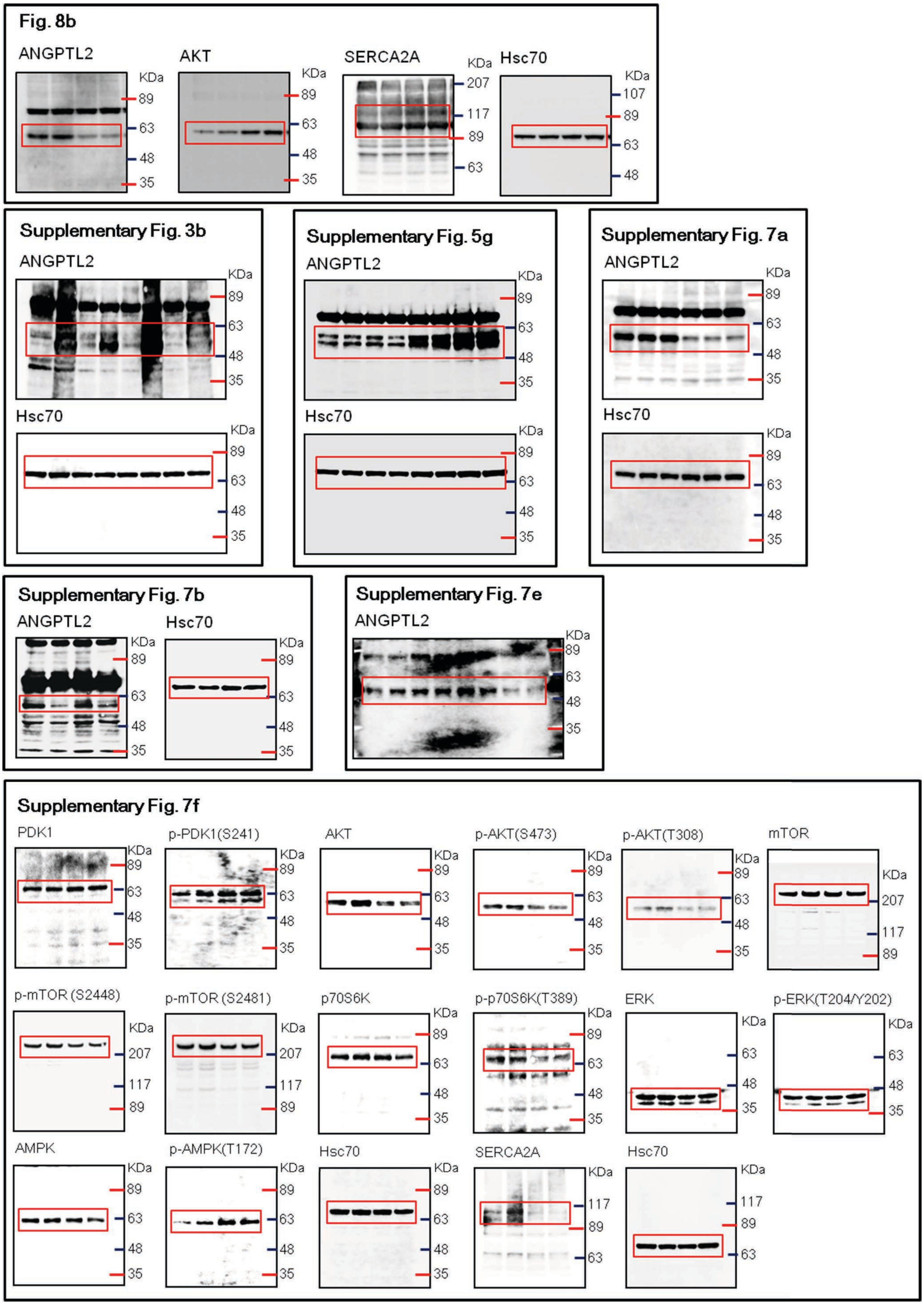

Fig. 8b
ANGPTL2
KDa 89 63 48 35
AKT
KDa 89 63 48 35
SERCA2A
KDa 207 117 89 63
Hsc70
KDa 107 89 63 48
Supplementary Fig. 3b
ANGPTL2
KDa 89 63 48 35
Hsc70
KDa 89 63 48 35
Supplementary Fig. 5g
ANGPTL2
KDa 89 63 48 35
Hsc70
KDa 89 63 48 35
Supplementary Fig. 7a
ANGPTL2
KDa 89 63 48 35
Hsc70
KDa 89 63 48 35
Supplementary Fig. 7b
ANGPTL2
KDa 89 63 48 35
Hsc70
KDa 89 63 48 35
Supplementary Fig. 7e
ANGPTL2
KDa 89 63 48 35
Supplementary Fig. 7f
PDK1
KDa 89 63 48 35
p-PDK1(S241)
KDa 89 63 48 35
AKT
KDa 89 63 48 35
p-AKT(S473)
KDa 89 63 48 35
p-AKT(T308)
KDa 89 63 48 35
mTOR
KDa 207 117 89
p-mTOR (S2448)
KDa 207 117 89
p-mTOR (S2481)
KDa 207 117 89
p70S6K
KDa 89 63 48 35
p-p70S6K(T389)
KDa 89 63 48 35
ERK
KDa 63 48 35
p-ERK(T204/Y202)
KDa 63 48 35
AMPK
KDa 89 63 48 35
p-AMPK(T172)
KDa 89 63 48 35
Hsc70
KDa 89 63 48 35
SERCA2A
KDa 117 89 63
Hsc70
KDa 117 89 63

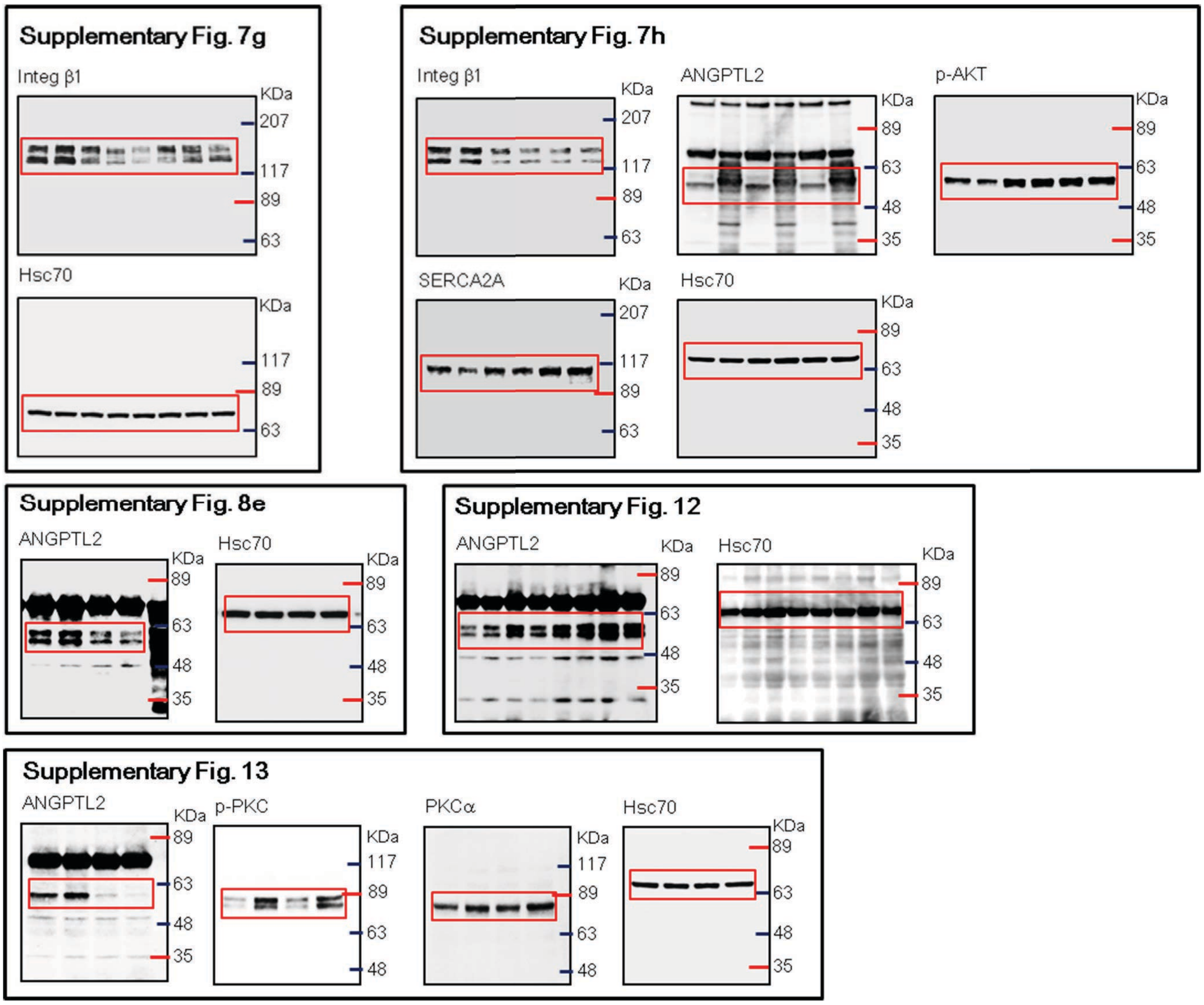

Supplementary Figure 14. Full scans of all immunoblots.
Red boxes indicate the cropped region of the corresponding figures.

Supplementary Table 1.

DCM Patient Clinical Characteristics

	Activated Group (ANGPTL2 levels: (Cs-Ao)*100/Ao > 35%)	Non-Activated Group (ANGPTL2 levels: (Cs-Ao)*100/Ao ≤ 35%)	*p*
**Number**	23	35	
**Age**	59.17 ± 2.50	51.89 ± 2.10	<0.05
**Sex (male / female)**	15 / 8	25 / 10	
**NYHA (I / II / III)**	12 / 9 / 2	14 / 18 / 3	
**BNP (pg/ml)**	217.04 ± 43.63	178.65 ± 50.58	n.s.
**Cardiac Catheterization Analysis**			
**ANGPTL2 levels in Ao (ng/ml)**	2.41 ± 0.17	2.50 ± 0.16	n.s.
**ANGPTL2 levels in Cs (ng/ml)**	4.56 ± 0.30	2.09 ± 0.18	<0.01
**Step-up of ANGPTL2 levels from Ao to Cs ((Cs-Ao)*100/Ao (%))**	97.62 ± 11.06	-18.18 ± 4.05	<0.01
**EF (%)**	32.08 ± 2.14	31.54 ± 1.91	n.s.
**CI (L/min/m^2)**	2.56 ± 0.12	2.75 ± 0.11	n.s.
**PCWP (mmHg)**	11.59 ± 0.96	12.70 ± 1.08	n.s.
**Ultrasonic Echocardiographic Analysis**			
**LVDd (mm)**	59.09 ± 1.16	64.57 ± 1.51	<0.01
**LAD (mm)**	40.61 ± 1.72	41.07 ± 1.58	n.s.
**LVMI (g/m^2)**	153.46 ± 8.22	179.38 ± 12.73	n.s.
**%FS (%)**	18.54 ± 1.43	16.03 ± 1.00	n.s.

Values are mean ± SEM or number (n) and percentage (%) for categorical variables. DCM = dilated cardiomyopathy; Ao = aortic root; Cs = coronary sinus; EF = ejection fraction; CI = cardiac index; PCWP = pulmonary capillary wedge pressure; LVDd = left ventricular end-diastolic dimension; LAD = left atrial dimension; LVMI = left ventricular mass index; FS = fractional shortening; n.s.= not significant.

Supplementary Table 2.

Primer Sequences Used in Quantitative RT-PCR (mouse)

Gene		Sequences
Rps18	Forward	TTCTGGCCAACGGTCTAGACAAC
	Reverse	CCAGTGGTCTTGGTGTGCTGA
Angptl2	Forward	GGAGGTTGGACTGTCATCCAGAG
	Reverse	GCCTTGGTTCGTCAGCCAGTA
ANP	Forward	GAGAGACGGCAGTGCTTCTAGGC
	Reverse	CGTGACACACCACAAGGGCTTAGG
BNP	Forward	AGGCGAGACAAGGGAGAACA
	Reverse	GGAGATCCATGCCGCAGA
Myh7	Forward	CGGACCTTGGAAGACCAGAT
	Reverse	GACAGCTCCCCATTCTCTGT
CTGF	Forward	CAAAGCAGCTGCAAATACCA
	Reverse	GGCCAAATGTGTCTTCCAGT
Col1	Forward	GAGCGGAGAGTACTGGATCGA
	Reverse	CTGACCTGTCTCCATGTTGCA
Col3a	Forward	CAACCAGTGCAAGTGACCAA
	Reverse	GCACCATTGAGACATTTTGAAG
PGC-1α	Forward	CCGTAAATCTGCGGGATGATG
	Reverse	CAGTTTCGTTCGACCTGCGTAA
PGC-1β	Forward	GTGCCAGGTGCTGACGAGAA
	Reverse	AGTGTATCTGGGCCAACGGAAG
Nrf1	Forward	TTGCCCAAGTGAATTACTCTGCTG
	Reverse	TGCAGGACAGTCTGAGCCATC
Nrf2	Forward	TTGGCAGAGACATTCCCATTTGTA
	Reverse	AGTCATGGCTGCCTCCAGAGA
RXRα	Forward	CCATGCCTTAGCTGGGATGTG
	Reverse	TTCCCGGTGCGTTAAACATTC
PPARα	Forward	ACGCTCCCGACCCATCTTTAG
	Reverse	TCCATAAATCGGCACCAGGAA

Gene		Sequences
FATP	Forward	GCAGCATTGCCAACATGGAC
	Reverse	GTGTCCTCATTGACCTTGACCAGA
CD36	Forward	GATGGCCTTACTTGGGATTGGA
	Reverse	GGCTTTACCAAAGATGTAGCCAGTG
Fabp3	Forward	TGGCTAGCATGACCAAGCCTACTAC
	Reverse	GTTCCACTTCTGCACATGGATGA
Acsl1	Forward	TTTGCCTGCAGCGAGTGTG
	Reverse	GCCCTCGACTATCCCTATGGTAAGA
CPT1α	Forward	GCCATGATGGACCCCACAAC
	Reverse	CCAGATACTTGGACACCACATAGAGG
CPT1β	Forward	GAGACAGGACACTGTGTGGGTGA
	Reverse	AGTGCCTTGGCTACTTGGTACGAG
Acads	Forward	AAGTTTGGATCCGCACAGCAG
	Reverse	CAAGCTTTGGTGCCGTTGAG
Acadm	Forward	CGAGTATGTTATCAACGGCCAGAA
	Reverse	GCGGGTACTTTAGGATCTGGGTTAG
Acox1	Forward	AAGATGGATCCTAAGCCAGCTGAA
	Reverse	CAGCTTACCACAAAGCCAGCTACTC
ND6	Forward	AGGTGAAGGCTTTAATGCTAACCC
	Reverse	GGTCGCAGTTGAATGCTGTGT
ND4	Forward	CATCACTCCTATTCTGCCTAGCAA
	Reverse	TCCTCGGGCCATGATTATAGTAC
as9	Forward	CCCGGGCCAGCTTACCT
	Reverse	GCTGCACTGCTTTCCTGATAGA
Cyt.b	Forward	GCCACCTTGACCCGATTCT
	Reverse	TTGCTAGGGCCGCGATAAT
Cyt.c	Forward	GGCTGCTGGATTCTCTTACACA
	Reverse	CCAAATACTCCATCAGGGTATCCT
Cox1	Forward	TTTTCAGGCTTCACCCTAGATGA
	Reverse	GAAGAATGTTATGTTTACTCCTACGAATATG

Gene		Sequences
Cox2	Forward	CCATCCCAGGCCGACTAAA
	Reverse	TTTCAGAGCATTGGCCATAGAA
Cox3	Forward	CGGAAGTATTTTTCTTTGCAGGAT
	Reverse	CAGCAGCCTCCTAGATCATGTG
Cox4	Forward	TGCAGACCAAGCGAATGCT
	Reverse	TAGTCCCACTTGGCGGAGAA
ATPase6	Forward	GGCTCCCGACACAAACTAAAAAG
	Reverse	TGGAATTAGTGAAATTGGAGTTCCT
ATP5a1	Forward	ATGTGTCCGCTTACATTCCAACAA
	Reverse	GATCCGACACGGGACACAGA
ATP5b	Forward	ACATGGGCACAATGCAGGAA
	Reverse	GTCAGGTCATCAGCAGGCACA
Tnf-α	Forward	AAGCCTGTAGCCCACGTCGTA
	Reverse	GGCACCACTAGTTGGTTGTCTTTG
IL6	Forward	CCACTTCACAAGTCGGAGGCTTA
	Reverse	GCAAGTGCATCATCGTTGTTCATAC
IL1β	Forward	TCCAGGATGAGGACATGAGCAC
	Reverse	GAACGTCACACACCAGCAGGTTA
IL12	Forward	TACTAGAGAGACTTCTTCCACAACAAGAG
	Reverse	TCTGGTACATCTTCAAGTCCTCATAGA
Ifnβ	Forward	GCAGCTGAATGGAAAGATCA
	Reverse	TGGCAAAGGCAGTGTAACTC
Ifnγ	Forward	CGGCACAGTCATTGAAAGCCTA
	Reverse	GTTGCTGATGGCCTGATTGTC
Mcp-1	Forward	GCATCCACGTGTTGGCTCA
	Reverse	CTCCAGCCTACTCATTGGGATCA

Supplementary Table 3.

Primer Sequences Used in Quantitative RT-PCR (Rat)

**Gene**		**Sequences**
Rps18	Forward	CGCCGCTAGAGGTGAAATTC
	Reverse	CCAGTCGGCATCGTTTATGG
Angptl2	Forward	TTACCTGGCACAACGGCAAAC
	Reverse	ACCACCATCCTCCCTTCTGATAG
ANP	Forward	GGGGGTAGGATTGACAGGAT
	Reverse	CTCCAGGAGGGTATTCACCA
BNP	Forward	GACGGGCTGAGGTTGTTTTA
	Reverse	ACTGTGGCAAGTTTGTGCTG
MYH7	Forward	GAGCCTCCAGAGTTTGCTGAAGGA
	Reverse	TTGGCACGGACTGCGTCATC
Serca2a	Forward	ATTGTTCGAAGTCTGCCTTCTGTGG
	Reverse	CATAGGTTGATCCAGTTATGGTAAA
PGC-1α	Forward	ACCCACAGGATCAGAACAAACC
	Reverse	GACAAATGCTCTTTGCTTTATTGC
PPARα	Forward	TGGTGGACCTCCGGCA
	Reverse	TCTTCTTGATGACCTGCACGA

Supplementary Table 4.

Primer Sequences Used in Quantitative RT-PCR (Human)

**Gene**		**Sequences**
RPS18	Forward	TTTGCGAGTACTCAACACCAACATC
	Reverse	GAGCATATCTTCGGCCCACAC
ANGPTL2	Forward	GCCACCAAGTGTCAGCCTCA
	Reverse	TGGACAGTACCAAACATCCAACATC
PGC-1α	Forward	AAGTGTGGAACTCTCTGGAACTG
	Reverse	GGGTTATCTTGGTTGGCTTTATG
PPARα	Forward	CTGGAAGCTTTGGCTTTACG
	Reverse	GATAAGTCACCGAGGAGGGG
